# Chromosome-level draft genome of a diploid plum (*Prunus salicina*)

**DOI:** 10.1093/gigascience/giaa130

**Published:** 2020-12-10

**Authors:** Chaoyang Liu, Chao Feng, Weizhuo Peng, Jingjing Hao, Juntao Wang, Jianjun Pan, Yehua He

**Affiliations:** Key Laboratory of Biology and Germplasm Enhancement of Horticultural Crops in South China, Ministry of Agriculture, South China Agricultural University, 483 Wushan Road, Guangzhou 510642, China; Key Laboratory of Plant Resources Conservation and Sustainable Utilization, South China Botanical Garden, Chinese Academy of Sciences, 1190 Tianyuan Road, Guangzhou 510650, China; Key Laboratory of Biology and Germplasm Enhancement of Horticultural Crops in South China, Ministry of Agriculture, South China Agricultural University, 483 Wushan Road, Guangzhou 510642, China; Maoming Branch, Guangdong Laboratory for Lingnan Modern Agriculture, 5 Youchengliu Road, Maoming 525000, China; Key Laboratory of Biology and Germplasm Enhancement of Horticultural Crops in South China, Ministry of Agriculture, South China Agricultural University, 483 Wushan Road, Guangzhou 510642, China; Maoming Branch, Guangdong Laboratory for Lingnan Modern Agriculture, 5 Youchengliu Road, Maoming 525000, China; Key Laboratory of Biology and Germplasm Enhancement of Horticultural Crops in South China, Ministry of Agriculture, South China Agricultural University, 483 Wushan Road, Guangzhou 510642, China; Maoming Branch, Guangdong Laboratory for Lingnan Modern Agriculture, 5 Youchengliu Road, Maoming 525000, China; Agricultural Technology Extension Center of Conghua District, 468 Tianlu Road, Guangzhou 510900, Guangdong Province, China; Key Laboratory of Biology and Germplasm Enhancement of Horticultural Crops in South China, Ministry of Agriculture, South China Agricultural University, 483 Wushan Road, Guangzhou 510642, China; Maoming Branch, Guangdong Laboratory for Lingnan Modern Agriculture, 5 Youchengliu Road, Maoming 525000, China

**Keywords:** Prunus, Plum, Genome, Chromosome-level

## Abstract

**Background:**

Plums are one of the most economically important Rosaceae fruit crops and comprise dozens of species distributed across the world. Until now, only limited genomic information has been available for the genetic studies and breeding programs of plums. *Prunus salicina*, an important diploid plum species, plays a predominant role in modern commercial plum production. Here we selected *P. salicina* for whole-genome sequencing and present a chromosome-level genome assembly through the combination of Pacific Biosciences sequencing, Illumina sequencing, and Hi-C technology.

**Findings:**

The assembly had a total size of 284.2 Mb, with contig N50 of 1.78 Mb and scaffold N50 of 32.32 Mb. A total of 96.56% of the assembled sequences were anchored onto 8 pseudochromosomes, and 24,448 protein-coding genes were identified. Phylogenetic analysis showed that *P. salicina* had a close relationship with *Prunus mume* and *Prunus armeniaca*, with *P. salicina* diverging from their common ancestor ∼9.05 million years ago. During *P. salicina* evolution 146 gene families were expanded, and some cell wall–related GO terms were significantly enriched. It was noteworthy that members of the DUF579 family, a new class involved in xylan biosynthesis, were significantly expanded in *P. salicina*, which provided new insight into the xylan metabolism in plums.

**Conclusions:**

We constructed the first high-quality chromosome-level plum genome using Pacific Biosciences, Illumina, and Hi-C technologies. This work provides a valuable resource for facilitating plum breeding programs and studying the genetic diversity mechanisms of plums and *Prunus* species.

Plums are one of the most economically important Rosaceae fruit crops and are produced throughout the world. Roughly 12.6 million tons of plums (including sloes) are produced per year [1], and the fruits are widely used for fresh consumption and processing such as canning and beverages [2]. There are 19–40 species of plums distributed across Asia, Europe, and North America. Plums have great diversity and are considered to be a link between the major subgenera in the genus *Prunus* [3].

*Prunus salicina*, commonly called the Japanese plum or Chinese plum, is an important diploid (2x = 2n = 16) plum species that predominates in the modern commercial production of plums (Fig. 1). *P. salicina* originates in China and its fruits are mostly used for fresh consumption for their characteristic taste [4]. Cultivars of *P. salicina* have wide variability in phenology, fruit size and shape, flavor, firmness, aroma, texture, phenolic composition, antioxidant activity, and both skin and pulp color [5].

**Figure 1:**
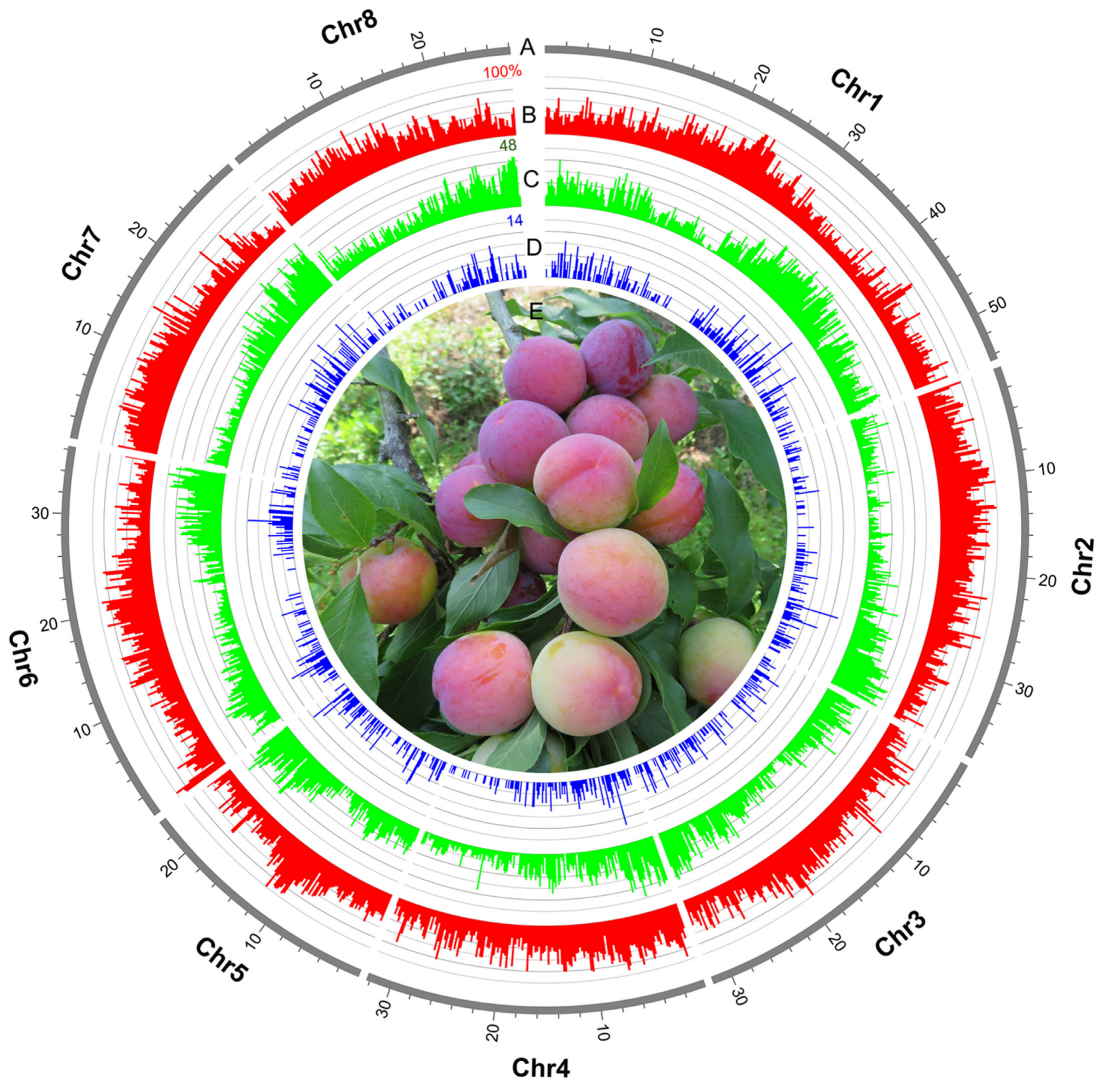
The genome and photograph of *P. salicina*. (A) Landscape of the *P. salicina* genome, comprising 8 pseudochromosomes that cover ∼96.56% of assembly. Concentric circles, from outermost to innermost, show (B) TE percentage (red), (C) gene density (green), and (D) density of duplicates resulting from tandem duplications (blue). (E) Photograph of *P. salicina*.

However, the genetic and genomic information for *P. salicina*, as well as most plum species, has been scarce [6]. The availability of a fully sequenced and annotated genome will help to measure and characterize the genetic diversity and determine how this diversity relates to the tremendous phenotypic diversity among plum cultivars. The genomic information is essential to support many of the studies involved in fundamental questions about plum biology and genetics. Moreover, genome-based tools could be developed to improve plum breeding work, which has typically been hindered by the high degree of heterozygosity, self-incompatibility, and long juvenile stage [3, 6, 7].

The fruit firmness, one of the most important indices of plum quality, is closely associated with cell wall composition [3]. Xylan is a major component of secondary cell walls [8], and xylan metabolism is involved in various aspects of plant growth and development such as fruit ripening and softening [9]. According to previous studies, the plum species present more xylose (the main component of xylan) compared with other *Prunus* species, and plums have been regarded as one of the richest natural sources of xylitol [10, 11]. The relatively high levels of xylan-related metabolites may be associated with the distinct mechanisms of the xylan metabolism in plums, and the available plum genomic information will be helpful to better elucidate the mechanism at molecular level.

Genome resources are already available for a number of Rosaceae fruit crops [12], including apple [13–16], peach [17], pear [18–21], strawberry [22, 23], almond [24, 25], black raspberry [26], sweet cherry [27, 28], apricot [29, 30], loquat [31], and *Prunus mume* [32]. However, whole-genome sequencing and chromosome-level assembly for plums have not been reported until now. In this study, *P. salicina* was selected for the whole-genome sequencing as a genomic reference. A high-quality chromosome-level *de novo* genome assembly of *P. salicina* was generated using an integrated strategy that combines Pacific Biosciences (PacBio) sequencing, Illumina sequencing, and Hi-C technology. The assembly has a total size of 284.2 Mb with contig N50 of 1.8 Mb and scaffold N50 of 32.3 Mb, and almost all (96.56%) of the assembled sequence was anchored onto 8 pseudochromosomes. The availability of the high-quality chromosome-scale genome sequences not only provides fundamental knowledge regarding plum biology but also presents a valuable resource for genetic diversity analysis and breeding programs of plums and other *Prunus* crops.

## Methods

### Sample collection

The *Prunus salicina* Lindl. cv. “Sanyueli,” a Japanese plum landrace originating from southern China, was selected for genome sequencing and assembly. Sanyueli has a cultivation history of >200 years and many distinctive characteristics, including early maturation, high yield, and low chilling requirements. The Sanyueli samples were kept at the Horticultural Germplasm Conservation Center of South China Agricultural University for breeding and research in Guangzhou, Guangdong Province, China (113°22'4" N, 23°9'5" E). Total genomic DNA was extracted from fresh young leaves of 5-year-old *P. salicina* tree using the CTAB method [33]. Samples from a total of 6 tissues, including leaf, flower, branch, young fruit pericarp, young fruit pulp, and matured fruit, were collected from the same *P. salicina* tree. Total RNA was extracted from the 6 tissues using E.N.Z.A.^®^ Plant RNA kit (OMEGA, USA).

### Library construction and sequencing

A combination of PacBio single-molecule real-time (SMRT) sequencing, Illumina paired-end sequencing, and Hi-C technology was applied. For PacBio sequencing, SMRT libraries were constructed using the PacBio 20-kb protocol [34]. The Illumina DNA paired-end libraries were constructed with an insert size of 350 bp, and sequencing was performed on the Illumina HiSeq 4000 platform according to the manufacturer's instructions. Reads with adaptors, with >10% unknown bases (N), and with >50% low-quality bases (≤5) were filtered out to obtain clean data for further analysis.

The Hi-C library was prepared using standard procedures. Young leaves of the same *P. salicina* tree were used as starting materials. Nuclear DNA from young leaves was cross-linked *in situ*, extracted, and digested with DpnII restriction endonuclease. The 5′ overhangs of the digested fragments were biotinylated, and the resulting blunt ends were ligated. The cross-links were reversed after ligation, and proteins were removed to release the DNA molecules. The purified DNA was sheared to a mean fragment size of 350 bp and ligated to adaptors, followed by purification through biotin-streptavidin–mediated pull-down. The quality of Hi-C sequencing was evaluated with HiCUP [35].

The RNA-seq libraries for the 6 tissues of *P. salicina* were constructed according to the manufacturer's protocols and were sequenced by Illumina Hiseq 4000 in paired-end 150-bp mode.

### Genome size estimation and *de novo* assembly

Sequencing data from the Illumina library were used to perform a *k*-mer analysis to estimate the genome size of *P. salicina*. Quality-filtered reads were subjected to 17-mer frequency distribution analysis using SOAPdenovo (SOAPdenovo, RRID:SCR_010752) [36].

The *de novo* assembly of the *P. salicina* genome was carried out using the FALCON assembler (FALCON, RRID:SCR_016089) [37], followed by polishing with Quiver [38] and Pilon (Pilon, RRID:SCR_014731) [39]. The PacBio subreads were subsequently processed by a self-correction of errors using FALCON [37] according to the manufacturer's instructions with the following parameters: length_cutoff = 7000, length_cutoff_pr = 4000, max_diff = 100, max_cov = 100. The draft assembly was further polished using Quiver [38]. The “Purge Haplotigs” pipeline was used to remove the redundant sequences caused by genomic heterozygosity [40]. Finally, the Illumina reads were mapped back to the assembly and the remaining errors were corrected by Pilon [39].

Clean Hi-C reads were aligned to the assembled genome with BWA (BWA, RRID:SCR_010910) with default parameters [41]. Only uniquely aligned read pairs with mapping quality >20 were retained for further analysis. Invalid read pairs, including dangling-end and self-cycle, religation, and dumped products, were filtered by HiCUP [35]. The valid interaction pairs were used to cluster, order, and orient the assembly contigs onto pseudochromosomes by LACHESIS (LACHESIS, RRID:SCR_017644) (parameters: CLUSTER_N = 8, CLUSTER_MIN_RE_SITES = 1157, CLUSTER_MAX_LINK_DENSITY = 5, CLUSTER _NONINFORMATIVE_RATIO = 0) [42]. Juicebox [43] was applied to build the interaction matrices and complete the visual correction.

### Genome quality evaluation

To evaluate the coverage of the assembly, the paired-end Illumina short reads were aligned to the assembly using BWA. RNA-seq reads from 6 tissues of *P. salicina* were mapped against our assembly using Hisat with default parameters [44]. The single-nucleotide polymorphisms (SNPs) were counted to evaluate the accuracy of the genome assembly. For CEGMA (CEGMA, RRID:SCR_015055) evaluation, a set of highly reliable conserved protein families that occur in a range of model eukaryotes were built and then the 248 core eukaryotic genes were mapped to the genome [45]. Genome completeness was also assessed using BUSCO (BUSCO, RRID:SCR_015008) analysis, which included a set of 1,440 single-copy orthologous genes [46].

### Repeat annotations

To annotate repeat elements in the *P. salicina* genome, a combined strategy based on homology searching and *de novo* prediction was applied. For homology-based prediction, interspersed repeats were identified using RepeatMasker (RepeatMasker, RRID:SCR_012954) [47] and RepeatProteinMask (RepeatProteinMask, RRID:SCR_012954) [48] to search against the Repbase database [49]. For *de novo* prediction, RepeatScout (RepeatScout, RRID:SCR_014653) [47, 50], RepeatModeler (RepeatModeler (RRID:SCR_015027) [51], and LTR_Finder (LTR_Finder, RRID:SCR_015247) [52, 53] were used to identify *de novo* involved repeats. Tandem repeats were also *de novo* predicted using TRF [54].

Telomere sequences were identified by BLASTN searches of both ends of the pseudochromosomes using 4 tandem repeats of the telomere repeat motif (TTTAGGG) with E-value cut-off of 0.003.

### Gene annotations

A combination of 3 approaches, including homology-based prediction, *de novo* prediction, and transcriptome-based prediction, was used to predict the protein-coding genes within the *P. salicina* genome. For homology-based prediction, the homologous protein sequences of *Prunus persica, Prunus avium, P. mume, Pyrus bretschneideri, Malus domestica, Fragaria vesca*, and *Arabidopsis thaliana* were obtained from the NCBI database and mapped onto the *P. salicina* genome using TblastN (TBLASTN, RRID:SCR_011822) (E-value ≤ 1e−5) [55], and then the matching proteins were aligned to the homologous genome sequences for accurate spliced alignments with GeneWise (GeneWise, RRID:SCR_015054) [56] to define gene models. For *de novo* prediction, Augustus (Augustus, RRID:SCR_008417) [57], GlimmerHMM (GlimmerHMM, RRID:SCR_002654) [58], SNAP (SNAP, RRID:SCR_002127) [59], GeneID (GeneID, RRID:SCR_002473) [60], and Genescan (Genescan, RRID:SCR_012902) [61] were used to predict the coding regions of genes. For transcriptome-based predictions, RNA-seq data from 6 tissues were used for genome annotation, processed by HISAT2 (HISAT2, RRID:SCR_015530) [44] and Stringtie (StringTie, RRID:SCR_016323) [62]. RNA-seq data were also *de novo* assembled with Trinity (Trinity, RRID:SCR_013048) [63]. The assembled sequences were aligned against *P. salicina* genome with PASA (PASA, RRID:SCR_014656) [64], and the effective alignments were assembled to gene structures. Gene models predicted by all of the methods were integrated by EVidenceModeler (EVidenceModeler, RRID:SCR_014659) [64]. To update the gene models, PASA was further used to generate untranslated regions [64].

### Gene functions

The functional annotation of protein-coding genes within the *P. salicina* genome was carried out by aligning protein sequences against the SwissProt [65] and NR databases using BLASTp (with a threshold of E-value ≤ 1e−5). The protein motifs and domains were annotated by searching against InterPro (InterPro, RRID:SCR_006695) [66] and Pfam (Pfam, RRID:SCR_004726) database [67] with InterProScan (InterProScan, RRID:SCR_005829) [68]. Gene Ontology (GO) terms for each gene were retrieved according to the corresponding InterPro entry. KEGG pathways were mapped by the constructed gene set to identify the best match for each gene [69].

### Non-coding RNA annotation

The transfer RNAs (tRNAs) were predicted using the program tRNAscan-SE (tRNAscan-SE, RRID:SCR_010835) [70], and ribosomal RNA (rRNA) genes were annotated using the BLASTN (BLASTN, RRID:SCR_001598) tool with E-value of 1e−5 against rRNA sequences from several relative plant species. MicroRNA and small nuclear RNA were identified by searching against the Rfam (Rfam, RRID:SCR_007891) database [71] with default parameters using the INFERNAL software (INFERNAL, RRID:SCR_011809) [72].

### Gene family construction

OrthoFinder version 2.3.3 (OrthoFinder, RRID:SCR_017118) [73] was used to classify the orthogroups of proteins from *P. salicina* and 16 other sequenced rosids species, including *Prunus armeniaca, P. mume, P. persica, Prunus dulcis, P. avium, Prunus*×*yedoensis, M. domestica, P. bretschneideri, Pyrus communis, Fragaria vesca, Potentilla micrantha, Rosa chinensis, Rosa multiflora, Rubus occidentalis, Morus notabilis*, and *A. thaliana*.

### Phylogenetic tree and divergence time estimation

For phylogenetic tree construction, proteins of single-copy orthogroups (i.e., the orthogroups that contain none or only 1 gene for each species) presented in ≥70% of species were selected and aligned with MAFFT version 6.846b (MAFFT, RRID:SCR_011811) [74]. After determination of the best substitution model for each orthogroup with IQ-TREE version 1.7-beta12 (IQ-TREE, RRID:SCR_017254) [75], the maximum likelihood phylogenetic tree across the 17 plant species was constructed using IQ-TREE with the parameter (-p -bb 1000), setting *A. thaliana* as outgroup.

The divergence time of each node in the phylogenetic tree was estimated with BEAST (BEAST, RRID:SCR_010228) [76]. Two fossil constraints and a secondary calibration node were applied. The fossil *Prunus wutuensis* (age: Early Eocene, minimum age of 55.0 million years ago [Mya]) and the fossil *Rubus acutiformis* (age: Middle Eocene, minimum age of 41.3 Mya) were placed at the stem *Prunus* and *Rubus*, respectively [77]. For the secondary calibration node, the divergence of Rosoideae and Amygdaloideae at 100.7 Mya was dated according to Xiang et al. [77]. The Markov chain Monte Carlo was reported 10,000,000 times with 1,000 steps.

### Gene family expansion and contraction analysis

For gene family expansion and contraction analysis, the ancestral gene content of each cluster at each node was investigated with CAFÉ version 3.1 (CAFÉ, RRID:SCR_005983) [78]; on the basis of the phylogeny and gene numbers per orthogroup in each species, the gene family expansions/contractions at each branch were determined with *P* < 0.001.

### Genome synteny analysis

A Python version of MCScan (MCScan, RRID:SCR_017650) (minspan = 100) [79] was used to analyze the synteny between the *P. salicina* genome and other genomes within *Prunus* following the approaches of Haibao Tang [80].

### Positively selected gene analysis

The ratios of nonsynonymous to synonymous substitutions (Ka/Ks) were calculated using the Codeml program with the free-ratio model as implemented in the PAML (PAML, RRID:SCR_014932) package [81]. The positive selection analysis was performed using the Codeml program with the optimized branch-site model as implemented in the PAML package. The positively selected genes were subjected to GO functional annotation.

### Gene Ontology enrichment analysis

The GO enrichment analysis for the specific groups of genes (e.g., tandem duplication and expanded genes) was performed using the R package “topGO” [82], setting all *P. salicina* genes as background. The lowest-level GO terms under enrichment (*P* < 0.01) were focused, and *P*-value was calculated using a “classic” algorithm with the Fisher test. The lowest-level GO terms were based on the directed acyclic graph of GO, with the parameter “nodeSize = 100.”

### Identification of DUF579 family members

For the identification of the DUF579 family members, the hidden Markov model (HMM) profile corresponding to the DUF579 domain (PF04669) was downloaded from the Pfam database [83] and subsequently exploited for the genome of *P. salicina, P. persica, P. mume, P. armeniaca, P. dulcis*, and *A. thaliana* using HMMER 3.0. The default parameters were used and the cutoff value was set to 0.01.

## Results and Discussion

### Genome sequencing and assembly

We sequenced and assembled the genome of *P. salicina* using a combination of short-read sequencing from Illumina Hiseq, SMRT sequencing from PacBio, and Hi-C technology. For the Illumina sequencing, a total of ∼26.6 Gb (85.4× coverage) short reads was obtained (Supplementary Table S1). A total of ∼53.0 Gb long-sequencing reads were generated by PacBio Sequel platform. After removing adaptors within sequences, ∼52.9 Gb (169.7× coverage) subreads were obtained (Supplementary Table S1). The subreads have a mean length of 13.2 kb (Supplementary Table S2). Roughly 59.1 Gb (189.5× coverage) sequencing data generated from Hi-C library were produced (Supplementary Table S1). The quality of Hi-C sequencing was evaluated with HiCUP [35], and the effect rate was ∼28.10% (Supplementary Table S3).

In the genome assembly process, Illumina sequencing data were used for the genome survey and polishing of preliminary contigs, PacBio long reads were used for contig assembly, and Hi-C reads were used for chromosome-level scaffolding. Based on the total number of *k*-mers (19,341,904,177), the estimated *P. salicina* genome size was calculated to be ∼311.82 Mb (Supplementary Fig. S1). The heterozygous and repeat sequencing ratios were 0.70% and 54.49%, respectively (Supplementary Table S4). The *de novo* genome assembly of *P. salicina*with a total length of 284.2 Mb (Table 1) was yielded. As shown in Fig. 1, the Hi-C–assisted genome assembly was anchored onto the 8 pseudochromosomes with lengths ranging from 23.70 to 54.53 Mb (Supplementary Table S5), which were designated according to the published genetic map of *P. salicina* [84]. Five regions of tandemly repeated telomeric repeat sequences were identified on 3 pseudochromosomes (Supplementary Table S5). The total length of pseudochromosomes accounted for 96.56% of the genome sequences (Fig. 1), with contig N50 of 1.78 Mb and scaffold N50 of 32.32 Mb (Table 1; Supplementary Table S6).

**Table 1:** Summary of genome assembly and annotation for *P. salicina*

Parameter	Value
**Assembly feature**	
Scaffolds	
Total length (bp)	284,209,110
No.	75
N50 (bp)	32,324,625
Contigs	
Total length (bp)	284,189,410
No.	272
N50 (bp)	1,777,944
Mapping rate by reads from short-insert libraries (%)	96.93
CEGs (%)	
Assembled	94.35
Completely assembled	92.34
BUSCOs (%)	
Complete	95.7
Complete and single-copy	86.5
Complete and duplicated	9.2
Fragmented	1.3
Missing	3.0
RNA-Seq evaluation	92.44–95.25
**Genome annotation**	
TEs (%)	48.28
LTR retrotransposons (%)	42.10
No. of predicted protein-coding genes	24,448
No. (%) of genes	
Assigned to pseudochromosomes	24,209 (99.0)
Annotated to public database	23,931 (97.9)
Annotated to GO database	13,484 (55.2)
Duplicated by tandem duplications	2,384 (9.8)

CEG: core eukaryotic gene; LTR: long terminal repeat; TE: transposable element.

### Evaluation of the genome assembly

To assess the genome assembly quality, the Illumina clean data were aligned to the *P. salicina* genome, with the mapping rate of 96.93%. A total of 98.81% assembled genome was covered by the reads, and the mapping coverage with ≥4×, 10×, 20× was 98.48%, 98.06%, and 97.13%, respectively (Table 1; Supplementary Table S7). The RNA-seq reads were mapped against the genome assembly, and the percentage of aligned reads ranged from 92.44% to 95.25% (Table 1; Supplementary Table S8). A total of 3,668 homozygous SNPs were identified, accounting for only 0.0015% of the reference genome (Supplementary Table S9). The low rate of homozygous SNPs suggested that the assembly had a high base accuracy. A total of 234 core eukaryotic genes (CEGs) out of the complete set of 248 CEGs (94.35%) were covered by the assembly, and 229 (92.34%) of these were complete (Table 1; Supplementary Table S10). BUSCO analysis based on the set of single-copy orthologs showed that 95.7% of the expected genes were identified as complete, 1.3% were fragmented, and only 3.0% were missing (Table 1; Supplementary Table S11). These results verified the high quality of the presently generated *P. salicina* genome assembly.

### Genome annotation

The results of the repeat annotations found that 48.28% of the assembly was covered with transposable elements (TEs). Among them, long terminal repeat (LTR) retrotransposons represented the greatest proportion, making up 42.10% of the genome (Table 1; Supplementary Table S12). The TE percentage and density of duplicates resulting from tandem duplications are shown in Fig. 1. Tandem duplicates occurred for 9.8% of the genes (Table 1) and were preferentially enriched in “transferase activity (GO: 0016758 and GO: 0016747)” and “phloem development (GO: 0010088)” (Supplementary Fig. S2). The significant enrichment of the sieve element occlusion genes in phloem development, which are involved in wound sealing of the phloem [85], might be associated with specific requirements during the damage response in *P. salicina*.

For gene annotations, we predicted 24,448 non-redundant protein-coding genes in *P. salicina*. There were 24,209 genes (∼99.0%) that could be assigned to 8 pseudochromosomes (Table 1), and the gene density is shown in Fig. 1. The mean number of exons per gene and mean coding sequence length were 4.97 and 1,157.42, respectively (Table 2). Further gene functional annotation showed that 23,931 (97.9%) protein-coding genes were successfully annotated (Table 1; Supplementary Table S13). For the identification of non-coding RNA (ncRNA) genes, a total of 627 microRNA, 960 tRNA, 273 rRNA, and 2,023 small nuclear RNA in the *P. salicina* genome were predicted (Supplementary Table S14).

**Table 2:** Statistics of predicted protein-coding genes

Gene set		No.	Mean transcript length (bp)	Mean CDS length (bp)	Mean exons per gene	Mean exon length (bp)	Mean intron length (bp)
*De novo* prediction	Augustus	23,592	2,627.71	1,167.83	4.80	243.43	384.45
	GlimmerHMM	39,985	5,450.51	747.07	3.14	238.12	2,200.59
	SNAP	24,882	2,876.50	728.45	4.22	172.73	667.66
	Geneid	33,780	3,829.40	899.99	4.44	202.74	851.78
	Genscan	21,882	8,251.09	1,355.87	6.34	213.98	1,292.13
Homolog prediction	*Pyrus bretschneideri*	20,265	3,119.83	1,356.17	4.74	286.35	472.06
	*Malus domestica*	20,010	2,920.17	1,361.30	4.65	292.56	426.72
	*Prunus mume*	23,064	3,038.66	1,346.19	4.78	281.67	447.84
	*Prunus persica*	28,915	2,296.51	1,099.56	4.06	270.55	390.64
	*Arabidopsis thaliana*	28,284	2,071.73	973.28	3.67	265.51	412.07
	*Fragaria vesca*	22,927	2,994.24	1,380.61	4.59	300.66	449.24
	*Prunus avium*	22,715	3,077.20	1,351.28	4.74	284.86	461.03
RNA-seq	PASA	196,264	3,913.86	1,008.68	5.16	195.60	698.88
	Transcripts	42,450	11,076.28	2,360.92	6.85	344.83	1,490.64
EVM		27,981	2,736.70	1,061.73	4.57	232.52	469.68
PASA-update*		27,594	2,784.15	1,092.82	4.64	235.59	464.83
Final set*		24,448	2,988.45	1,157.42	4.97	233.09	461.72

* Includes untranslated regions. CDS: coding sequence.

### Evolution of the *P. salicina* genome

The genome sequences of the representative sequenced rosid species were collected and subjected to comparative genomic analysis with *P. salicina* to reveal the genome evolution and divergence of *P. salicina*. A total of 15,751 orthogroups containing 23,265 genes were found in *P. salicina*. Moreover, 1,010 genes that were specific to *P. salicina* were identified. A comparison of the predicted proteomes among the 17 species indicated that 9,616, 10,447, 11,098, 13,963, and 15,512 orthogroups were shared between *P. salicina* and Rosids, Rosales, Rosaceae, Amygdaloideae, and *Prunus*, respectively.

**Figure 2:**
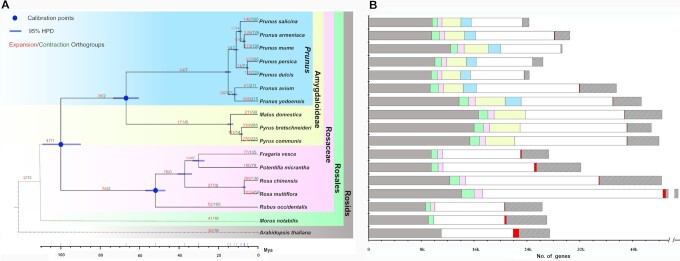
Evolution of *P. salicina* genome and orthogroups. (A) Phylogeny, divergence time, and orthogroup expansions/contractions for 17 rosids species. The tree was constructed by maximum likelihood method using 341 single-copy orthogroups. All nodes have 100% bootstrap support. Divergence time was estimated on a basis of 3 calibration points (blue circles). Blue bar indicates 95% highest posterior density (HPD) for each node. The numbers in red and green indicate the numbers of orthogroups that have expanded and contracted along particular branches, respectively. (B) Comparison of genes among 17 rosids. The grey bars indicate the genes belonging to 9,616 rosids-shared orthogroups in each of 17 rosids. The grey + green bars indicate the genes belonging to 10,447 rosales-shared orthogroups in each of 16 rosales. The grey + green + pink bars indicate the genes belonging to 11,098 Rosaceae-shared orthogroups in each of 15 Rosaceae. The grey + green + pink + yellow bars indicate the genes belonging to 13,963 rosaceae-shared orthogroups in each of ten Amygdaloideae. The grey + green + pink + yellow + blue bars indicate the genes belonging to 15,512 *Prunus*-shared orthogroups in each of 7 *Prunus* species. The red and striped bars indicate the genes in species-specific orthogroups and unassigned genes, respectively. The white bars indicate the remaining genes for each genome.

The phylogenetic analysis confirmed the close relationship among *P. salicina, P. mume*, and *P. armeniaca*. The molecular clock of these plant genomes was also calculated. The data indicated that *P. salicina* diverged from the ancestor of *P. mume* and *P. armeniaca* ∼9.05 Mya, and from the ancestor of *P. persica* and *P. dulcis* 11.12 Mya (Fig. 2).

**Figure 3:**
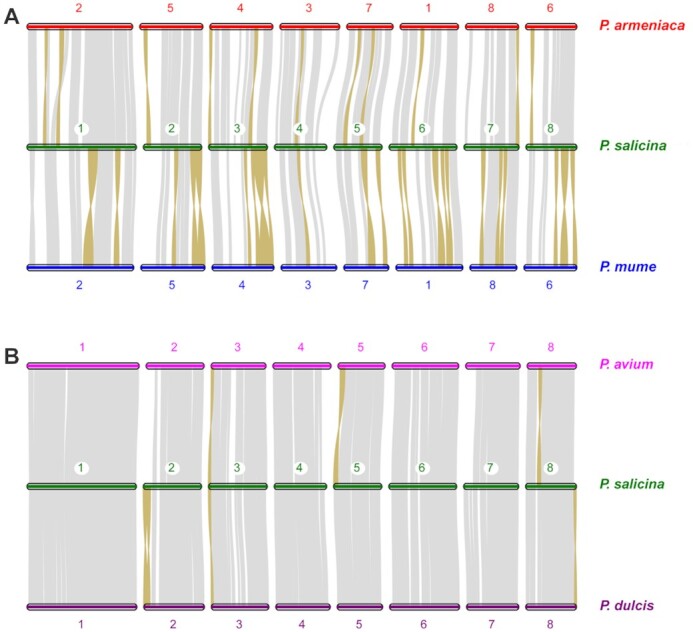
Chromosome-level collinearity patterns (A) between *P. salicina, P. mume*, and *P. armeniaca* and (B) between *P. salicina, P. avium* and *P. dulcis*. The numbers indicate the pseudochromosome order generated from the original genome sequence. The pseudochromosome 2 and 6 in *P. armeniaca* and *P. mume* are reversed. Each grey line represents 1 block. The inverted regions are highlighted with brown color.

We also explored the genome syntenic blocks between *P. salicina* and the other representative *Prunus* species. As shown in Fig. 3, our genome assembly of *P. salicina* exhibited a high level of genome synteny with all the other *Prunus* genomes, especially the genomes of *P. avium* and *P. dulcis*. Significantly fewer inversions were found in *P. salicina* vs *P. avium* and *P. salicina* vs *P. dulcis* than that in *P. salicina* vs *P. mume* and *P. salicina* vs *P. armeniaca*.

### Expansion and contraction of gene families in *P. salicina*

The gene family analysis showed that during the evolution of *P. salicina*, 146 gene families were expanded and 500 gene families were contracted. The functional enrichment on GO of those expanded gene families identified 60 significantly enriched GO terms (*P* < 0.05) (Supplementary Table S15; Supplementary Fig. S3).

**Figure 4:**
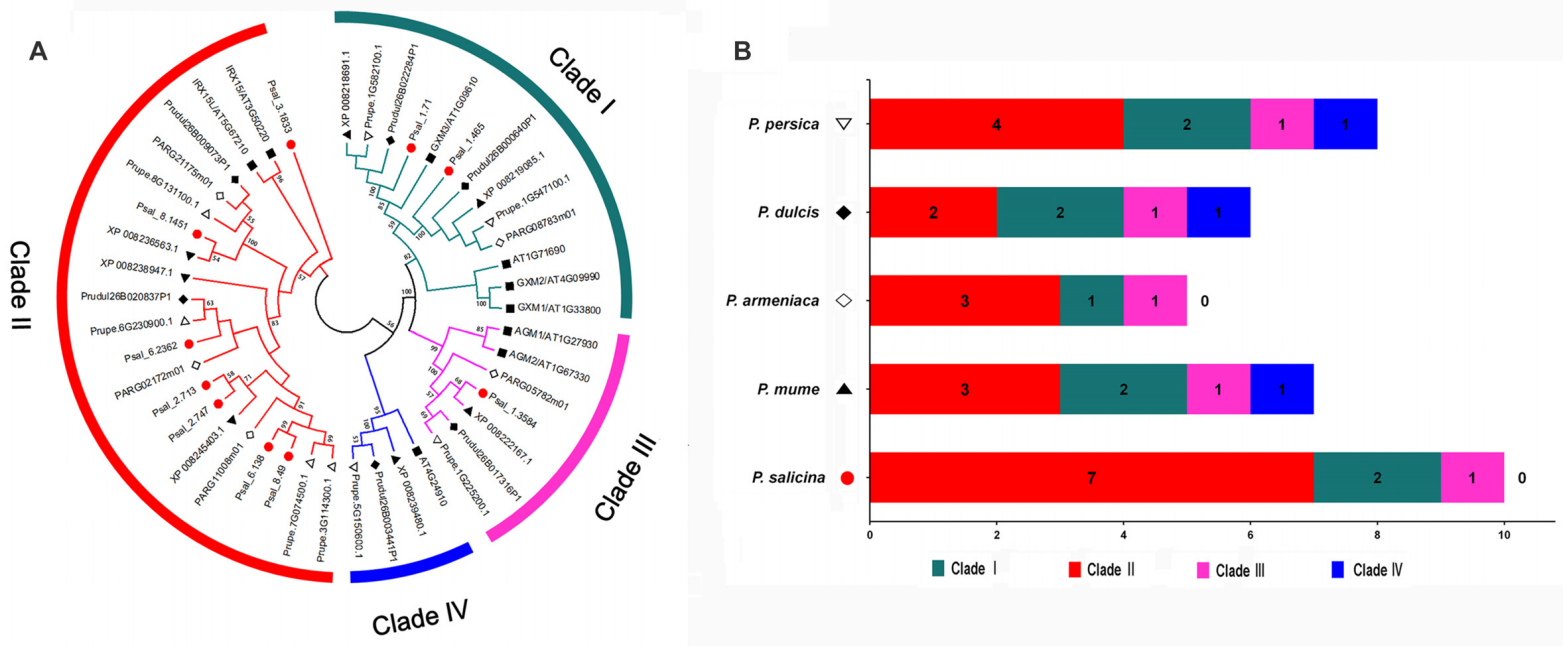
The significant expansion of the DUF579 family members in *P. salicina*. (A) Phylogenetic tree of the DUF579 proteins from *P. salicina* (red cicle), *P. persica* (hollow inverted triangle), *P. mume* (solid triangle), *P. armeniaca* (hollow diamond), *P. dulcis* (solid diamond), and *A. thaliana* (solid square). (B) The summary of the numbers of clade members in DUF579 family.

It is noteworthy that genes from the expanded families were enriched in a series of cell wall–related processes, such as “cell wall polysaccharide metabolic process (GO: 0010383),” “hemicellulose metabolic process (GO: 0010410),” and “regulation of cellular biosynthetic process (GO: 0031326).” Specially, genes in “xylan biosynthetic process (GO: 0045492),” which correspond to the DUF579 family [86], were significantly expanded. Further investigation showed that the major copy differences were found in Clade II, which consisted of orthologs of IRX15/IRX15L [86], with 7 members in *P. salicina* and only 2–4 members in other *Prunus* species (Fig. 4). It has been reported that IRX15 and IRX15L defined a new class of genes involved in xylan biosynthesis [87, 88]. The species-specific expansion of this new subclade might contribute to the relatively high content of xylan-related metabolites (e.g., xylose and xyliot) in plum [10, 11], which provide new insight into the xylan metabolism in plum.

Moreover, the FRS (FAR1-related sequence) gene family, which plays multiple roles in a wide range of cellular processes [89], was also significantly expanded in the phylogeny (GO: 000945), and the family expansion may be related to the genetic and phenotypic diversity in *P. salicina*.

### Positively selected genes in *P. salicina*

The Ka/Ks ratios for all 2,314 single-copy orthologs shared with the sequenced *Prunus* species were calculated. A total of 213 candidate genes in *P. salicina* underwent positive selection (*P* < 0.05). Most of them were enriched in the GO terms involved in “monooxygenase activity (GO: 0004497)” and “enzyme inhibitor activity (GO: 0004857)” (Supplementary Fig. S4). It is noteworthy that the category “monooxygenase activity” was also found in the enriched GO terms for the expanded gene families in *P. salicina*, which might provide valuable candidate genes for further functional investigations.

## Conclusions

To our knowledge, this is the first report of the chromosome-level genome assembly of plums using Illumina and PacBio sequencing platforms with Hi-C technology. The assembly had a total size of 284.2 Mb, and the contig and scaffold N50 reached 1.8 and 32.3 Mb, respectively. A total of 24,448 protein-coding genes were predicted, and 23,931 genes (97.9%) have been annotated. Phylogenetic analysis indicated that *P. salicina* was closely related to *P. mume* and *P. armeniaca*. Expanded gene families in *P. salicina* were significantly enriched in several cell wall–related processes. Remarkably, the *P. salicina*–specific expansion of the xylan biosynthesis–related DUF579 family provided new insight into the xylan metabolism in plums. Given the economic and evolutionary importance of *P. salicina*, the genomic data in this study offer a valuable resource for facilitating plum breeding programs and studying the genetic basis for agronomic and adaptive divergence of plum and *Prunus* species.

## Data Availability

This Whole Genome Shotgun project has been deposited at DDBJ/ENA/GenBank under the accession WERZ00000000. The version described in this article is version WERZ01000000. The raw sequencing data are available through the NCBI SRA via accession Nos. SRR10233497–SRR10233505, via the Project PRJNA574159. The transcriptome data are available through the NCBI SRA (Nos. SRR10235674–SRR10235679). The genome data have also been submitted to Genome Database for Rosaceae (Accession No. tfGDR1044). All annotation tables containing results of an analysis of the draft genome are available at Figshare [90]. Supporting data are also available via the *GigaScience* database GigaDB [91].

## Additional Files

**Supplementary Table S1**. Statistics of *P. salicina* genome sequencing data.

**Supplementary Table S2**. Statistics of characteristics of PacBio long reads.

**Supplementary Table S3**. Statistics of Hi-C sequencing data.

**Supplementary Table S4**. Estimation of the genome size using *k*-mer analysis.

**Supplementary Table S5**. Summary of assembled 8 pseudochromosomes of *P. salicina*.

**Supplementary Table S6**. Summary of the genome assembly of *P. salicina*.

**Supplementary Table S7**. Statistics of mapping ratio in genome.

**Supplementary Table S8**. Summary of the transcriptome and their mapping rate on the genome assembly.

**Supplementary Table S9**. Number and density of SNPs in *P. salicina* genome.

**Supplementary Table S10**. Assessment of CEGMA.

**Supplementary Table S11**. Summary of BUSCO analysis results according to prediction.

**Supplementary Table S12**. Detailed classification of repeat sequences.

**Supplementary Table S13**. Statistics of functional annotation.

**Supplementary Table S14**. Summary of non-coding RNA.

**Supplementary Table S15**. List of the gene ontology terms significantly enriched in the expanded gene families of *P. salicina*.

**Supplementary Figure S1**. 17-mer frequency distribution in *P. salicina* genome.

**Supplementary Figure S2**. Gene ontology enrichment of the tandemly duplicated genes in *P. salicina*.

**Supplementary Figure S3**. Gene ontology enrichment of *P. salicina*–expanded genes.

**Supplementary Figure S4**. Gene ontology enrichment of the positively selected genes in *P. salicina*.

## Abbreviations

BLAST: Basic Local Alignment Search Tool; BEAST: Bayesian Evolutionary Analysis Sampling Trees; bp: base pairs; BUSCO: Benchmarking Universal Single-Copy Orthologs; BWA: Burrows-Wheeler Aligner; CEG: core eukaryotic gene; CEGMA: Core Eukaryotic Genes Mapping Approach; CTAB: cetyltrimethylammonium bromide; EVM: EVidenceModeler; Gb: gigabase pairs; GO: Gene Ontology; Hi-C: high-throughput chromosome conformation capture; HMM: hidden Markov model; kb: kilobase pairs; KEGG: Kyoto Encyclopedia of Genes and Genomes; LTR: long terminal repeat; MAFFT: Multiple Alignment using Fast Fourier Transform; Mb: megabase pairs; Mya: million years ago; NCBI: National Center for Biotechnology Information; PacBio: Pacific Biosciences; PAML: Phylogenetic Analysis by Maximum Likelihood; PASA: Program to Assemble Spliced Alignments; RNA-seq: RNA sequencing; rRNA: ribosomal RNA; SMRT: single-molecule real-time; SNP: single-nucleotide polymorphism; SRA: Sequence Read Archive; TE: transposable element; TRF: Tandem Repeats Finder; tRNA: transfer RNA.

## Competing Interests

The authors declare that they have no competing interests.

## Funding

This work was financially supported by The Industry University Research Collaborative Innovation Major Projects of Guangzhou Science Technology Innovation Commission (201704020021) and Modern Agricultural Industry Technology System of Guangdong Province (2016LM1128).

## Authors’ Contributions

Y.H.H. conceived the study. C.Y.L., C.F., and J.T.W. performed bioinformatics analysis. W.Z.P., J.J.H., and J.J.P. collected the samples and extracted the DNA. C.Y. L. and C. F. wrote the manuscript. All authors read and approved the final manuscript.

## Supplementary Material

giaa130_GIGA-D-20-00195_Original_Submission

giaa130_GIGA-D-20-00195_Revision_1

giaa130_GIGA-D-20-00195_Revision_2

giaa130_Response_to_Reviewer_Comments_Original_Submission

giaa130_Response_to_Reviewer_Comments_Revision_1

giaa130_Reviewer_1_Report_Original_Submission Veronique Decroocq, Ph.D -- 7/20/2020 Reviewed

giaa130_Reviewer_1_Report_Revision_1 Veronique Decroocq, Ph.D -- 9/20/2020 Reviewed

giaa130_Reviewer_1_Report_Revision_2 Veronique Decroocq, Ph.D -- 9/28/2020 Reviewed

giaa130_Reviewer_2_Report_Revision_1 Luca Bianco, Ph.D -- 9/14/2020 Reviewed

giaa130_Reviewer_2_Repor_Original_Submission Luca Bianco, Ph.D -- 7/22/2020 Reviewed

giaa130_Supplemental_Files
