# Supplementary material for: Chromosome-level draft genome of a diploid plum (Prunus salicina)
Source: Gigascience. 2020 Dec 10;9(12):giaa130. doi: 10.1093/gigascience/giaa130 (PMC7727024; doi:10.1093/gigascience/giaa130)
Supplement: giaa130_GIGA-D-20-00195_Revision_1 [file giaa130_giga-d-20-00195_revision_1.pdf]

GigaScience

The chromosome-level draft genome of a diploid plum (Prunus salicina)

--Manuscript Draft--

**Manuscript Number:**	GIGA-D-20-00195R1
**Full Title:**	The chromosome-level draft genome of a diploid plum (Prunus salicina)
**Article Type:**	Data Note
**Funding Information:**	The Industry University Research Collaborative Innovation Major Projects of Guangzhou Science Technology Innovation Commission (201704020021) — Dr Yehua He<br>Guangdong Key Laboratory of Innovation Method and Decision Management System (CN) (2016LM1128) — Dr Yehua He
**Abstract:**	Background: Plums are one of the most economically important Rosaceae fruit crops, and contain dozens of species distributed across the world. Until now, only limited genomic information is available for the genetic studies and breeding programs of plums. Prunus salicina , an important diploid plum species, plays a predominant role in modern commercial plums production. Here we selected P. salicina for whole-genome sequencing and presented a chromosome-level genome assembly through the combination of PacBio sequencing, Illumina sequencing and Hi-C technology.<br>Findings: The assembly had a total size of 284.2 Mb, with contig N50 of 1.8Mb and Scaffold N50 of 32.3 Mb. 96.56% of the assembled sequences were anchored onto eight pseudochromosomes and a total of 24,448 protein-coding genes were identified. Phylogenetic analysis showed that P. salicina had closer relationship with P. mume and P. armeniaca , with P. salicina diverging from their common ancestor approximately 9.05 million years ago (Mya). 146 gene families were expanded during P. salicina evolution, and some cell wall-related GO terms were significantly enriched. It was noteworthy that members in the DUF579 family, a new class involved in xylan biosynthesis, were significantly expanded in P. salicina, which provided new insight into the xylan metabolism in plums .<br>Conclusions: We constructed the first high-quality chromosome-level plum genome using PacBio, Illumina and Hi-C technologies. This work provides a valuable resource for facilitating plum breeding programs and studying the genetic diversity mechanisms of plums and Prunus species.
**Corresponding Author:**	Yehua He<br>Soth China Agricultural University<br>Guangzhou, Guangdong Province CHINA
**Corresponding Author Secondary Information:**	
**Corresponding Author's Institution:**	Soth China Agricultural University
**Corresponding Author's Secondary Institution:**	
**First Author:**	Chaoyang Liu
**First Author Secondary Information:**	
**Order of Authors:**	Chaoyang Liu<br>Chao Feng<br>Weizhuo Peng<br>Jingjing Hao<br>Juntao Wang<br>Jianjun Pan

Powered by Editorial Manager® and ProduXion Manager® from Aries Systems Corporation

	Yehua He
**Order of Authors Secondary Information:**	
**Response to Reviewers:**	Dear Editor and Reviewers,<br><br>We would like to thank you for helpful suggestions on our manuscript entitled "The chromosome-level draft genome of a diploid plum (Prunus salicina)" (GIGA-D-20-00195). Following the comments and suggestions, we rewrote the entire manuscript and re-organized the structure of the article. Our genome data have been submitted to Genome Database for Rosaceae (GDR) and received the accession number tfGDR1044. The reviewer's questions regarding heterozygosity, peach physical map and inter-specific hybrid were answered in detail. We carefully proofread the manuscript and corrected inappropriate words and expressions. We have studied the comments carefully and revised the manuscript according to reviewers' suggestions, and we expected that it would meet the publication requirement of GigaScience. A point by point response to the reviewers' comments and questions and the main corrections in the paper were provided below.<br><br>Reviewer reports:<br><br>--Reviewer #1:<br><br># This manuscript reports high-quality assembly and annotation for one Japanese plum (P. salicina) genome. Phylogenetic analysis has been performed based on the identification of orthologous genes. The genomic data are interesting and should be useful for the community, however, the authors do not put forward any clear research question and respective hypotheses. Therefore, the study will be of limited relevance for an international readership. Most importantly, I identified substantial shortcomings that cannot be alleviated on the basis of the data and analyses presented. The main points raised are summarized below:<br><br>The manuscript is poorly prepared. Material and Methods, Results and Discussion sections are not clearly identified and this does not help to estimate the scope and importance of the results presented. Review and discussion on published results in the similar topics and/or related species appeared insufficient. Material & Methods, Results, mixed with discussion, were not clearly presented. It is fine for results and discussion to be combined, but the results still should be presented first, clearly, then followed by relevant discussion. It also requires a proper Material & Methods section, even presented as supplemental information, but at least clearly identified from the results section. This paper needs substantial improvement of its content organization and clarity to be clear and understandable, before it could be re-submitted as a new manuscript. An alternative would be to present it as a short communication but the decision remains to the editorial board.<br><br>Response: Thanks for your suggestions. We are very sorry for the inconvenience due to the poor content organization. For the preparation of our original manuscript, we download several published papers in 'Data Note' section, and take them as reference to arrange the contents of our manuscript. We mainly focus on how to describe our data and ignore the content structures. According to the suggestions from you and editor, the content organizations are significantly improved, and the Methods and Results sections could be clearly identified in our revised manuscript. We hope the clear content structure could make it more convenient for your review.<br><br># The choice of the methodology for genome assembly is also raising question. Japanese plum is self-incompatible, at least in most accessions, and thus highly heterozygous. It is not clear how the authors disentangled the two expected haplotypes (therefore the two sets of 8 pseudomolecules for P. salicina). By the way, it is not clear why they assembled the accession 'Sanyueli', in particular. What is the level of heterozygosity in 'Sanyueli'.<br><br>Response: Thanks very much for your kindly suggestions.<br>(1) Assembling the highly heterozygous Japanese plum genome have long been challenging as a result of its self-incompatible nature [1]. The short Illumina reads and

even hybrid assembly strategies have always been problematic to de novo assemble any complex plant genome having highly heterozygous sequences. However, the problem has been greatly alleviated with the advent of new sequencing technologies as well as accompanying advances in genome assembly algorithms.

In recent years, the single-molecule, real-time (SMRT) PacBio sequencing and chromosome conformation capture (Hi-C) techniques have been used to make significant advances in improving the assembly of plant genomes at the chromosomal level. The PacBio sequencing can generate long reads which overcomes the restriction of the short reads generated from the Illumina sequencing platform [2]. The Hi-C technology has become available to generate reliable chromosome-scale de novo genome assemblies, and the Hi-C data can also be used to phase genome onto separate haplotypes at chromosomal-scale, since homologous chromosomes occupy distinct territories in nuclei, which could be used to distinguish different haplotypes [3].

Moreover, continuous optimizations for the genome assembly algorithms are helpful for us to disentangle the two expected haplotypes of Japanese plum genome. Just as you mentioned, the haplotype phasing is a key problem in heterozygous genome assemblies. The newer generation of genome assemblers, such as FALCON-Phase, Purge Haplotigs and FALCON-Unzip, are able to separate allelic contigs and have considerably improved the quality of highly heterozygous diploid genome [4]. In our study, the pipeline of 'Purge Haplotigs' [5] was used to remove the redundant sequences caused by genomic heterozygosity.

Based on the integration of PacBio sequencing, Hi-C technology and latest generation of genome assemblers, a series of high quality complex plant genomes have been obtained recently, such as the genome of rubber tree (heterozygosity rate of ~1.6%) [6], cushion willow (~0.71%) [7], Camellia sinensis var. sinensis (~1.22%) [8] and Durian (~1.14%) [9]. In our study, the level of heterozygosity for the Japanese plum 'Sanyueli' was about 0.7% (estimated by k-mer analysis), which was significantly lower than many published complex genomes. Therefore, we think it is not a major problem to assemble the Japanese plum genome and disentangle the two expected haplotypes.

(2)The accession 'Sanyueli' is an early-maturing and high-yielding Japanese plum variety and widely cultivated in South China. Besides the economic importance, 'Sanyueli' also has great value in breeding and scientific research for its lowest chilling requirements among the cultivated Japanese plum varieties [10]. Moreover, the preliminary genome survey results show that the heterozygosity rate (~0.7 %) of 'Sanyueli' is not very high. Therefore, 'Sanyueli' is selected for the subsequent genome sequencing and assembly in our study.

Authors used the peach physical map and genome assembly to align the metascaffolds onto 8 pseudo-molecules, corresponding to the eight haploid Prunus chromosomes. How did the authors handle the genomic re-arrangements (translocation, inversions, deletions) between peach and plum? Why didn't they use Japanese plum genetic maps which were previously published?

Response: Thank you very much for your kindly suggestions.
(1) We think there might be some misunderstandings，the peach physical map was not used in the genome assembly process in our study. The chromosome-level de novo genome assembly of Prunus salicina was generated using an integrated strategy that combined PacBio sequencing, Illumina sequencing and Hi-C technology. We used Hi-C to cluster and order contigs of this draft genome assembly into 8 pseudo-molecules, which cover ~96.56% of the total contig length. The genomic data from peach were only used as references in the gene annotation, orthogroup identification and phylogenetic analysis.

(2) Since the peach physical map and genome assembly were not used to align the scaffolds in our study, the genomic re-arrangements between peach and plum were not considered in the genome assembly process. For the Hi-C assisted assembly, we applied LACHESIS to cluster, order, and orient the assembly contigs onto pseudo-molecules.

Powered by Editorial Manager® and ProduXion Manager® from Aries Systems Corporation

(3) Genetic maps are useful tools for guiding scaffold anchoring into pseudo-chromosome assembly [11]. Up to now, only a few genetic linkage maps of Japanese plums have been reported [12-15], and there are still several problems in using them for the assisted genome assembly: ① Most of the parents are not local varieties of Japanese plums; ②The marker numbers and chromosome coverage are limited, and several large gaps are found; ③The original data for most of the genetic maps are not available.

Moreover, the mapping algorithms used to build genetic maps can sometimes place markers at incorrect locations, which could lead to errors in the genome assembly [16]. The Hi-C technology employed in our study is a novel strategy combining capture of chromatin interaction within the nucleus and next-generation sequencing. Hi-C data can effectively identify linkage between contigs or scaffolds, allowing contigs being linked to nearly whole chromosome-scale [4]. This method has been widely used in many species and dramatically improved genome assemblies. For example, Jibran et al. [17] demonstrated that Hi-C analysis had vastly improved the black raspberry genome assembly, yielding a N50 contig size for the Hi-C guided assembly of 31,759,000 bp versus the N50 scaffold size of 48,488 bp for the previously genetic maps-assisted assembled genome of VanBuren et al. [18].

(4) Overall, compared to the published relatively low-density Japanese plum genetic maps, we think that the Hi-C technology has more advantages in the genome assembly. It is certain that the available high-density Japanese plum genetic maps could be used as an important supplement for the improvement of our genome assembly in the future.

P. salicina is inter-fertile with many other Prunus species, P. mume and P. armeniaca included, especially in China. This has been profoundly documented (see Zhang et al, 2018. DOI: 10.1038/s41467-018-04093-z). How did the authors check the fact that cv. 'Sanyueli' is pure Japanese plum and not an inter-specific hybrid?

Response: Thank you very much for your kindly suggestions.
(1) According to the paper you mentioned, there also might be introgression events in Japanese plum cultivars from Prunus species. The interspecific cross-compatibility is found among the diploid plum and non-plum species within the subgenus Prunophora [19]. Moreover, the diploid plums can also be hybridised with species from the subgenera Amygdalus (peach and almond) and Cerasus (cherry) but with less fertility [20]. Many interspecific hybrids have been reported and widely cultivated. For example, Prunus simonii might be a type of natural hybridization between P. salicina and P. armeniaca [21]; 'Santa Rosa' is a complex hybrid containing a mixture of P. salicina, P. saimonii, and P. Americana [22].

(2) 'Sanyueli' is a traditional landraces of Japanese plum and widely cultivated in South China, especially in Guangdong Province. 'Sanyueli' has long cultivation history and has been recorded in local gazetteers of Nanhua County in 1843 [23].

(3) Japanese plum originates in China, has a long cultivation history and wide geographical distribution ranging from the southern to the northern areas of the country. 'Sanyueli' is a low-chilling requirement and cold-sensitive Japanese plum variety, mainly distributed in the south of Japanese plum cultivation regions in China [10, 24].

According to the most widely accepted classification [25], Prunophora subgenus could be subdivided into the sections Euprunus (plum species native to Europe and Asia), Prunocerasus (plum species native to North America) and Armeniaca (apricot species). Among the species of Euprunus and Prunocerasus sections, only Japanese plum is widely found in South China region, according to the germplasm resources investigation [10]. Other plum species are not well adapted to the climate in South China, because the winter temperatures could not meet their chilling requirements for normal flowering in most years. The distribution characteristics of plums show that the natural outcrossing between 'Sanyueli' and other plum species in recent years might be considered as rare events.

Powered by Editorial Manager® and ProduXion Manager® from Aries Systems Corporation

Among the species in Section Armeniaca, only Prunus mume is also widely found in South China, which is overlapped with the distribution of 'Sanyueli'. However, the differences in flowering time might reduce the possibility of natural outcrossing. As far as we know, there are no reports about the natural hybrids between Prunus mume and Prunus salicina. Boonprakob at al. [26] found that the P. mume produce semi-fertile hybrids in crosses with plum species. The interspecific hybrids between P. mume cv. Baigo and P. salicina cv. Sordum were created with manual hybridization by Hakoda et al. [27], and the hybrids can be easily distinguished with their parents according to the morphological characteristics like flower size and leaf shape.

(4) Overall, the above analyses indicate that the cv. 'Sanyueli' is most likely not from the recent interspecific hybridization. We think it could be a suitable candidate material for the Japanese plum genome sequencing. However, we could not rule out the possibility of the introgression from other germplasms like P. mume during the long-term cultivation and domestication of 'Sanyueli'. In the future work, we will perform the whole-genome re-sequencing project for various germplams within Prunophora subgenus. We think the project will help us to better understand the genetic background of 'Sanyueli' and other varieties of Japanese plum.

Given those issues, the analyses appear rudimentary/descriptive and biased, the main conclusions not reliable enough and the previous studies on diversity and genetic studies in Japanese plums not taken into account.

Response: We agree that the analyses in our study maybe not comprehensive enough and the main conclusions need further experimental verification. However, our paper is submitted as a Data Note, which aims to incentivize and more rapidly release data before subsequent detailed analysis has been carried out, so we mainly focuses on presenting the genome data in our manuscript. We have actually noticed the previous studies on diversity and genetic studies in Japanese plum, and carefully selected cv. 'Sanyueli' for genome sequencing. We think that the completion of our high-quality Japanese plum genome will help to measure and characterize the genetic diversity and determine how this diversity relates to the tremendous phenotypic diversity among plum cultivars.

This situation is aggravated by the fact that in many instances, writing is not clear and terminology inappropriate, with many awkward or incorrect sentences (for ex. In the abstract, what does 'hold the center of the Prunus' mean or what is a 'typical' diploid plum species for the authors?). Attention should be given to using correct terms. A substantial English proofreading is required.

Response: Thank you very much for your kindly suggestions. We are sorry for the unclear writing and inappropriate terminology in our original manuscript. We reorganize the article structures and carefully modify the incorrect sentences that you pointed out. The substantial English proofreading is implemented, and the inappropriate words and expressions are corrected in revised manuscript.

Reviewer #2:

The authors report the first chromosome-level genome assembly of plum (P. salicina), which is an economically important fruit crop and therefore provide a useful resource for the research community of this fruit tree. They also provided a phylogenetic analysis with P. nume and P. armenica and studied gene family expansion in P. salicina evolution investigating in particular xylan metabolism which might have an impact on fruit quality.

I believe that the paper is well written and provides a useful resource for the community therefore I would welcome its publication once a few, mostly minor, issues are addressed.

I have seen that the data is/will be available on public repositories but I did not see the assembled sequences and the usual services like BLAST that would make the

Powered by Editorial Manager® and ProduXion Manager® from Aries Systems Corporation

genome truly available for the community. I am not sure whether authors intend to publish this data on their own web-server alongside GigaDB, but I would also recommend to submit sequences/gene predictions to specialized databases like the Genome Database for Rosaceae (GDR) which will make this data easily available for the rosaceae community.

Response: According to your suggestion, we have submitted our genome data to GDR and received the accession number tfGDR1044. The genome data will be available through the link https://www.rosaceae.org/publication_datasets. (Line 435)

Detailed comments

line 31: "Plums are the economically important" I believe should be "Plums are one of the most economically important... and are produced"
Response: We have corrected it according to your suggestion. (Line 31)

line 64: originate should be originates
Response: We corrected accordingly. (Line 63)

line 88: some references here are missing like Daccord et.al, 2017 for the apple GDDH13 genome and Linsmith et al, 2019 for European Pear. The published genomes of Prunus avium, Prunus armenica and Prunus dulcis are also ignored here. I am not an expert in Prunus, but perhaps authors should also consider providing a collinearity analysis with avium and dulcis.
Response: Thanks very much for your kindly suggestions. The references you mentioned have been added in the revised manuscript (Apple GDDH13, Ref 15; European Pear, Ref 17; Sweet cherry, Ref 27; Apricot, Ref 29; Almond, Ref 24)(Line 88-89).According to your suggestion, the collinearity analysis between P. salicina, P. avium and P. dulcis was performed in revised manuscript (Figure 3B, Line 375-379).

line 105: conversation should be conservation
Response: We corrected accordingly. (Line 108)

line 119: I guess that by "with unknown bases (N) than 10%" authors mean "with more than 10% unknown bases (N)", and with more than 50% low quality bases... Please rephrase.
Response: We rephrase the sentence according to your suggestion. (Line 124)

line 145: "were used to estimate the genomic information" I would rephrase this to say that they were used to perform a kmer analysis to estimate the genome size.
Response: Thanks very much for your kindly suggestions. We corrected accordingly. (Line 141)

lines 156-158: this is what FALCON does, so in my opinion there is no need to repeat this here.
Response: We corrected accordingly. (Line 146)

line 189: I would remove approaches.
Response: We corrected accordingly. (Line 174)

line 194: In table 1 it would be interesting to have more information on CEGMA and BUSCO like the % of duplicated genes vs unique etc. which are in the supplementary material
Response: According to your suggestions, more detailed information about CEGMA and BUSCO were added in Table 1.

line 195: It would be interesting to see how many telomeric sequences are recovered at each end of the assembled chromosomes to show how complete they are. I believe this could be a nice addition to this paragraph.
Response: Thanks very much for your kindly suggestions. According to your suggestions, the telomere sequences were identified by BLASTN searches using tandem repeats of the telomere repeat motif (TTTAGGG), and the results were exhibited in Table S5.

#line 211: remove "of"
Response: We corrected accordingly. (Line 342)

#line 213-214: any comment on why transferase activity and phloem development were enriched?
Response: Thanks very much for your kindly suggestions. The possible causes for the significant enrichment of sieve element occlusion genes in ‘phloem development’ were discussed in revised manuscript. (Line 348-350)

#line 221-222: maybe authors should have added the protein sequences from Pyrus Communis as well. In the gene family identification paragraph Pyrus communis is actually mentioned, therefore this might just be an oversight here.
Response: Thanks very much for your kindly suggestions. Sequences from Prunus salicina and other 16 sequenced rosids species, including Pyrus Communis, were actually used in the gene family identification (Line 240). However, only 7 species (Prunus persica, Prunus avium, Prunus mume, Pyrus bretschneideri, Malus domestica, Fragaria vesca and Arabidopsis thaliana) were selected in the homology-based gene prediction (Line 198-199). The Pyrus Communis was not included because the Pyrus bretschneideri was selected as the representative of pear.

#line 224: SwisssProt should be SwissProt
Response: We corrected accordingly. (Line 220)

#Lines 245-247: It is not clear to me if authors used only Interpro results to annotate the plum proteins with the Gene Ontology? In this case, why did they also perform the BLAST search against NR and SwissProt? Otherwise, how did they use the BLAST results to retrieve the GO terms? Please explain.
Response: We only used the Interpro results to annotate the Japanese plum proteins with the Gene Ontology (GO). The GO IDs for each gene were assigned according to the corresponding InterPro entry. The InterPro database, which includes 14 member databses, integrates diverse information about protein families, domains and functional sites [28]. The InterPro databases group one or more related member databases signatures, and provides additional overarching functional annotations, including GO terms wherever possible.The BLAST search against NR and SwissProt databases were also performed in our study, because they were not integrated into the InterPro databases and had different focuses and distinctive signatures. The NR dataset include the non-redundant protein sequences from GenPept, SwissProt, PIR, PDF, PDB, and NCBI Refseq, and the annotations might be more comprehensive. SwissProt is a curated protein sequence database [29], which might be able to provide the high quality annotation.

#Figure 2: The quality of the figure I saw is quite low and it is difficult to read the names. This might be due to the pdf version I have seen, but please double-check
Response: We checked the quality of Figure 2 again, and found that the low figure quality was due to the PDF version that you have seen. The original figure in TIFF format could be downloaded through the link “Click here to access/download” at the top right corner of the PDF pages.

#Figure 3: P. armeniaeca should be P. armenica
Response: According to your suggestion, we corrected the scientific name of apricot (in Figure 3) to P. armeniaca.

Reference
1.Guerra M and Rodrigo J. Japanese plum pollination: A review. Sci Hortic 2015; 197:674-686.
2.Wei S, Yang Y and Yin T. The chromosome-scale assembly of the willow genome provides insight into Salicaceae genome evolution. Hortic Res 2020; 7(1):1-12.
3.Korbel JO and Lee C. Genome assembly and haplotyping with Hi-C. Nat Biotechnol 2013; 31 (12):1099-1101.
4.Zhang X, Wu R, Wang Y, Yu J and Tang H. Unzipping haplotypes in diploid and polyploid genomes. Comput Struct Biotechnol J 2020; 18:66-72.
5.Roach MJ, Schmidt SA and Borneman AR. Purge Haplotigs: allelic contig reassignment for third-gen diploid genome assemblies. BMC Bioinformatics 2018; 19

(1):460.
6.Liu J, Shi C, Shi CC, Li W, Zhang QJ, Zhang Y, et al. The Chromosome-Based Rubber Tree Genome Provides New Insights into Spurge Genome Evolution and Rubber Biosynthesis. Mol Plant 2020; 13 (2):336-350.
7.Chen JH, Huang Y, Brachi B, Yun QZ, Zhang W, Lu W, et al. Genome-wide analysis of Cushion willow provides insights into alpine plant divergence in a biodiversity hotspot. Nat Commun 2019; 10 (1):5230.
8.Zhang Q-J, Li W, Li K, Nan H, Shi C, Zhang Y, et al. SMRT sequencing yields the chromosome-scale reference genome of tea tree, Camellia sinensis var. sinensis. BioRxiv 2020.
9.Teh BT, Lim K, Yong CH, Ng CCY, Rao SR, Rajasegaran V, et al. The draft genome of tropical fruit durian (Durio zibethinus). Nat Genet 2017; 49 (11):1633-1641.
10.He Yehua, Feng Junting, Guo Cuihong, Han Jingzhong, Xie Zhiliang and Yu Xiaolin. Study on plum germplasm of Guangdong (In Chinese). Acta Horticulturae Sinica 2014; 41(S):2610.
11.Xue H, Wang S, Yao JL, Deng CH, Wang L, Su Y, et al. Chromosome level high-density integrated genetic maps improve the Pyrus bretschneideri 'DangshanSuli' v1.0 genome. BMC Genomics 2018; 19 (1):833.
12.Vieira EA, Nodari, RO, de Mesquita Dantas A C, Ducroquet JPHJ, Dalbó M, & Borges, CV. Genetic mapping of Japanese plum. Crop Breed Appl Biot 2005; 5(1):29-37.
13.Salazar JA, Pacheco I, Shinya P, Zapata P, Silva C, Aradhya M, et al. Genotyping by Sequencing for SNP-Based Linkage Analysis and Identification of QTLs Linked to Fruit Quality Traits in Japanese Plum (Prunus salicina Lindl.). Front Plant Sci 2017; 8:476.
14.Carrasco B, Gonzalez M, Gebauer M, Garcia-Gonzalez R, Maldonado J and Silva H. Construction of a highly saturated linkage map in Japanese plum (Prunus salicina L.) using GBS for SNP marker calling. PLoS One 2018; 13 (12):e0208032.
15.Zhang Q-p, Wei X, Liu N, Zhang Y-p, Xu M, Zhang Y-j, et al. Construction of an SNP-based high-density genetic map for Japanese plum in a Chinese population using specific length fragment sequencing. Tree Genetics & Genomes 2020; 16(1):1-10.
16.Tang H, Zhang X, Miao C, Zhang J, Ming R, Schnable JC, et al. ALLMAPS: robust scaffold ordering based on multiple maps. Genome Biol 2015; 16 (1):3.
17.Jibran R, Dzierzon H, Bassil N, Bushakra JM, Edger PP, Sullivan S, et al. Chromosome-scale scaffolding of the black raspberry (Rubus occidentalis L.) genome based on chromatin interaction data. Hortic Res 2018; 5(1): 1-11.
18.VanBuren R, Bryant D, Bushakra JM, Vining KJ, Edger PP, Rowley ER, et al. The genome of black raspberry (Rubus occidentalis). Plant J 2016; 87 (6):535-547.
19.Okie W and Weinberger J. Fruit breeding, volume I: Tree and tropical fruits. John Wiley & Sons, Inc., New York, 1996.
20.Topp BL, Russell DM, Neumüller M, Dalbó MA and Liu W. Plum. In: Maria Luisa Badenes DHB, editor. Fruit Breeding. Springer; 2012.p.571-621.
21.Faust M and Surányi D. Origin and dissemination of plums. Hort Rev. 1999; 23: 179-231.
22.Karp D. Luther Burbank's plums. HortScience 2015; 50(2):189-194.
23.Zhang Jiayan and Zhou En. Records of Chinese fruit-Journal of plum (In Chinese). Beijing: China Forestry Publishing House, 1998. p.17-23.
24.Liu Shuo, Xu Ming, Zhang Yuping, Zhang Yujun, Ma Xiaoxue, Zhang Qiuping, Liu Ning, Liu Weisheng. Retrospect, problematical issues and the prospect of plum breeding in China (In Chinese). Journal of Fruit Science 2018; 35 (2):231-245.
25.Rehder A. Manual of cultivated trees and shrubs. 1949.
26.Boonprakob U and Byrne D. Mume, a possible source of genes in apricot breeding. Fruit Varieties J 1990; 44 (3):108-113.
27.Hakoda N, Toyoda R, Tabuchi T, Ogiwara I, Ishikawa S and Shimura I. Morphological characteristics of the interspecific hybrids between Japanese apricot (Prunus mume) and Plum (P. salicina) (In Japanese). J JPN SOC HORTIC SCI 1998; 67 (5):708-714.
28.Finn RD, Attwood TK, Babbitt PC, Bateman A, Bork P, Bridge AJ, et al. InterPro in 2017-beyond protein family and domain annotations. Nucleic Acids Res 2017; 45 (D1): D190-D199.
29.Gasteiger E, Jung E and Bairoch AM. SWISS-PROT: connecting biomolecular knowledge via a protein database. Curr Issues Mol Biol 2001; 3 (3):47-55.

Additional Information:

Powered by Editorial Manager® and ProduXion Manager® from Aries Systems Corporation

Question	Response
Are you submitting this manuscript to a special series or article collection?	No
**Experimental design and statistics**<br><br>Full details of the experimental design and statistical methods used should be given in the Methods section, as detailed in our Minimum Standards Reporting Checklist. Information essential to interpreting the data presented should be made available in the figure legends.<br><br>Have you included all the information requested in your manuscript?	Yes
**Resources**<br><br>A description of all resources used, including antibodies, cell lines, animals and software tools, with enough information to allow them to be uniquely identified, should be included in the Methods section. Authors are strongly encouraged to cite Research Resource Identifiers (RRIDs) for antibodies, model organisms and tools, where possible.<br><br>Have you included the information requested as detailed in our Minimum Standards Reporting Checklist?	Yes
**Availability of data and materials**<br><br>All datasets and code on which the conclusions of the paper rely must be either included in your submission or deposited in publicly available repositories (where available and ethically appropriate), referencing such data using a unique identifier in the references and in the "Availability of Data and Materials" section of your manuscript.	Yes

Have you have met the above requirement as detailed in our Minimum Standards Reporting Checklist?	

Manuscript

Click here to access/download;Manuscript;Revised manuscript of GIGA-D-20-00195.pdf

The chromosome-level draft genome of a diploid plum (*Prunus salicina*)

Chaoyang Liu[1, 3*], Chao Feng[2,*], Weizhuo Peng[1,3], Jingjing Hao[1,3], Juntao Wang[1,3] Jianjun Pan[4], Yehua He[1,3]

[1] Key Laboratory of Biology and Germplasm Enhancement of Horticultural Crops in South China, Ministry of Agriculture, South China Agricultural University, Guangzhou 510642, China

[2] Key Laboratory of Plant Resources Conservation and Sustainable Utilization, South China Botanical Garden, Chinese Academy of Sciences, Guangzhou 510650, China

[3] Maoming Branch, Guangdong Laboratory for Lingnan Modern Agriculture, Maoming 525000, China

[4] Agricultural Technology Extension Center of Conghua District, Guangzhou. Guangzhou 510900, Guangdong Province, China

*Equal contribution

Corresponding author: Yehua He (email: heyehua@hotmail.com)

Abstract

Background: Plums are one of the most economically important Rosaceae fruit crops, and contain dozens of species distributed across the world. Until now, only limited genomic information is available for the genetic studies and breeding programs of plums. *Prunus salicina*, an important diploid plum species, plays a predominant role in modern commercial plums production. Here we selected *P. salicina* for whole-genome sequencing and presented a chromosome-level genome assembly through the combination of PacBio sequencing, Illumina sequencing and Hi-C technology. **Findings:** The assembly had a total size of 284.2 Mb, with contig N50 of 1.8Mb and scaffold N50 of 32.3Mb. 96.56% of the assembled sequences were anchored onto eight pseudochromosomes and a total of 24,448 protein-coding genes were identified. Phylogenetic analysis showed that *P. salicina* had closer relationship with *P. mume* and *P. armeniaca*, with *P. salicina* diverging from their common ancestor approximately 9.05 million years ago (Mya). 146 gene families were expanded during *P. salicina* evolution, and some cell wall-related GO terms were significantly enriched. It was noteworthy that members in the DUF579 family, a new class involved in xylan biosynthesis, were significantly expanded in *P. salicina,* which provided new insight into the xylan metabolism in plums. **Conclusions:** We constructed the first high-quality chromosome-level plum genome using PacBio, Illumina and Hi-C technologies. This work provides a valuable resource for facilitating plum breeding programs and studying the genetic diversity mechanisms of plums and *Prunus* species.

Background

Plums are one of the most economically important Rosaceae fruit crops and are produced throughout the world. About 12.6 million tons of plums (include sloes) are produced per year (FAOSTAT 2018, http://faostat.fao.org/), and the fruits are widely used for fresh consumption and processing like canning and beverages [1].There are 19-40 species of plums distributed across Asia, Europe and America. Plums have great diversity and are considered as a link between the major subgenera in the genus *Prunus* [2].

Prunus salicina, commonly called the Japanese plum or Chinese plum, is an important diploid (2x=2n=16) plum species that predominates in the modern commercial production of plums (Fig. 1). *P. salicina* originates in China and its fruits are mostly used for fresh consumption for their characteristic taste [3]. Cultivars of *P. salicina* have wide variability in phenology, fruit size and shape, flavour, firmness, aroma, texture, phenolic composition, antioxidant activity and both skin and pulp color [4].

However, the genetic and genomic information for *P. salicina* as well as most plum species were scarce [5]. The availability of a fully sequenced and annotated genome will help to measure and characterize the genetic diversity and determine how this diversity relates to the tremendous phenotypic diversity among plum cultivars. The genomic information is essential to support many of the studies involved in fundamental questions about plums biology and genetics. Moreover, genome-based tools could be developed to improve breeding works of plums, which were usually hindered by the high degree of heterozygosity, self-incompatible and long juvenile stage [2, 5, 6].

The fruit firmness, one of the most important indices of plum quality, is closely associated with cell wall compositions [2]. Xylan is a major component of secondary cell walls [7], and the xylan metabolism is involved in various aspects of plant growth and development like fruit ripening and softening [8]. According to previous studies, the plum species presented more xylose (the main component of xylan) compared to

other *Prunus* species, and plums were regarded as one of the richest natural sources of xyliot [9, 10]. The relatively high levels of xylan-related metabolites may be associated with the distinct mechanisms of the xylan metabolism in plums, and the available plum genomic information will be helpful to better understand the mechanism at molecular level.

Genome resources are already available for a number of Rosaceae fruit crops [11], including apple [12-15], peach [16], pear [17-20], strawberry [21, 22], almond [23, 24], black raspberry [25], sweet cherry [26, 27], apricot [28, 29], loquat [30] and *Prunus mume* [31]. However, whole-genome sequencing and chromosome-level assembly for plums have not been reported until now. In this study, *P. salicina* was selected for the whole-genome sequencing as a genomic reference. A high-quality chromosome-level *de novo* genome assembly of *P. salicina* was generated using an integrated strategy that combines PacBio sequencing, Illumina sequencing and Hi-C technology. The assembly has a total size of 284.2 Mb with contig N50 of 1.8Mb and scaffold N50 of 32.3 Mb, and vast majority (96.56%) of the assembled sequence was anchored onto eight pseudochromosomes. The availability of the high-quality chromosome-scale genome sequences not only provides fundamental knowledge regarding plum biology but also presents a valuable resource for genetic diversity analysis and breeding programs of plums and other *Prunus* crops.

Methods

Sample collection

The *Prunus salicina* Lindl. cv. ‘Sanyueli’, a Japanese plum landrace originating from Southern China, was selected for genome sequencing and assembly. ‘Sanyueli’ has a cultivation history of more than 200 years and many distinctive characteristics, including early-maturation, high-yield and low chilling requirements. The samples of the ‘Sanyueli’ were kept at the Horticultural Germplasm Conservation Center of South China Agricultural University (SCAU) for breeding and research in Guangzhou, Guangdong Province, China (113°22'4" N, 23°9'5" E). Total genomic DNA was

extracted from fresh young leaves of 5-year-old *P. salicina* tree using the CTAB method [32]. Samples from a total of six tissues, including leaf, flower, branch, young fruit pericarp, young fruit pulp and matured fruit, were collected from the same *P. salicina* tree. Total RNA was extracted from the six tissues using E.N.Z.A.® Plant RNA kit (OMEGA).

Library construction and sequencing

A combination of PacBio single-molecule real-time (SMRT) sequencing, Illumina's paired-end sequencing and Hi-C technology was applied. For PacBio sequencing, SMRT libraries were constructed using the PacBio 20-kb protocol (https://www.pacb.com/). The Illumina DNA paired-end libraries were constructed with an insert size of 350 bp, and sequencing was performed on the Illumina HiSeq 4000 platform according to the manufacturer's instructions. Reads with adaptors, with more than 10% unknown bases (N) and with more than 50% low-quality bases ($\leqslant$ 5) were filtered out to gain the clean data for further analysis.

The Hi-C library was prepared using the standard procedures. The young leaves of the same *P. salicina* tree were used as starting materials. Nuclear DNA from young leaves was cross-linked in situ, extracted, and digested with DpnII restriction endonuclease. The 5' overhangs of the digested fragments were biotinylated, and the resulting blunt ends were ligated. The cross-links were reversed after ligation, proteins were removed to release the DNA molecules. The purified DNA was sheared to a mean fragment size of 350 bp and ligated to adaptors, followed by purification through biotin-streptavidin-mediated pull down. The quality of Hi-C sequencing was evaluated with HiCUP [33].

The RNA-seq libraries for the six tissues of *P. salicina* were constructed according to the manufacturer's protocols, and were sequenced by Illumina Hiseq 4000 in paired-end 150bp mode.

Genome size estimation and *de novo* assembly

Sequencing data from the Illumina library were used to perform a k-mer analysis to

estimate the genome size of *P. salicina*. Quality-filtered reads were subjected to 17-mer frequency distribution analysis using SOAPdenovo (SOAPdenovo, RRID: SCR_010752) [34].

The *de novo* assembly of the *P. salicina* genome was carried out using the FALCON assembler (FALCON, RRID: SCR_016089) [35], followed by the polishing with Quiver [36] and Pilon (Pilon, RRID: SCR_014731) [37]. The PacBio subreads were subsequently processed by a self-correction of errors using FALCON [35] according to the manufacturer's instructions with the following parameters: length_cutoff =7,000, length_cutoff_pr = 4,000, max_diff = 100, max_cov = 100. The draft assembly was further polished using Quiver [36]. The pipeline of 'Purge Haplotigs' was used to remove the redundant sequences caused by genomic heterozygosity [38]. Finally, the Illumina reads were mapped back to the assembly and the remaining errors were corrected by Pilon [37].

Clean Hi-C reads were aligned to the assembled genome with BWA aligner (BWA, RRID: SCR 010910) with default parameters [39]. Only uniquely aligned read pairs whose mapping quality more than 20 were remained for further analysis. Invalid read pairs, including dangling-end and self-cycle, relegation, and dumped products, were filtered by HiCUP [33]. The valid interaction pairs were used to cluster, order, and orient the assembly contigs onto pseudochromosomes by LACHESIS (LACHESIS, RRID:SCR_017644; parameters: CLUSTER_N = 8, CLUSTER_MIN_RE_SITES =1157, CLUSTER_MAX_LINK_DENSITY = 5, CLUSTER _NONINFORMATIVE_RATIO = 0) [40]. The Juicebox [41] was applied to build the interaction matrices and complete the visual correction.

Genome quality evaluation

To evaluate the coverage of the assembly, the paired-end Illumina short reads were aligned to the assembly using BWA. RNA-seq reads from six tissues of *P. sacilina* were mapped against our assembly using Hisat with default parameters [42]. The SNPs were counted to evaluate the accuracy of the genome assembly. For CEGMA (Core Eukaryotic Genes Mapping Approach; CEGMA, RRID: SCR_015055)

evaluation, a set of highly reliable conserved protein families that occur in a range of model eukaryotes were build and then the 248 core eukaryotic genes were mapped to the genome [43]. Genome completeness was also accessed using BUSCO (Benchmarking Universal Single-Copy Orthologs; RRID: SCR_015008) analysis which included a set of 1440 single-copy orthologous genes [44].

Repeat annotations

To annotate repeat elements in the *P. salicina* genome, a combined strategy based on homology searching and *de novo* prediction was applied. For homology-based prediction, interspersed repeats were identified using RepeatMasker (http://www.repeatmasker.org) (RepeatMasker, RRID: SCR_012954) and RepeatProteinMask (RepeatProteinMask, RRID: SCR 012954) [45] to search against the Repbase database [46]. For *de novo* prediction, RepeatScout (http://www.repeatmasker.org/) (RepeatScout, RRID:SCR 014653) [47], RepeatModeler (http://www.repeatmasker.org/RepeatModeler/) RepeatModeler (RRID:SCR_015027), and LTR_Finder (http://tlife.fudan.edu.cn/tlife/ltr_finder/) (LTR_Finder, RRID:SCR_015247) [48] were used to identify *de novo* involved repeats. Tandem repeats were also *de novo* predicted using Tandem Repeats Finder (TRF) [49].

Telomere sequences were identified by BLASTN searches of both ends of the pseudochromosomes using four tandem repeats of the telomere repeat motif (TTTAGGG) with e-value cut-off of 0.003.

Gene annotations

A combination of three approaches, including homology-based prediction, *de novo* prediction and transcriptome-based prediction, was used to predict the protein-coding genes within *P. salicina* genome. For homology-based prediction, the homologous protein sequences of *Prunus persica*, *Prunus avium*, *Prunus mume*, *Pyrus bretschneideri*, *Malus domestica*, *Fragaria vesca* and *Arabidopsis thaliana* were obtained from NCBI database and mapped onto the *P. salicina* genome using TblastN

(TBLASTN; RRID:SCR_011822) (E-value ≤ 1e-5) [50], and then the matching proteins were aligned to the homologous genome sequences for accurate spliced alignments with GeneWise (GeneWise, RRID:SCR 015054) [51] to define gene models. For *de novo* prediction, Augustus (Augustus, RRID: SCR_008417) [52], GlimmerHMM (GlimmerHMM, RRID: SCR_002654) [53], SNAP (SNAP, RRID: SCR 002127) [54], GeneID (GeneID, RRID: SCR 002473) [55] and Genescan (Genescan, RRID: SCR_012902) [56] were used to predict the coding regions of genes. For transcriptome-based predictions, RNA-seq data from six tissues were used for genome annotation, processed by HISAT2 (HISAT2, RRID: SCR_015530) [42] and Stringtie (StringTie, RRID: SCR_016323) [57]. RNA-seq data were also *de novo* assembled with Trinity (Trinity, RRID: SCR_013048) [58]. The assembled sequences were aligned against *P. salicina* genome with PASA (Program to Assemble Spliced Alignment, PASA, RRID: SCR_014656) [59], and the effective alignments were assembled to gene structures. Gene models predicted by all of the methods were integrated by EVidenceModeler (EVidenceModeler, RRID: SCR_014659) [59]. To update the gene models, PASA was further used to generate UTRs [59].

Gene functions

The functional annotation of protein-coding genes within *P. salicina* genome was carried out by aligning protein sequences against SwissProt [60] and NR databases using BLASTp (with a threshold of E-value ≤ 1e-5). The protein motifs and domains were annotated by searching against InterPro (InterPro, RRID: SCR 006695) [61] and Pfam (Pfam, RRID: SCR_004726) database [62] with InterProScan (InterProScan, RRID: SCR_005829) [63]. Gene Ontology (GO) terms for each gene were retrieved according to the corresponding InterPro entry. KEGG pathway was mapped by the constructed gene set to identify the best match for each gene [64].

Non-coding RNA annotation

The tRNAs were predicted using the program tRNAscan-SE (tRNAscan-SE, RRID: SCR 010835) [65], and rRNA genes were annotated using BLASTN (BLASTN,

RRID: SCR_001598) tool with E-value of 1e-5 against rRNA sequences from several relative plant species. miRNA and snRNA were identified by searching against the Rfam (Rfam, RRID:SCR_007891) database [66] with default parameters using the INFERNAL software (INFERNAL, RRID:SCR 011809)[67].

Gene family construction

OrthoFinder version 2.3.3 (OrthoFinder, RRID:SCR_017118) [68] was used to classify the orthogroups of proteins from *P. salicina* and 16 other sequenced rosids species, including *P. armeniaca*, *P. mume*, *P. persica*, *P. dulcis*, *P. avium*, *P. yedoensis*, *M. domestica*, *P. bretschneideri*, *Pyrus communis*, *F. vesca*, *Potentilla micrantha*, *Rosa chinensis*, *Rosa multiflora*, *Rubus occidentalis*, *Morus notabilis* and *A. thaliana*.

Phylogenetic tree and divergence time estimation

For phylogenetic tree construction, proteins of single-copy orthogroups (i.e., the orthogroups which contain none or only one genes for each species) presented in at least 70% of species were selected and aligned with MAFFT version 6.846b (MAFFT, RRID: SCR 011811) [69]. After determination of the best substitution model for each orthogroup with IQ-TREE version 1.7-beta12 (IQ-TREE, RRID: SCR_017254) [70], the maximum likelihood phylogenetic tree across the 17 plant species was constructed using IQ-TREE with the parameter (-p -bb 1000), setting *A. thaliana* as outgroup.

The divergence time of each node in the phylogenetic tree was estimated with Bayesian Evolutionary Analysis Sampling Trees (BEAST, RRID: SCR_010228) [71].Two fossil constraints and a secondary calibration node were applied. The fossil *Prunus wutuensis* (age: Early Eocene, minimum age of 55.0 Mya) and the fossil *Rubus acutiformis* (age: Middle Eocene, minimum age of 41.3Mya) were placed at the stem *Prunus* and *Rubus*, respectively [72]. For the secondary calibration node, the divergence of Rosoideae and Amygdaloideae at 100.7 Mya was dated according to Xiang et al. [72]. The Markov chain Monte Carlo was reported 10,000,000 times with 1000 steps.

Gene family expansion and contraction analysis

For gene family expansion and contraction analysis, the ancestral gene content of each cluster at each node was investigated with CAFÉ version 3.1 (CAFÉ, RRID: SCR_005983) [73], basing on the phylogeny and gene numbers per orthogroup in each species, the gene family expansions/contractions at each branch were determined with *p*-value < 0.001.

Genome synteny analysis

A Python version of MCScan (minspan=100; MCScan, RRID: SCR_017650; https://github.com/tanghaibao/jcvi/wiki/MCscan) was employed to analyze the synteny between the *P. salicina* genome and other genomes within *Prunus* following the approaches of Haibao Tang [74].

Positively selected gene analysis

The ratios of nonsynonymous to synonymous substitutions (*Ka/Ks*) were calculated using the Codeml program with the free-ratio model as implemented in the PAML (PAML, RRID: SCR_014932) package [75]. The positive selection analysis was performed using the Codeml program with the optimized branch-site model as implemented in the PAML package. The positively selected genes were subjected to GO functional annotation.

Gene Ontology enrichment analysis

The Gene Ontology (GO) enrichment analysis for the specific groups of genes (e.g. tandem duplication and expanded genes) were performed using R package 'topGO' [76], setting all *P. salicina* genes as background. The lowest-level GO terms under enrichment (*p*-value < 0.01) were focused, and *p*-value was calculated using a 'classic' algorithm with the 'fisher' test. The lowest-level GO terms was based on the directed acyclic graph (DAG) of GO, with the parameter 'nodeSize = 100'.

The identification of the DUF579 family members

For the identification of the DUF579 family members, the hidden Markov model (HMM) profile corresponding to the DUF579 domain (PF04669) was downloaded from Pfam database (http://pfam.sanger.ac.uk/), and subsequently exploited for the genome of *P. salicina*, *P. persica*, *P. mume*, *P. armeniaca*, *P. dulcis* and *A. thaliana* using HMMER 3.0. The default parameters were employed and the cutoff value was set to 0.01.

Results and Discussion

Genome sequencing and assembly

We sequenced and assembled the genome of *P. salicina* using a combination of short-read sequencing from Illumina Hiseq, SMRT sequencing from PacBio and Hi-C technology. For the Illumina sequencing, a total of approximately 26.6 Gb (85.4 × coverage) short reads was obtained (Table S1). A total of ~53.0 Gb long-sequencing reads were generated by PacBio Sequel platform. After removing adaptors within sequences, about 52.9 Gb (169.7 × coverage) subreads were obtained (Table S1). The subreads have a mean length of 13.2 kb (Table S2). About 59.1 Gb (189.5 × coverage) sequencing data generated from Hi-C library was produced (Table S1). The quality of Hi-C sequencing was evaluated with HiCUP [33], and the effect rate was approximately 28.10% (Table S3).

In the genome assembly process, Illumina sequencing data were used for the genome survey and polishing of preliminary contigs, PacBio long reads were used for contig assembly and Hi-C reads were used for chromosome-level scaffolding. Based on the total number of k-mers (19,341,904,177), the estimated *P. salicina* genome size was calculated to be approximately 311.82 Mb (Figure S1). The heterozygous and repeat sequencing ratios were 0.70% and 54.49%, respectively (Table S4). The *de novo* genome assembly of *P. salicina with a* total length of 284.2 Mb (Table 1) was yielded. As shown in Fig. 1, the Hi-C assisted genome assembly was anchored onto the eight pseudochromosomes with lengths ranging from 23.70 to 54.53 Mb (Table S5). Five regions of tandemly repeated telomeric repeat sequences were identified on

three pseudochromosomes (Table S5). The total length of pseudochromosomes accounted for 96.56% of the genome sequences (Figure 1), with contig N50 of 1.78 Mb and scaffold N50 of 32.32 Mb (Table1; Table S6).

Evaluation of the genome assembly

To assess the genome assembly quality, the Illumina clean data were aligned to the *P. salicina* genome, with the mapping rate of 96.93%. A total of 98.81% assembled genome was covered by the reads and the mapping coverage with at least 4×, 10×, 20×was 98.48 %, 98.06% and 97.13%, respectively (Table1; Table S7). The RNA-seq reads were mapped against the genome assembly, and the percentage of aligned reads ranged from 92.44% to 95.25% (Table1; Table S8). A total of 3,668 homozygous SNPs were identified, accounting for only 0.0015% of the reference genome (Table S9). The low rate of homozygous SNPs suggested that the assembly had a high base accuracy. 234 Core Eukaryotic Genes (CEGs) out of the complete set of 248 CEGs (94.35%) were covered by the assembly, and 229 (92.34%) of these were complete (Table1; Table S10). BUSCO analysis based on single copy orthologs set showed that 95.7% of the expected genes were identified as complete, 1.3% were fragmented, and only 3.0% were missing (Table1; Table S11). These results verified the high quality of the presently generated *P. salicina* genome assembly

Genome annotation

The results of the repeat annotations found that 48.28% of the assembly was covered with transposable elements (TE). Among them, long terminal repeat (LTR) retrotransposons represented the greatest proportion, making up 42.10% of the genome (Table1; Table S12). The TE percentage and density of duplicates resulted from tandem duplications were shown in Figure 1. Tandem duplicates occurred for 9.8% of the genes (Table 1) and were preferentially enriched in ‘transferase activity (GO: 0016758 and GO: 0016747)’ and ‘phloem development (GO: 0010088)’ (Figure S2). The significant enrichment of the sieve element occlusion genes in ‘phloem development’, which were involved in wound sealing of the phloem [77], might be

associated with specific requirements during the damage response in *P. salicina.*

For gene annotations, we predicted 24,448 non-redundant protein-coding genes in *P. salicina*. There were 24,209 genes (~99.0%) that could be assigned to eight pseudochromosomes (Table 1), and the gene density was shown in Figure 1. The average number of exons per gene, and average CDS length were 4.97 and 1,157.42, respectively (Table 2). Further gene functional annotation showed that 23,931 (97.9%) protein-coding genes were successfully annotated (Table 1; Table S13). For the identification of non-coding RNA (ncRNA) genes, a total of 627 miRNA, 960 tRNA, 273 rRNA and 2,023 snRNA in the *P. salicina* genome were predicted (Table S14).

Evolution of the *P. salicina* genome

The genome sequences of the representative sequenced rosid species were collected and subjected to comparative genomic analysis with *P. salicina* to reveal the genome evolution and divergence of *P. salicina.* A total of 15,751 orthogroups containing 23,265 genes were found in *P. salicina*. Moreover, 1,010 genes which were specific to *P. salicina* were identified. A comparison of the predicted proteomes among the 17 species indicated that 9,616, 10,447, 11,098, 13,963 and 15,512 orthogroups were shared between *P. salicina* and Rosids, Rosales, Rosaceae, Amygdaloideae and *Prunus*, respectively.

The phylogenetic analysis confirmed the close relationship among *P. salicina*, *P. mume* and *P. armeniaca*. The molecular clock of these plant genomes was also calculated. The data indicated that *P. salicina* diverged from the ancestor of *P. mume* and *P. armeniaca* approximately 9.05 Mya, from the ancestor of *P. persica* and *P.dulcis* 11.12 Mya (Figure 2).

We also explored the genome syntenic blocks between *P. salicina* and the other representative *Prunus* species. As shown in Fig. 3, our genome assembly of *P. salicina* exhibited a high level of genome synteny with all the other *Prunus* genomes, especially the genomes of *P. avium* and *P. dulcis*. Significantly fewer inversions were found in *P. salicina* vs *P. avium* and *P. salicina* vs *P. dulcis* than that in *P. salicina* vs *P. mume* and *P. salicina* vs *P. armeniaca.*

Expansion and contraction of gene families in *P. salicina*

The gene family analysis showed that during the evolution of *P. salicina*, 146 gene families were expanded and 500 gene families were contracted. The functional enrichment on Gene Ontology of those expanded gene families identified 60 significantly enriched GO terms (p-value < 0.05) (Table S15; Figure S3).

It was noteworthy that genes from the expanded families were enriched in a series of cell wall related processes, such as 'cell wall polysaccharide metabolic process (GO: 0010383)', 'hemicellulose metabolic process (GO: 0010410)' and 'regulation of cellular biosynthetic process (GO: 0031326)'. Specially, genes in 'xylan biosynthetic process (GO: 0045492)', which corresponded to the DUF579 family [78], were significantly expanded. Further investigation showed that the major copy differences were found in Clade II, which consisted of orthologs of IRX15/IRX15L [78], with seven members in *P. salicina* and only two to four members in other *Prunus* species (Figure 4). It was reported that IRX15 and IRX15L defined a new class of genes involved in xylan biosynthesis [79, 80]. The species-specific expansion of this new subclade might contribute to the relatively high content of xylan-related metabolites (like xylose and xyliot) in plum [9, 10], which provided new insight into the xylan metabolism in plum.

Moreover, the FRS (FAR1-related sequence) gene family, which played multiple roles in a wide range of cellular processes [81], was also significantly expanded in the phylogeny (GO: 000945), and the family expansion may be related to the genetic and phenotypic diversity in *P. salicina*.

Positively selected genes in *P. salicina*

The Ka/Ks ratios for all the 2,314 single-copy orthologs shared with the sequenced *Prunus* species were calculated. A total of 213 candidate genes in *P. salicina* underwent positive selection ($P<0.05$). Most of them were enriched in the GO terms involved in 'monooxygenase activity (GO: 0004497)' and 'enzyme inhibitor activity (GO: 0004857)' (Figure S4). It was noteworthy that the category 'monooxygenase

activity' was also found in the enriched GO terms for the expanded gene families in *P. salicina*, which might provide valuable candidate genes for further functional investigations.

Conclusions

To our knowledge, this is the first report of the chromosome-level genome assembly of plums using Illumina and PacBio sequencing platforms with Hi-C technology. The assembly had a total size of 284.2 Mb, the contig and scaffold N50 reached 1.8 Mb and 32.3 Mb, respectively. A total of 24,448 protein-coding genes were predicted, and 23,931 genes (97.9%) have been annotated. Phylogenetic analysis indicated that *P. salicina* was closely related to *P. mume* and *P. armeniaca*. Expanded gene families in *P. salicina* were significantly enriched in several cell-wall related processes. Remarkably, the *P. salicina*-specific expansion of the xylan biosynthesis-related DUF579 family provided new insight into the xylan metabolism in plums. Given the economic and evolutionary importance of *P. salicina*, the genomic data in this study offer a valuable resource for facilitating plum breeding programs and studying the genetic basis for agronomic and adaptive divergence of plum and *Prunus* species.

Availability of supporting data and materials

This Whole Genome Shotgun project has been deposited at DDBJ/ENA/GenBank under the accession WERZ00000000. The version described in this paper is version WERZ01000000. The raw sequencing data are available through the NCBI Sequence Read Archive (SRA) via accession numbers from SRR10233497 to SRR10233505, via the Project PRJNA574159. The transcriptome data are available through the NCBI SRA (from SRR10235674 to SRR10235679). The genome data have also been submitted to Genome Database for Rosaceae (Accession number: tfGDR1044). All the annotation tables containing results of an analysis of the draft genome are available at Figshare (https://doi.org/10.6084/m9.figshare.9973469).

Additional files

Table S1 Statistics of *P. salicina* genome sequencing data.

Table S2 Statistics of characteristics of PacBio long-reads.

Table S3 Statistics of Hi-C sequencing data.

Table S4 Estimation of the genome size using k-mer analysis.

Table S5 Summary of assembled 8 pseudochromosomes of *P. Salicina.*

Table S6 Summary of the genome assembly of *P. Salicina.*

Table S7 Statistics of mapping ratio in genome.

Table S8 Summary of the transcriptome and their mapping rate on the genome assembly.

Table S9 Number and density of SNPs in *P. salicina* genome.

Table S10 Assessment of CEGMA.

Table S11 Summary of BUSCO analysis results according to prediction.

Table S12 Detailed classification of repeat sequences.

Table S13 Statistics of functional annotation.

Table S14 Summary of non-coding RNA.

Table S15 List of the Gene ontology terms significantly enriched in the expanded gene families of *P. salicina*

Figure S1 17-mer frequency distribution in *P. salicina* genome.

Figure S2 Gene ontology enrichment of the tandemly duplicated genes in *P. salicina*.

Figure S3 Gene ontology enrichment of *P. salicina*-expanded genes.

Figure S4 Gene ontology enrichment of the positively selected genes in *P. salicina*.

Authors' Contributions

Y.H.H. conceived the study. C.Y.L., C.F. and J.T.W. performed bioinformatics analysis. W.Z.P., J.J.H. and J.J.P. collected the samples and extracted the DNA. C.Y. L. and C. F. wrote the manuscript. All authors read and approved the final manuscript.

Abbreviations

BLAST: Basic Local Alignment Search Tool; BEAST: Bayesian Evolutionary Analysis Sampling Trees; bp: base pair; BUSCO: Benchmarking Universal Single-Copy Orthologs; CEGMA: Core Eukaryotic Genes Mapping Approach; CTAB: cetyltrimethylammonium bromide; EVM: EVidenceModeler; Gb: gigabase pair; GO: Gene Ontology; Hi-C: high-throughput chromosome conformation capture; kb: kilobase pair; KEGG: Kyoto Encyclopedia of Genes and Genomes; Mb: megabase pair; miRNA: microRNA; Mya: million years ago; NCBI: National Center for Biotechnology Information; PacBio: Pacific Biosciences; PAML: phylogenetic analysis by maximum likelihood; PASA: Program to Assemble Spliced Alignments; RNA-seq: RNA sequencing; rRNA: ribosomal RNA; SMRT: single-molecule real-time; SnRNA，small nuclear RNA; SNP: single-nucleotide polymorphism; TRF: Tandem Repeats Finder; tRNA: transfer RNA.

Funding

This work was financially supported by The Industry University Research Collaborative Innovation Major Projects of Guangzhou Science Technology Innovation Commission (201704020021) and Modern Agricultural Industry Technology System of Guangdong Province (2016LM1128).

Competing interests

The authors declare no competing interests.

Figure Legends

Figure 1 The genome and photograph of *P. salicina*. Landscape of the *P. salicina* genome, comprising 8 pseudochromosomes that cover ~96.56% of assembly (A); Concentric circles, from outermost to innermost, showing TE percentage (red; B); gene density (green; C); density of duplicates resulted from tandem duplications (blue; D); (E) photograph of *P. salicina*.

Figure 2 Evolution of *P. salicina* genome and orthogroups. (A) The phylogeny, divergence time and orthogroup expansions/contractions for 17 rosids species. The tree was constructed by maximum likelihood method using 341 single copy orthogroups. All nodes have 100% bootstrap support. Divergence time was estimated on a basis of three calibration points (blue circles). Blue bar indicates 95% HPD (highest posterior density) for each node. The numbers in red and green indicate the numbers of orthogroups that have expanded and contracted along particular branches, respectively. (B) The comparison of genes among 17 rosids. The grey bars indicate the genes belonging to 9,616 rosids-shared orthogroups in each of 17 rosids. The grey + green bars indicate the genes belonging to 10,447 rosales-shared orthogroups in each of 16 rosales. The grey + green + pink bars indicate the genes belonging to 11,098 Rosaceae-shared orthogroups in each of 15 Rosaceae. The grey + green + pink + yellow bars indicate the genes belonging to 13,963 rosaceae-shared orthogroups in each of ten Amygdaloideae. The grey + green + pink + yellow + blue bars indicate the genes belonging to 15,512 *Prunus*-shared orthogroups in each of seven *Prunus* species. The red and stripe bars indicate the genes in species-specific orthogroups and unassigned genes, respectively. The white bars indicate the remaining genes for each genome.

Figure 3 Chromosome-level collinearity patterns between *P. salicina*, *P. mume* and *P. armeniaca* (A) and between *P. salicina*, *P. avium* and *P. dulcis* (B). The numbers indicate the pseudochromosome order generated from the original genome sequence.

The pseudochromosome 1 and 8 in *P. avium* and *P. dulcis* are reversed. Each gray line represents one block. The inverted regions are highlighted with brown color.

.

Figure 4 The significant expansion of the DUF579 family members in *P. salicina*. (A) Phylogenetic tree of the DUF579 proteins from *P. salicina* (red cicle), *P. persica* (hollow inverted triangle), *P. mume* (solid triangle), *P. armeniaca* (hollow diamond), *P. dulcis* (solid diamond) and *A. thaliana* (solid square). (B) The summary of the numbers of clade members in DUF579 family.

Table 1 Summary of genome assembly and annotation for *P. salicina*

	**Number or percentage**
**Assembly feature**	
Total length of scaffolds (bp)	284,209,110
Number of scaffolds	75
N50 of scaffolds (bp)	32,324,625
Total length of contigs (bp)	284,189,410
Number of contigs	272
N50 of contigs (bp)	1,777,944
Mapping rate by reads from short-insert libraries	96.93%
Assembled CEGs	94.35%
Completely assembled CEGs	92.34%
Complete BUSCOs	95.7%
Complete and single-copy BUSCOs	86.5%
Complete and duplicated BUSCOs	9.2%
Fragmented BUSCOs	1.3%
Missing BUSCOs	3.0%
RNA-Seq evaluation	92.44%-95.25%
**Genome annotation**	
Percentage of transposable elements (TE)	48.28%
Percentage of long terminal repeat (LTR) retrotransposon	42.1%
No. of predicted protein-coding genes	24,448
No. of genes assigned to pseudochromosomes	24,209 (99.0%)
No. of genes annotated to public database	23,930 (97.9%)
No. of genes annotated to GO database	13,484 (55.2%)
No. of genes duplicated by tandem duplications	2,384(9.8%)

Table 2 Statistics of predicted protein-coding genes.

Gene set		Number	Average transcript length (bp)	Average CDS length (bp)	Average exons per gene	Average exons length (bp)	Average intron length (bp)
De novo prediction	Augustus	23,592	2,627.71	1167.83	4.80	243.43	384.45
	GlimmerHMM	39,985	5,450.51	747.07	3.14	238.12	2200.59
	SNAP	24,882	2,876.50	728.45	4.22	172.73	667.66
	Geneid	33,780	3,829.40	899.99	4.44	202.74	851.78
	Genscan	21,882	8,251.09	1355.87	6.34	213.98	1292.13
Homolog prediction	*Pyrus bretschneideri*	20,265	3,119.83	1356.17	4.74	286.35	472.06
	Malus domestica	20,010	2,920.17	1361.30	4.65	292.56	426.72
	Prunus mume	23,064	3,038.66	1346.19	4.78	281.67	447.84
	Prunus persica	28,915	2,296.51	1099.56	4.06	270.55	390.64
	Arabidopsis thaliana	28,284	2,071.73	973.28	3.67	265.51	412.07
	Fragaria vesca	22,927	2,994.24	1380.61	4.59	300.66	449.24
	Prunus avium	22,715	3,077.20	1351.28	4.74	284.86	461.03
RNA-seq	PASA	196,264	3,913.86	1008.68	5.16	195.60	698.88
	Transcripts	42,450	11,076.28	2360.92	6.85	344.83	1490.64
EVM		27,981	2,736.70	1061.73	4.57	232.52	469.68
PASA-update*		27,594	2,784.15	1092.82	4.64	235.59	464.83
Final set*		24,448	2,988.45	1157.42	4.97	233.09	461.72

* UTR regions were contained

References

1. Roussos PA, Efstathios N, Intidhar B, Denaxa N-K and Tsafouros A. Plum (*Prunus domestica* L. and *P. salicina* Lindl.). In: Monique Simmonds VRP, editor. Nutritional Composition of Fruit Cultivars. Elsevier; 2016. p. 639 - 666.
2. Topp BL, Russell DM, Neumüller M, Dalbó MA and Liu W. Plum. In: Maria Luisa Badenes DHB, editor. Fruit Breeding. Springer; 2012. p. 571-621.
3. Hartmann W and Neumüller M. Plum breeding. In: Shri Mohan Jain PMP, editor. Breeding Plantation Tree Crops: Temperate Species. Springer; 2009. p. 161-231.
4. Okie W and Hancock J. Plums. In: Hancock JF, editor. Temperate Fruit Crop Breeding. Springer Science & Business Media; 2008. p. 337-358.
5. Esmenjaud D and Dirlewanger E. Plum. In: Kole C, editor. Genome Mapping and Molecular Breeding in Plants. Springer; 2007. p. 119-135.
6. Guerra M and Rodrigo J. Japanese plum pollination: A review. SCI Hortic-Amsterdam 2015;**197**:674-686.
7. Rennie EA and Scheller HV. Xylan biosynthesis. Curr Opin Biotech 2014;**26**:100-107.
8. Brummell DA and Schröder R. Xylan metabolism in primary cell walls. NZ J Forestry Sci. 2009;**39**:125-143.
9. Renard CMGC and Ginies C. Comparison of the cell wall composition for flesh and skin from five different plums. Food Chem 2009;**114**(3):1042-1049.
10. Arcaño YD, García ODV, Mandelli D, Carvalho WA and Pontes LAM. Xylitol: A review on the progress and challenges of its production by chemical route. Catal Today 2020;**344**:2-14.
11. Aranzana MJ, Decroocq V, Dirlewanger E, Eduardo I, Gao ZS, Gasic K, et al. *Prunus* genetics and applications after *de novo* genome sequencing: achievements and prospects. Hortic Res 2019;**6** (1):1-25.
12. Velasco R, Zharkikh A, Affourtit J, Dhingra A, Cestaro A, Kalyanaraman A, et al. The genome of the domesticated apple (*Malus* × *domestica* Borkh.). Nat Genet 2010;**42** (10):833-839.
13. Chen X, Li S, Zhang D, Han M, Jin X, Zhao C, et al. Sequencing of a wild apple (*Malus baccata*) genome unravels the differences between cultivated and wild apple species regarding disease resistance and cold tolerance. G3: Genes, Genomes, Genet 2019;**9** (7):2051-2060.
14. Zhang L, Hu J, Han X, Li J, Gao Y, Richards CM, et al. A high-quality apple genome assembly reveals the association of a retrotransposon and red fruit colour. Nat Commun 2019;**10** (1):1-13.
16. Verde I, Abbott AG, Scalabrin S, Jung S, Shu S, Marroni F, et al. The high-quality draft genome of peach (*Prunus persica*) identifies unique patterns of genetic diversity, domestication and genome evolution. Nat Genet 2013;**45**(5):487-494.
17. Linsmith G, Rombauts S, Montanari S, Deng CH, Celton J-M, Guérif P, et al.

Pseudo-chromosome–length genome assembly of a double haploid “Bartlett” pear (*Pyrus communis* L.) GigaSciemce 2019; 8 (12):giz138.

18. Wu J, Wang Z, Shi Z, Zhang S, Ming R, Zhu S, et al. The genome of the pear (*Pyrus bretschneideri* Rehd.). Genome Res 2013;**23**(2):396-408.
19. Chagné D, Crowhurst RN, Pindo M, Thrimawithana A, Deng C, Ireland H, et al. The draft genome sequence of European pear (*Pyrus communis* L.‘Bartlett’). PloS One 2014;**9** (4):e92644.
20. Dong X, Wang Z, Tian L, Zhang Y, Qi D, Huo H, et al. *De novo* assembly of a wild pear (*Pyrus betuleafolia*) genome. Plant Biotechnol J 2020;**18**(2):581-595
21. Shulaev V, Sargent DJ, Crowhurst RN, Mockler TC, Folkerts O, Delcher AL, et al. The genome of woodland strawberry (*Fragaria vesca*). Nat Genet 2011;**43**(2):109-116.
22. Edger PP, Poorten TJ, VanBuren R, Hardigan MA, Colle M, McKain MR, et al. Origin and evolution of the octoploid strawberry genome. Nat Genet 2019;**51**(3):541-547.
23. Alioto T, Alexiou KG, Bardil A, Barteri F, Castanera R, Cruz F, et al. Transposons played a major role in the diversification between the closely related almond and peach genomes: Results from the almond genome sequence. Plant J 2020;**101**(2):455-472.
24. Sánchez-Pérez R, Pavan S, Mazzeo R, Moldovan C, Cigliano RA, Del Cueto J, et al. Mutation of a bHLH transcription factor allowed almond domestication. Science 2019; **364** (6445):1095-1098.
25. VanBuren R, Bryant D, Bushakra JM, Vining KJ, Edger PP, Rowley ER, et al. The genome of black raspberry (*Rubus occidentalis*). Plant J 2016;**87**(6):535-547.
26. Shirasawa K, Isuzugawa K, Ikenaga M, Saito Y, Yamamoto T, Hirakawa H, et al. The genome sequence of sweet cherry (*Prunus avium*) for use in genomics-assisted breeding. DNA Res 2017;**24**(5):499-508.
27. Wang J, Liu W, Zhu D, Hong P, Zhang S, Xiao S, et al. Chromosome-scale genome assembly of sweet cherry *(Prunus avium* L.) cv. Tieton obtained using long-read and Hi-C sequencing. Hort Res 2020;**7** (1):1-11.
28. Jiang F, Zhang J, Wang S, Yang L, Luo Y, Gao S, et al. The apricot (*Prunus armeniaca* L.) genome elucidates Rosaceae evolution and beta-carotenoid synthesis. Hortic Res 2019;6 (1):1-12.
29. Campoy JA, Sun H, Goel M, Jiao W-B, Folz-Donahue K, Kukat C, et al. Chromosome-level and haplotype-resolved genome assembly enabled by high-throughput single-cell sequencing of gamete genomes. BioRxiv. 2020.
30. Jiang S, An H, Xu F and Zhang X. Chromosome-level genome assembly and annotation of the loquat (*Eriobotrya japonica*) genome. GigaScience 2020;**9**(3):giaa015.
31. Zhang Q, Chen W, Sun L, Zhao F, Huang B, Yang W, et al. The genome of *Prunus mume*. Nat Commun 2012;**3**:1318.
32. Lodhi MA, Ye G-N, Weeden NF and Reisch BI. A simple and efficient method for DNA extraction from grapevine cultivars and *Vitis* species. Plant Mol Biol Rep. 1994;**12** (1):6-13.

33. Wingett S, Ewels P, Furlan-Magaril M, Nagano T, Schoenfelder S, Fraser P, et al. HiCUP: pipeline for mapping and processing Hi-C data. F1000Res 2015;**4**:1310.
34. Luo R, Liu B, Xie Y, Li Z, Huang W, Yuan J, et al. SOAPdenovo2: an empirically improved memory-efficient short-read *de novo* assembler. Gigascience 2012;**1** (1):2047-217X-1-18.
35. Chin C-S, Peluso P, Sedlazeck FJ, Nattestad M, Concepcion GT, Clum A, et al. Phased diploid genome assembly with single-molecule real-time sequencing. Nat Methods 2016;**13** (12):1050-1054.
36. Chin C-S, Alexander DH, Marks P, Klammer AA, Drake J, Heiner C, et al. Nonhybrid, finished microbial genome assemblies from long-read SMRT sequencing data. Nat Methods 2013;**10**(6):563-569.
37. Walker BJ, Abeel T, Shea T, Priest M, Abouelliel A, Sakthikumar S, et al. Pilon: an integrated tool for comprehensive microbial variant detection and genome assembly improvement. PloS One 2014;**9** (11):e112963.
38. Roach MJ, Schmidt SA and Borneman ARJBb. Purge Haplotigs: allelic contig reassignment for third-gen diploid genome assemblies. BMC Bioinformatics 2018;**19** (1):460.
39. Li H and Durbin R. Fast and accurate short read alignment with Burrows-Wheeler transform. Bioinformatics 2009;**25** (14):1754-1760.
40. Burton JN, Adey A, Patwardhan RP, Qiu R, Kitzman JO and Shendure J. Chromosome-scale scaffolding of *de novo* genome assemblies based on chromatin interactions. Nat Biotechnol 2013;**31** (12):1119-1125.
41. Robinson JT, Turner D, Durand NC, Thorvaldsdottir H, Mesirov JP and Aiden EL. Juicebox. js provides a cloud-based visualization system for Hi-C data. Cell Syst 2018;**6**(2):256-258. e1.
42. Kim D, Langmead B and Salzberg SL. HISAT: a fast spliced aligner with low memory requirements. Nat Methods 2015;**12** (4):357-360.
43. Parra G, Bradnam K and Korf I. CEGMA: a pipeline to accurately annotate core genes in eukaryotic genomes. Bioinformatics 2007;**23** (9):1061-1067.
44. Simão FA, Waterhouse RM, Ioannidis P, Kriventseva EV and Zdobnov EM. BUSCO: assessing genome assembly and annotation completeness with single-copy orthologs. Bioinformatics 2015;**31**(19):3210-3212.
45. Tarailo−Graovac M and Chen N. Using RepeatMasker to identify repetitive elements in genomic sequences. Curr Protoc Bioinf 2009;**25** (1):4-10.
46. Jurka J, Kapitonov VV, Pavlicek A, Klonowski P, Kohany O and Walichiewicz J. Repbase Update, a database of eukaryotic repetitive elements. Cytogenet Genome Res 2005;**110** (1-4):462-467.
47. Price AL, Jones NC and Pevzner PA. *De novo* identification of repeat families in large genomes. Bioinformatics 2005;**21** (suppl_1):i351-i358.
48. Xu Z and Wang H. LTR_FINDER: an efficient tool for the prediction of full-length LTR retrotransposons. Nucleic Acids Res 2007;**35** (suppl_2):W265-W268.

49. Benson G. Tandem repeats finder: a program to analyze DNA sequences. Nucleic Acids Res 1999;**27** (2):573-580.
50. Gertz EM, Yu Y-K, Agarwala R, Schäffer AA and Altschul SF. Composition-based statistics and translated nucleotide searches: improving the TBLASTN module of BLAST. BMC Biol 2006;**4** (1):1-14.
51. Birney E, Clamp M and Durbin R. GeneWise and genomewise. Genome Res 2004;**14** (5):988-995.
52. Stanke M, Steinkamp R, Waack S and Morgenstern B. AUGUSTUS: a web server for gene finding in eukaryotes. Nucleic Acids Res 2004;**32** (suppl_2):W309-W312.
53. Majoros WH, Pertea M and Salzberg SL. TigrScan and GlimmerHMM: two open source ab initio eukaryotic gene-finders. Bioinformatics 2004;**20** (16):2878-2879.
54. Korf I. Gene finding in novel genomes. BMC Bioinf 2004;**5** (1):59.
55. Blanco E, Parra G and Guigó R. Using geneid to identify genes. Curr Protoc Bioinf 2007;**18** (1):4.3. 1-4.3. 28.
56. Burge C and Karlin S. Prediction of complete gene structures in human genomic DNA. J Mol Biol 1997;**268** (1):78-94.
57. Pertea M, Pertea GM, Antonescu CM, Chang T-C, Mendell JT and Salzberg SL. StringTie enables improved reconstruction of a transcriptome from RNA-seq reads. Nat Biotechnol 2015;**33**(3):290-295.
58. Haas BJ, Papanicolaou A, Yassour M, Grabherr M, Blood PD, Bowden J, et al. *De novo* transcript sequence reconstruction from RNA-seq using the Trinity platform for reference generation and analysis. Nat Protoc 2013;**8** (8):1494-1512.
59. Haas BJ, Salzberg SL, Zhu W, Pertea M, Allen JE, Orvis J, et al. Automated eukaryotic gene structure annotation using EVidenceModeler and the Program to Assemble Spliced Alignments. Genome Biol 2008;**9** (1):R7.
60. Bairoch A and Apweiler R. The SWISS-PROT protein sequence database and its supplement TrEMBL in 2000. Nucleic Acids Res 2000;**28** (1):45-48.
61. Mulder N and Apweiler R. InterPro and InterProScan: tools for protein sequence classifcation and comparison. Methods Mol Biol 2007; **396**:59-70.
62. Finn RD, Bateman A, Clements J, Coggill P, Eberhardt RY, Eddy SR, et al. Pfam: the protein families database. Nucleic Acids Res 2013;**42** (D1):D222-D230.
63. Jones P, Binns D, Chang H-Y, Fraser M, Li W, McAnulla C, et al. InterProScan 5: genome-scale protein function classification. Bioinformatics 2014;**30** (9):1236-1240.
64. Kanehisa M and Goto S. KEGG: kyoto encyclopedia of genes and genomes. Nucleic Acids Res 2000;**28** (1):27-30.
65. Lowe TM and Eddy SR. tRNAscan-SE: a program for improved detection of transfer RNA genes in genomic sequence. Nucleic Acids Res 1997;**25** (5):955-964.
66. Griffiths-Jones S, Bateman A, Marshall M, Khanna A and Eddy SR. Rfam: an RNA family database. Nucleic Acids Res 2003;**31**(1):439-441.

67. Nawrocki EP and Eddy SR. Infernal 1.1: 100-fold faster RNA homology searches. Bioinformatics 2013;**29** (22):2933-2935.
68. Emms DM and Kelly S. OrthoFinder: solving fundamental biases in whole genome comparisons dramatically improves orthogroup inference accuracy. Genome Biol 2015;**16** (1):157.
69. Katoh K and Standley DM. MAFFT multiple sequence alignment software version 7: improvements in performance and usability. Mol Biol Evol 2013;**30** (4):772-780.
70. Nguyen L-T, Schmidt HA, Von Haeseler A and Minh BQ. IQ-TREE: a fast and effective stochastic algorithm for estimating maximum-likelihood phylogenies. Mol Biol Evol 2015;**32** (1):268-274.
71. Drummond AJ and Rambaut A. BEAST: Bayesian evolutionary analysis by sampling trees. BMC Evol Biol 2007;**7** (1):1-8.
72. Xiang Y, Huang C-H, Hu Y, Wen J, Li S, Yi T, et al. Evolution of Rosaceae fruit types based on nuclear phylogeny in the context of geological times and genome duplication. Mol Biol Evol 2017;**34** (2):262-281.
73. De Bie T, Cristianini N, Demuth JP and Hahn MW. CAFE: a computational tool for the study of gene family evolution. Bioinformatics 2006;**22** (10):1269-1271.
74. Tang H. Multiple collinearity scan—mcscan. 2009.
75. Yang Z. PAML 4: phylogenetic analysis by maximum likelihood. Mol Biol Evol 2007;**24**(8):1586-1591.
76. Alexa A and Rahnenführer J. Gene set enrichment analysis with topGO. Bioconductor Improv. 2009;27.
77. Ernst AM, Jekat SB, Zielonka S, Müller B, Neumann U, Rüping B, et al. Sieve element occlusion (SEO) genes encode structural phloem proteins involved in wound sealing of the phloem. P Natl Acad Sci USA 2012;**109** (28): E1980-E1989.
78. Temple H, Mortimer JC, Tryfona T, Yu X, Lopez‐Hernandez F, Sorieul M, et al. Two members of the DUF 579 family are responsible for arabinogalactan methylation in Arabidopsis. Plant Direct 2019;**3** (2):e00117.
79. Jensen JK, Kim H, Cocuron JC, Orler R, Ralph J and Wilkerson CG. The DUF579 domain containing proteins IRX15 and IRX15-L affect xylan synthesis in Arabidopsis. Plant J 2011;**66** (3):387-400.
80. Brown D, Wightman R, Zhang Z, Gomez LD, Atanassov I, Bukowski JP, et al. Arabidopsis genes IRREGULAR XYLEM (IRX15) and IRX15L encode DUF579‐containing proteins that are essential for normal xylan deposition in the secondary cell wall. Plant J 2011;**66** (3):401-413.
81. Ma L and Li G. FAR1-related sequence (FRS) and FRS-related factor (FRF) family proteins in *Arabidopsis* growth and development. Front Plant Sci 2018; **9**:692.

Figure 1

Click here to access/download;Figure;Figure 1-ok.tif

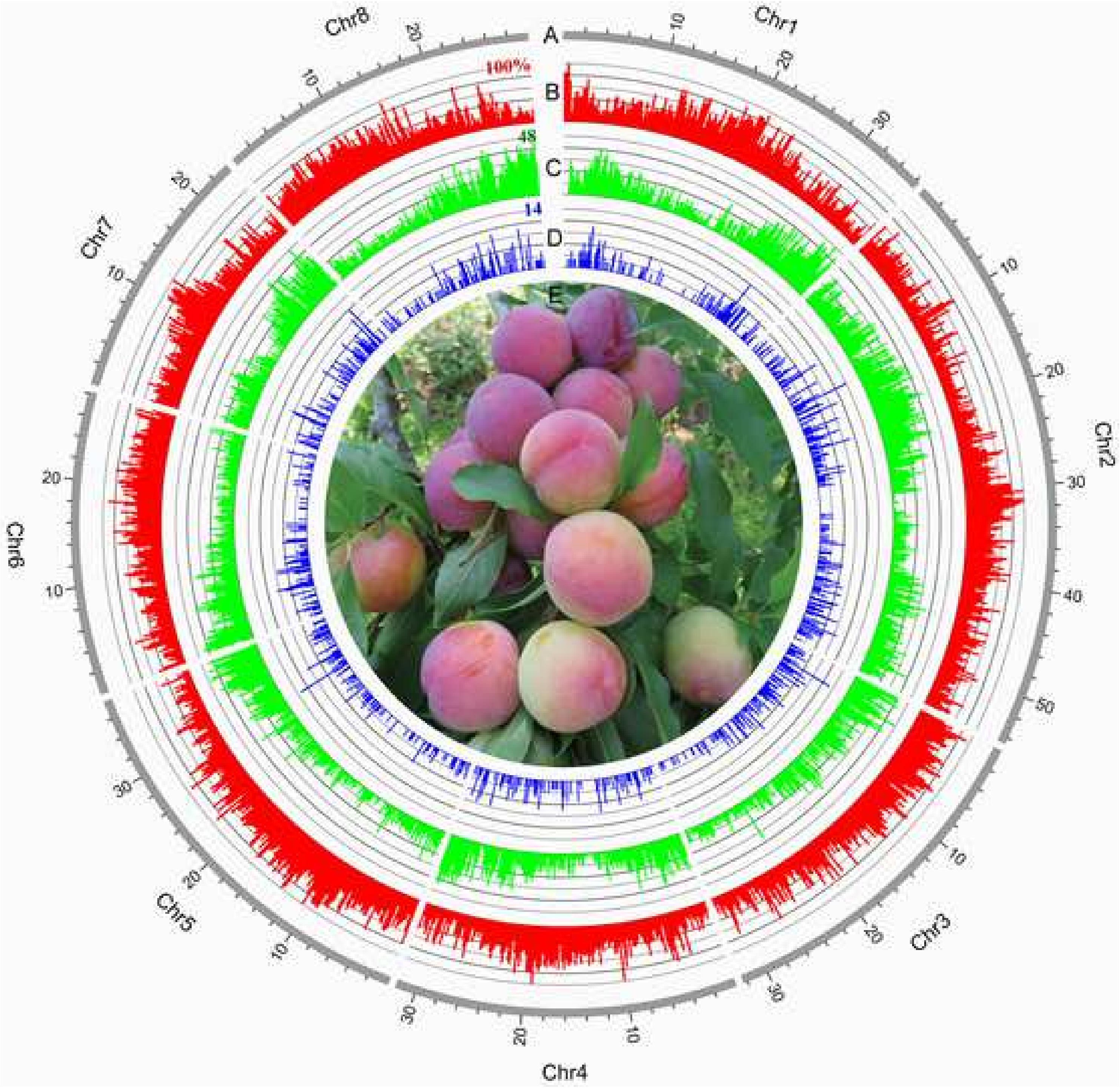

Figure 2

Click here to access/download;Figure;Figure 2-ok.tif

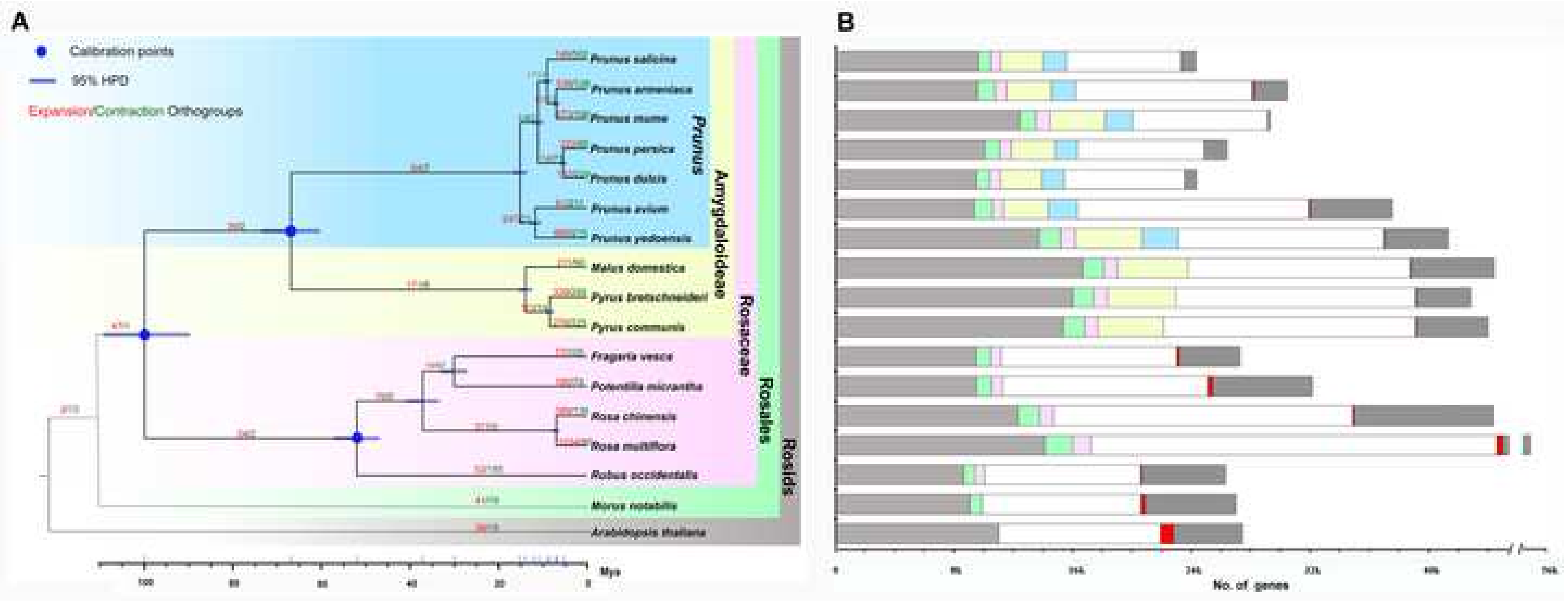

Figure 3

Click here to access/download;Figure;Figure 3.tif

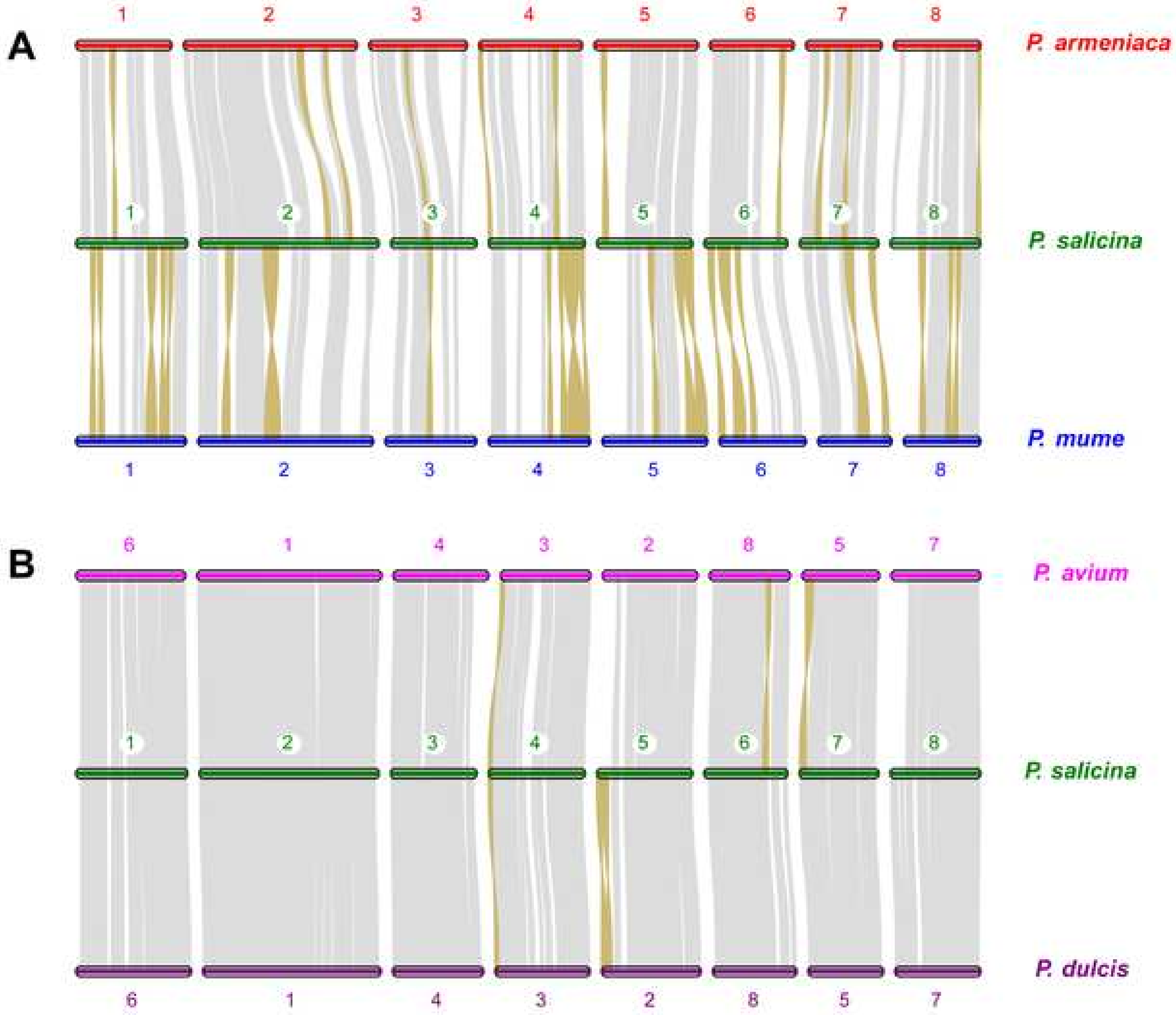

Click here to access/download;Figure;Figure 4-OK.tif

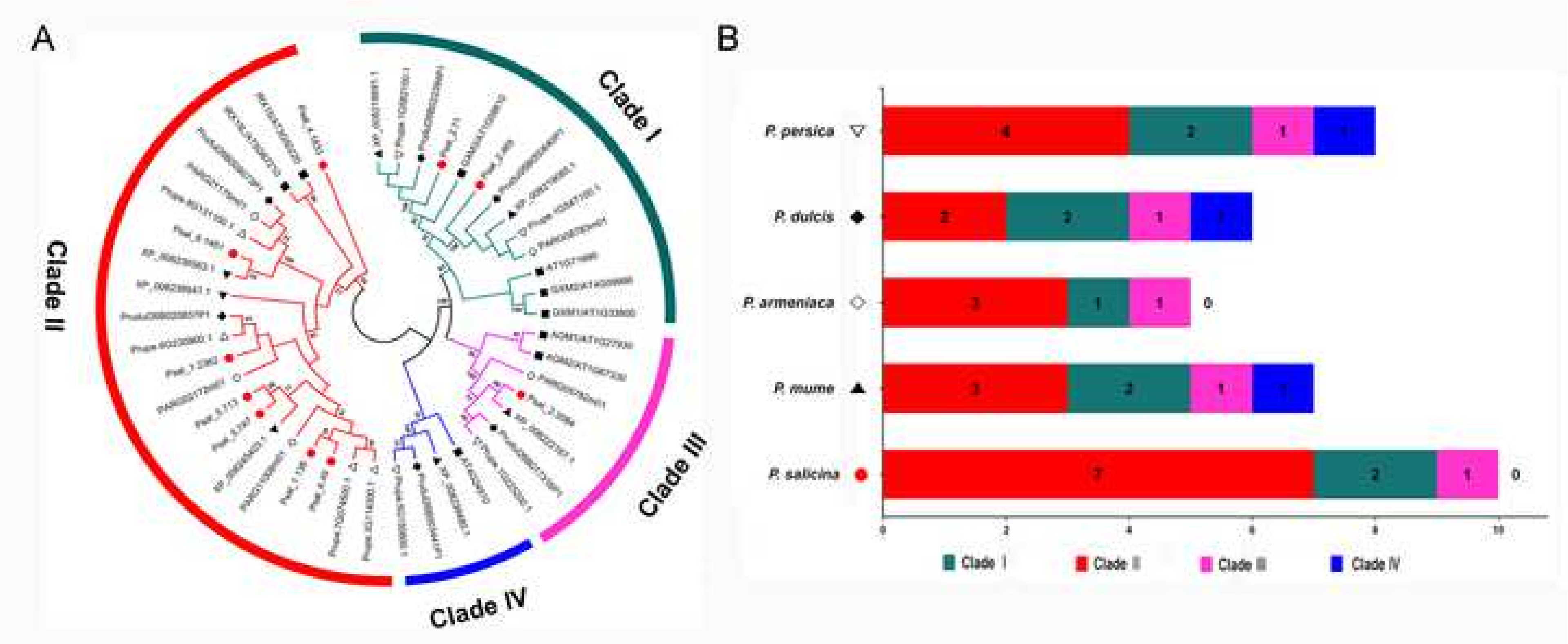

Click here to access/download
Supplementary Material
Supplementary Tables.xlsx

Click here to access/download
Supplementary Material
Supplementary Figures.pdf
