# Supplementary material for: Chromosome-level draft genome of a diploid plum (Prunus salicina)
Source: Gigascience. 2020 Dec 10;9(12):giaa130. doi: 10.1093/gigascience/giaa130 (PMC7727024; doi:10.1093/gigascience/giaa130)
Supplement: giaa130_GIGA-D-20-00195_Revision_2 [file giaa130_giga-d-20-00195_revision_2.pdf]

GigaScience

The chromosome-level draft genome of a diploid plum (Prunus salicina)

--Manuscript Draft--

**Manuscript Number:**	GIGA-D-20-00195R2
**Full Title:**	The chromosome-level draft genome of a diploid plum (Prunus salicina)
**Article Type:**	Data Note
**Funding Information:**	The Industry University Research Collaborative Innovation Major Projects of Guangzhou Science Technology Innovation Commission (201704020021) — Dr Yehua He<br>Guangdong Key Laboratory of Innovation Method and Decision Management System (CN) (2016LM1128) — Dr Yehua He
**Abstract:**	Background: Plums are one of the most economically important Rosaceae fruit crops, and contain dozens of species distributed across the world. Until now, only limited genomic information is available for the genetic studies and breeding programs of plums. Prunus salicina , an important diploid plum species, plays a predominant role in modern commercial plums production. Here we selected P. salicina for whole-genome sequencing and presented a chromosome-level genome assembly through the combination of PacBio sequencing, Illumina sequencing and Hi-C technology.<br>Findings: The assembly had a total size of 284.2 Mb, with contig N50 of 1.8Mb and Scaffold N50 of 32.3 Mb. 96.56% of the assembled sequences were anchored onto eight pseudochromosomes and a total of 24,448 protein-coding genes were identified. Phylogenetic analysis showed that P. salicina had closer relationship with P. mume and P. armeniaca , with P. salicina diverging from their common ancestor approximately 9.05 million years ago (Mya). 146 gene families were expanded during P. salicina evolution, and some cell wall-related GO terms were significantly enriched. It was noteworthy that members in the DUF579 family, a new class involved in xylan biosynthesis, were significantly expanded in P. salicina, which provided new insight into the xylan metabolism in plums .<br>Conclusions: We constructed the first high-quality chromosome-level plum genome using PacBio, Illumina and Hi-C technologies. This work provides a valuable resource for facilitating plum breeding programs and studying the genetic diversity mechanisms of plums and Prunus species.
**Corresponding Author:**	Yehua He<br>Soth China Agricultural University<br>Guangzhou, Guangdong Province CHINA
**Corresponding Author Secondary Information:**	
**Corresponding Author's Institution:**	Soth China Agricultural University
**Corresponding Author's Secondary Institution:**	
**First Author:**	Chaoyang Liu
**First Author Secondary Information:**	
**Order of Authors:**	Chaoyang Liu<br>Chao Feng<br>Weizhuo Peng<br>Jingjing Hao<br>Juntao Wang<br>Jianjun Pan

Powered by Editorial Manager® and ProduXion Manager® from Aries Systems Corporation

	Yehua He
**Order of Authors Secondary Information:**	
**Response to Reviewers:**	Dear Editor and Reviewer,<br><br>We would like to thank you for helpful suggestions on our manuscript entitled "The chromosome-level draft genome of a diploid plum (Prunus salicina)" (GIGA-D-20-00195R1). Following the comments and suggestions, we corrected the numbering of the P. salicina pseudochromosomes all over the manuscript. The P. salicina pseudo-chromosome names and gene IDs in Figure 1, 3 and 4 were modified in the revised manuscript. We also contacted the staff of GDR (Genome Database for Rosaceae) and GenBank to update our genomic information. We expected that it would meet the publication requirement of GigaScience.<br><br>A point by point response to the reviewer' comments and questions and the main corrections in the paper were provided below.<br><br><br>Reviewer #1: I thank the authors for further revising their manuscript and clarifying some outstanding issues in regards of English proofreading and MS layout. Thank you very much for the answers to my previous questions, even if I do not fully agree with soem of them.<br>However, there is still a major revision necessary before the manuscript is ready for publication. I bet I overlooked it in the first version of the manuscript because of the other issues that were since corrected. My main concern relates to the chromosome nomenclature: the chromosome numbering is not in adequation with the Prunus genetic map. For exemple, Chromosome 1 in all Prunus species is always the largest one and following Figure 1, it appears that it is chromosome 2, here. The same remark applies to the other chromosomes, not only chromosome 2 (see figure 2B, chromosome 1 of P. salicina should in fact be chromosome 6 in the Prunus genetic map, chr3 should be chr4 and so on), and that's the reason why I was recommending using, even a few, Prunus genetic markers, to correct this discrepancy. This major issue is coming from the first release of the P. mume genome in 2012 and was reproduced in the P. armeniaca genome presented here. If colinearity has to be displayed (Figure 3) then it should be made clear that Chr2 here should be in fact Chr 1 in the genetic map. In fact, I would once again recommend the authors to re-order their chromosomes, according to the general acknowledged genetic map. Since the genetic maps were obtained by using molecular markers which are largely colinear and syntenic in between Prunus species (peach, P. mume, apricot and plum included) I would strongly recommend to right this issue, both within the P. salicina assembly and the following colinearity studies with the other genomes. Since genetic maps were released before genome assembly, the authors are expected to follow the internationally acknowledged nomenclature. Reproducing forever the mistake made initially for the P. mume genome would severely limit the interest of this de novo assembled genome and thus the impact of its release.<br>In conclusion, I recommend the authors to correct the numbering of the P. salicina chromosome all over the MS (by using a few of plum markers and even better Prunus orthologous markers as published in https://doi.org/10.1371/journal.pone.0208032, for that they only need to do a ePCR with markers depicted in Table S2F) and the data available online (and therefore Figure 3, accordingly).<br><br><br>Response：Thank you very much for your kindly suggestion. We further carefully read the literatures that you mentioned, and realized that the important role of the Prunus genetic maps was ignored in our first version of the manuscript. According to your kind advices, the numbering of the P. salicina pseudochromosome was corrected in the revised manuscript, using the markers that you recommended.<br><br>The present pseudochromosome numbering is consisted with that in the published P. salicina genetic map (Ref 77 in the revised manuscript). The P. salicina pseudo-chromosomes names and gene IDs in Figure 1, 3 and 4 were modified in the revised manuscript. The detailed information about the P. salicina pseudochromosomes in Table S5 was corrected accordingly. Moreover, we also contacted the staff of GDR (Genome Database for Rosaceae) and GenBank to update our genomic information.

Powered by Editorial Manager® and ProduXion Manager® from Aries Systems Corporation

Additional Information:	
Question	Response
Are you submitting this manuscript to a special series or article collection?	No
**Experimental design and statistics**<br><br>Full details of the experimental design and statistical methods used should be given in the Methods section, as detailed in our Minimum Standards Reporting Checklist. Information essential to interpreting the data presented should be made available in the figure legends.<br><br>Have you included all the information requested in your manuscript?	Yes
**Resources**<br><br>A description of all resources used, including antibodies, cell lines, animals and software tools, with enough information to allow them to be uniquely identified, should be included in the Methods section. Authors are strongly encouraged to cite Research Resource Identifiers (RRIDs) for antibodies, model organisms and tools, where possible.<br><br>Have you included the information requested as detailed in our Minimum Standards Reporting Checklist?	Yes
**Availability of data and materials**<br><br>All datasets and code on which the conclusions of the paper rely must be either included in your submission or deposited in publicly available repositories (where available and ethically appropriate), referencing such data using a unique identifier in the references and in the "Availability of Data and Materials" section of your manuscript.	Yes

Powered by Editorial Manager® and ProduXion Manager® from Aries Systems Corporation

Have you have met the above requirement as detailed in our Minimum Standards Reporting Checklist?	

Powered by Editorial Manager® and ProduXion Manager® from Aries Systems Corporation

Manuscript

Click here to access/download;Manuscript;Plum_for_production.docx

The chromosome-level draft genome of a diploid plum (*Prunus salicina*)

Chaoyang Liu[1, 3*], Chao Feng[2,*], Weizhuo Peng[1,3], Jingjing Hao[1,3], Juntao Wang[1,3]
Jianjun Pan[4], Yehua He[1,3]

[1] Key Laboratory of Biology and Germplasm Enhancement of Horticultural Crops in South China, Ministry of Agriculture, South China Agricultural University, Guangzhou 510642, China

[2] Key Laboratory of Plant Resources Conservation and Sustainable Utilization, South China Botanical Garden, Chinese Academy of Sciences, Guangzhou 510650, China

[3] Maoming Branch, Guangdong Laboratory for Lingnan Modern Agriculture, Maoming 525000, China

[4] Agricultural Technology Extension Center of Conghua District, Guangzhou. Guangzhou 510900, Guangdong Province, China

*Equal contribution

Corresponding author: Yehua He (email: heyehua@hotmail.com)

Abstract

Background: Plums are one of the most economically important Rosaceae fruit crops, and contain dozens of species distributed across the world. Until now, only limited genomic information is available for the genetic studies and breeding programs of plums. *Prunus salicina*, an important diploid plum species, plays a predominant role in modern commercial plums production. Here we selected *P. salicina* for whole-genome sequencing and presented a chromosome-level genome assembly through the combination of PacBio sequencing, Illumina sequencing and Hi-C technology. **Findings:** The assembly had a total size of 284.2 Mb, with contig N50 of 1.8Mb and scaffold N50 of 32.3Mb. 96.56% of the assembled sequences were anchored onto eight pseudochromosomes and a total of 24,448 protein-coding genes were identified. Phylogenetic analysis showed that *P. salicina* had closer relationship with *P. mume* and *P. armeniaca*, with *P. salicina* diverging from their common ancestor approximately 9.05 million years ago (Mya). 146 gene families were expanded during *P. salicina* evolution, and some cell wall-related GO terms were significantly enriched. It was noteworthy that members in the DUF579 family, a new class involved in xylan biosynthesis, were significantly expanded in *P. salicina,* which provided new insight into the xylan metabolism in plums. **Conclusions:** We constructed the first high-quality chromosome-level plum genome using PacBio, Illumina and Hi-C technologies. This work provides a valuable resource for facilitating plum breeding programs and studying the genetic diversity mechanisms of plums and *Prunus* species.

Background

Plums are one of the most economically important Rosaceae fruit crops and are produced throughout the world. About 12.6 million tons of plums (include sloes) are produced per year (FAOSTAT 2018, http://faostat.fao.org/), and the fruits are widely used for fresh consumption and processing like canning and beverages [1].There are 19-40 species of plums distributed across Asia, Europe and America. Plums have great diversity and are considered as a link between the major subgenera in the genus *Prunus* [2].

Prunus salicina, commonly called the Japanese plum or Chinese plum, is an important diploid (2x=2n=16) plum species that predominates in the modern commercial production of plums (Fig. 1). *P. salicina* originates in China and its fruits are mostly used for fresh consumption for their characteristic taste [3]. Cultivars of *P. salicina* have wide variability in phenology, fruit size and shape, flavour, firmness, aroma, texture, phenolic composition, antioxidant activity and both skin and pulp color [4].

However, the genetic and genomic information for *P. salicina* as well as most plum species were scarce [5]. The availability of a fully sequenced and annotated genome will help to measure and characterize the genetic diversity and determine how this diversity relates to the tremendous phenotypic diversity among plum cultivars. The genomic information is essential to support many of the studies involved in fundamental questions about plums biology and genetics. Moreover, genome-based tools could be developed to improve breeding works of plums, which were usually hindered by the high degree of heterozygosity, self-incompatible and long juvenile stage [2, 5, 6].

The fruit firmness, one of the most important indices of plum quality, is closely associated with cell wall compositions [2]. Xylan is a major component of secondary cell walls [7], and the xylan metabolism is involved in various aspects of plant growth and development like fruit ripening and softening [8]. According to previous studies, the plum species presented more xylose (the main component of xylan) compared to

other *Prunus* species, and plums were regarded as one of the richest natural sources of xyliot [9, 10]. The relatively high levels of xylan-related metabolites may be associated with the distinct mechanisms of the xylan metabolism in plums, and the available plum genomic information will be helpful to better understand the mechanism at molecular level.

Genome resources are already available for a number of Rosaceae fruit crops [11], including apple [12-15], peach [16], pear [17-20], strawberry [21, 22], almond [23, 24], black raspberry [25], sweet cherry [26, 27], apricot [28, 29], loquat [30] and *Prunus mume* [31]. However, whole-genome sequencing and chromosome-level assembly for plums have not been reported until now. In this study, *P. salicina* was selected for the whole-genome sequencing as a genomic reference. A high-quality chromosome-level *de novo* genome assembly of *P. salicina* was generated using an integrated strategy that combines PacBio sequencing, Illumina sequencing and Hi-C technology. The assembly has a total size of 284.2 Mb with contig N50 of 1.8Mb and scaffold N50 of 32.3 Mb, and vast majority (96.56%) of the assembled sequence was anchored onto eight pseudochromosomes. The availability of the high-quality chromosome-scale genome sequences not only provides fundamental knowledge regarding plum biology but also presents a valuable resource for genetic diversity analysis and breeding programs of plums and other *Prunus* crops.

Methods

Sample collection

The *Prunus salicina* Lindl. cv. ‘Sanyueli’, a Japanese plum landrace originating from Southern China, was selected for genome sequencing and assembly. ‘Sanyueli’ has a cultivation history of more than 200 years and many distinctive characteristics, including early-maturation, high-yield and low chilling requirements. The samples of the ‘Sanyueli’ were kept at the Horticultural Germplasm Conservation Center of South China Agricultural University (SCAU) for breeding and research in Guangzhou, Guangdong Province, China (113°22'4" N, 23°9'5" E). Total genomic DNA was

extracted from fresh young leaves of 5-year-old *P. salicina* tree using the CTAB method [32]. Samples from a total of six tissues, including leaf, flower, branch, young fruit pericarp, young fruit pulp and matured fruit, were collected from the same *P. salicina* tree. Total RNA was extracted from the six tissues using E.N.Z.A.® Plant RNA kit (OMEGA).

Library construction and sequencing

A combination of PacBio single-molecule real-time (SMRT) sequencing, Illumina's paired-end sequencing and Hi-C technology was applied. For PacBio sequencing, SMRT libraries were constructed using the PacBio 20-kb protocol (https://www.pacb.com/). The Illumina DNA paired-end libraries were constructed with an insert size of 350 bp, and sequencing was performed on the Illumina HiSeq 4000 platform according to the manufacturer's instructions. Reads with adaptors, with more than 10% unknown bases (N) and with more than 50% low-quality bases (⩽ 5) were filtered out to gain the clean data for further analysis.

The Hi-C library was prepared using the standard procedures. The young leaves of the same *P. salicina* tree were used as starting materials. Nuclear DNA from young leaves was cross-linked in situ, extracted, and digested with DpnII restriction endonuclease. The 5' overhangs of the digested fragments were biotinylated, and the resulting blunt ends were ligated. The cross-links were reversed after ligation, proteins were removed to release the DNA molecules. The purified DNA was sheared to a mean fragment size of 350 bp and ligated to adaptors, followed by purification through biotin-streptavidin-mediated pull down. The quality of Hi-C sequencing was evaluated with HiCUP [33].

The RNA-seq libraries for the six tissues of *P. salicina* were constructed according to the manufacturer's protocols, and were sequenced by Illumina Hiseq 4000 in paired-end 150bp mode.

Genome size estimation and *de novo* assembly

Sequencing data from the Illumina library were used to perform a k-mer analysis to

estimate the genome size of *P. salicina*. Quality-filtered reads were subjected to 17-mer frequency distribution analysis using SOAPdenovo (SOAPdenovo, RRID: SCR_010752) [34].

The *de novo* assembly of the *P. salicina* genome was carried out using the FALCON assembler (FALCON, RRID: SCR_016089) [35], followed by the polishing with Quiver [36] and Pilon (Pilon, RRID: SCR_014731) [37]. The PacBio subreads were subsequently processed by a self-correction of errors using FALCON [35] according to the manufacturer's instructions with the following parameters: length_cutoff =7,000, length_cutoff_pr = 4,000, max_diff = 100, max_cov = 100. The draft assembly was further polished using Quiver [36]. The pipeline of 'Purge Haplotigs' was used to remove the redundant sequences caused by genomic heterozygosity [38]. Finally, the Illumina reads were mapped back to the assembly and the remaining errors were corrected by Pilon [37].

Clean Hi-C reads were aligned to the assembled genome with BWA aligner (BWA, RRID: SCR 010910) with default parameters [39]. Only uniquely aligned read pairs whose mapping quality more than 20 were remained for further analysis. Invalid read pairs, including dangling-end and self-cycle, relegation, and dumped products, were filtered by HiCUP [33]. The valid interaction pairs were used to cluster, order, and orient the assembly contigs onto pseudochromosomes by LACHESIS (LACHESIS, RRID:SCR_017644； parameters: CLUSTER_N = 8, CLUSTER_MIN_RE_SITES =1157, CLUSTER_MAX_LINK_DENSITY = 5, CLUSTER _NONINFORMATIVE_RATIO = 0) [40]. The Juicebox [41] was applied to build the interaction matrices and complete the visual correction.

Genome quality evaluation

To evaluate the coverage of the assembly, the paired-end Illumina short reads were aligned to the assembly using BWA. RNA-seq reads from six tissues of *P. sacilina* were mapped against our assembly using Hisat with default parameters [42]. The SNPs were counted to evaluate the accuracy of the genome assembly. For CEGMA (Core Eukaryotic Genes Mapping Approach; CEGMA, RRID: SCR_015055)

evaluation, a set of highly reliable conserved protein families that occur in a range of model eukaryotes were build and then the 248 core eukaryotic genes were mapped to the genome [43]. Genome completeness was also accessed using BUSCO (Benchmarking Universal Single-Copy Orthologs; RRID: SCR_015008) analysis which included a set of 1440 single-copy orthologous genes [44].

Repeat annotations

To annotate repeat elements in the *P. salicina* genome, a combined strategy based on homology searching and *de novo* prediction was applied. For homology-based prediction, interspersed repeats were identified using RepeatMasker (http://www.repeatmasker.org) (RepeatMasker, RRID: SCR_012954) and RepeatProteinMask (RepeatProteinMask, RRID: SCR 012954) [45] to search against the Repbase database [46]. For *de novo* prediction, RepeatScout (http://www.repeatmasker.org/) (RepeatScout, RRID:SCR 014653) [47], RepeatModeler (http://www.repeatmasker.org/RepeatModeler/) RepeatModeler (RRID:SCR_015027), and LTR_Finder (http://tlife.fudan.edu.cn/tlife/ltr_finder/) (LTR_Finder, RRID:SCR_015247) [48] were used to identify *de novo* involved repeats. Tandem repeats were also *de novo* predicted using Tandem Repeats Finder (TRF) [49].

Telomere sequences were identified by BLASTN searches of both ends of the pseudochromosomes using four tandem repeats of the telomere repeat motif (TTTAGGG) with e-value cut-off of 0.003.

Gene annotations

A combination of three approaches, including homology-based prediction, *de novo* prediction and transcriptome-based prediction, was used to predict the protein-coding genes within *P. salicina* genome. For homology-based prediction, the homologous protein sequences of *Prunus persica*, *Prunus avium*, *Prunus mume*, *Pyrus bretschneideri*, *Malus domestica*, *Fragaria vesca* and *Arabidopsis thaliana* were obtained from NCBI database and mapped onto the *P. salicina* genome using TblastN

(TBLASTN; RRID:SCR_011822) (E-value ≤ 1e-5) [50], and then the matching proteins were aligned to the homologous genome sequences for accurate spliced alignments with GeneWise (GeneWise, RRID:SCR 015054) [51] to define gene models. For *de novo* prediction, Augustus (Augustus, RRID: SCR_008417) [52], GlimmerHMM (GlimmerHMM, RRID: SCR_002654) [53], SNAP (SNAP, RRID: SCR 002127) [54], GeneID (GeneID, RRID: SCR 002473) [55] and Genescan (Genescan, RRID: SCR_012902) [56] were used to predict the coding regions of genes. For transcriptome-based predictions, RNA-seq data from six tissues were used for genome annotation, processed by HISAT2 (HISAT2, RRID: SCR_015530) [42] and Stringtie (StringTie, RRID: SCR_016323) [57]. RNA-seq data were also *de novo* assembled with Trinity (Trinity, RRID: SCR_013048) [58]. The assembled sequences were aligned against *P. salicina* genome with PASA (Program to Assemble Spliced Alignment, PASA, RRID: SCR_014656) [59], and the effective alignments were assembled to gene structures. Gene models predicted by all of the methods were integrated by EVidenceModeler (EVidenceModeler, RRID: SCR_014659) [59]. To update the gene models, PASA was further used to generate UTRs [59].

Gene functions

The functional annotation of protein-coding genes within *P. salicina* genome was carried out by aligning protein sequences against SwissProt [60] and NR databases using BLASTp (with a threshold of E-value ≤ 1e-5). The protein motifs and domains were annotated by searching against InterPro (InterPro, RRID: SCR 006695) [61] and Pfam (Pfam, RRID: SCR_004726) database [62] with InterProScan (InterProScan, RRID: SCR_005829) [63]. Gene Ontology (GO) terms for each gene were retrieved according to the corresponding InterPro entry. KEGG pathway was mapped by the constructed gene set to identify the best match for each gene [64].

Non-coding RNA annotation

The tRNAs were predicted using the program tRNAscan-SE (tRNAscan-SE, RRID: SCR 010835) [65], and rRNA genes were annotated using BLASTN (BLASTN,

RRID: SCR_001598) tool with E-value of 1e-5 against rRNA sequences from several relative plant species. miRNA and snRNA were identified by searching against the Rfam (Rfam, RRID:SCR_007891) database [66] with default parameters using the INFERNAL software (INFERNAL, RRID:SCR 011809)[67].

Gene family construction

OrthoFinder version 2.3.3 (OrthoFinder, RRID:SCR_017118) [68] was used to classify the orthogroups of proteins from *P. salicina* and 16 other sequenced rosids species, including *P. armeniaca*, *P. mume*, *P. persica*, *P. dulcis*, *P. avium*, *P. yedoensis*, *M. domestica*, *P. bretschneideri*, *Pyrus communis*, *F. vesca*, *Potentilla micrantha*, *Rosa chinensis*, *Rosa multiflora*, *Rubus occidentalis*, *Morus notabilis* and *A. thaliana*.

Phylogenetic tree and divergence time estimation

For phylogenetic tree construction, proteins of single-copy orthogroups (i.e., the orthogroups which contain none or only one genes for each species) presented in at least 70% of species were selected and aligned with MAFFT version 6.846b (MAFFT, RRID: SCR 011811) [69]. After determination of the best substitution model for each orthogroup with IQ-TREE version 1.7-beta12 (IQ-TREE, RRID: SCR_017254) [70], the maximum likelihood phylogenetic tree across the 17 plant species was constructed using IQ-TREE with the parameter (-p -bb 1000), setting *A. thaliana* as outgroup.

The divergence time of each node in the phylogenetic tree was estimated with Bayesian Evolutionary Analysis Sampling Trees (BEAST, RRID: SCR_010228) [71].Two fossil constraints and a secondary calibration node were applied. The fossil *Prunus wutuensis* (age: Early Eocene, minimum age of 55.0 Mya) and the fossil *Rubus acutiformis* (age: Middle Eocene, minimum age of 41.3Mya) were placed at the stem *Prunus* and *Rubus*, respectively [72]. For the secondary calibration node, the divergence of Rosoideae and Amygdaloideae at 100.7 Mya was dated according to Xiang et al. [72]. The Markov chain Monte Carlo was reported 10,000,000 times with 1000 steps.

Gene family expansion and contraction analysis

For gene family expansion and contraction analysis, the ancestral gene content of each cluster at each node was investigated with CAFÉ version 3.1 (CAFÉ, RRID: SCR_005983) [73], basing on the phylogeny and gene numbers per orthogroup in each species, the gene family expansions/contractions at each branch were determined with *p*-value < 0.001.

Genome synteny analysis

A Python version of MCScan (minspan=100; MCScan, RRID: SCR_017650; https://github.com/tanghaibao/jcvi/wiki/MCscan) was employed to analyze the synteny between the *P. salicina* genome and other genomes within *Prunus* following the approaches of Haibao Tang [74].

Positively selected gene analysis

The ratios of nonsynonymous to synonymous substitutions (*Ka/Ks*) were calculated using the Codeml program with the free-ratio model as implemented in the PAML (PAML, RRID: SCR_014932) package [75]. The positive selection analysis was performed using the Codeml program with the optimized branch-site model as implemented in the PAML package. The positively selected genes were subjected to GO functional annotation.

Gene Ontology enrichment analysis

The Gene Ontology (GO) enrichment analysis for the specific groups of genes (e.g. tandem duplication and expanded genes) were performed using R package 'topGO' [76], setting all *P. salicina* genes as background. The lowest-level GO terms under enrichment (*p*-value < 0.01) were focused, and *p*-value was calculated using a 'classic' algorithm with the 'fisher' test. The lowest-level GO terms was based on the directed acyclic graph (DAG) of GO, with the parameter 'nodeSize = 100'.

The identification of the DUF579 family members

For the identification of the DUF579 family members, the hidden Markov model (HMM) profile corresponding to the DUF579 domain (PF04669) was downloaded from Pfam database (http://pfam.sanger.ac.uk/), and subsequently exploited for the genome of *P. salicina*, *P. persica*, *P. mume*, *P. armeniaca*, *P. dulcis* and *A. thaliana* using HMMER 3.0. The default parameters were employed and the cutoff value was set to 0.01.

Results and Discussion

Genome sequencing and assembly

We sequenced and assembled the genome of *P. salicina* using a combination of short-read sequencing from Illumina Hiseq, SMRT sequencing from PacBio and Hi-C technology. For the Illumina sequencing, a total of approximately 26.6 Gb (85.4 × coverage) short reads was obtained (Table S1). A total of ~53.0 Gb long-sequencing reads were generated by PacBio Sequel platform. After removing adaptors within sequences, about 52.9 Gb (169.7 × coverage) subreads were obtained (Table S1). The subreads have a mean length of 13.2 kb (Table S2). About 59.1 Gb (189.5 × coverage) sequencing data generated from Hi-C library was produced (Table S1). The quality of Hi-C sequencing was evaluated with HiCUP [33], and the effect rate was approximately 28.10% (Table S3).

In the genome assembly process, Illumina sequencing data were used for the genome survey and polishing of preliminary contigs, PacBio long reads were used for contig assembly and Hi-C reads were used for chromosome-level scaffolding. Based on the total number of k-mers (19,341,904,177), the estimated *P. salicina* genome size was calculated to be approximately 311.82 Mb (Figure S1). The heterozygous and repeat sequencing ratios were 0.70% and 54.49%, respectively (Table S4). The *de novo* genome assembly of *P. salicina with a* total length of 284.2 Mb (Table 1) was yielded. As shown in Fig. 1, the Hi-C assisted genome assembly was anchored onto the eight pseudochromosomes with lengths ranging from 23.70 to 54.53 Mb (Table S5), which were designated according to the published genetic map of *P. salicina* [77].

Five regions of tandemly repeated telomeric repeat sequences were identified on three pseudochromosomes (Table S5). The total length of pseudochromosomes accounted for 96.56% of the genome sequences (Figure 1), with contig N50 of 1.78 Mb and scaffold N50 of 32.32 Mb (Table1; Table S6).

Evaluation of the genome assembly

To assess the genome assembly quality, the Illumina clean data were aligned to the *P. salicina* genome, with the mapping rate of 96.93%. A total of 98.81% assembled genome was covered by the reads and the mapping coverage with at least 4×, 10×, 20× was 98.48 %, 98.06% and 97.13%, respectively (Table1; Table S7). The RNA-seq reads were mapped against the genome assembly, and the percentage of aligned reads ranged from 92.44% to 95.25% (Table1; Table S8). A total of 3,668 homozygous SNPs were identified, accounting for only 0.0015% of the reference genome (Table S9). The low rate of homozygous SNPs suggested that the assembly had a high base accuracy. 234 Core Eukaryotic Genes (CEGs) out of the complete set of 248 CEGs (94.35%) were covered by the assembly, and 229 (92.34%) of these were complete (Table1; Table S10). BUSCO analysis based on single copy orthologs set showed that 95.7% of the expected genes were identified as complete, 1.3% were fragmented, and only 3.0% were missing (Table1; Table S11). These results verified the high quality of the presently generated *P. salicina* genome assembly

Genome annotation

The results of the repeat annotations found that 48.28% of the assembly was covered with transposable elements (TE). Among them, long terminal repeat (LTR) retrotransposons represented the greatest proportion, making up 42.10% of the genome (Table1; Table S12). The TE percentage and density of duplicates resulted from tandem duplications were shown in Figure 1. Tandem duplicates occurred for 9.8% of the genes (Table 1) and were preferentially enriched in ‘transferase activity (GO: 0016758 and GO: 0016747)’ and ‘phloem development (GO: 0010088)’ (Figure

S2). The significant enrichment of the sieve element occlusion genes in ‘phloem development’, which were involved in wound sealing of the phloem [78], might be associated with specific requirements during the damage response in *P. salicina.*

For gene annotations, we predicted 24,448 non-redundant protein-coding genes in *P. salicina*. There were 24,209 genes (~99.0%) that could be assigned to eight pseudochromosomes (Table 1), and the gene density was shown in Figure 1. The average number of exons per gene, and average CDS length were 4.97 and 1,157.42, respectively (Table 2). Further gene functional annotation showed that 23,931 (97.9%) protein-coding genes were successfully annotated (Table 1; Table S13). For the identification of non-coding RNA (ncRNA) genes, a total of 627 miRNA, 960 tRNA, 273 rRNA and 2,023 snRNA in the *P. salicina* genome were predicted (Table S14).

Evolution of the *P. salicina* genome

The genome sequences of the representative sequenced rosid species were collected and subjected to comparative genomic analysis with *P. salicina* to reveal the genome evolution and divergence of *P. salicina.* A total of 15,751 orthogroups containing 23,265 genes were found in *P. salicina*. Moreover, 1,010 genes which were specific to *P. salicina* were identified. A comparison of the predicted proteomes among the 17 species indicated that 9,616, 10,447, 11,098, 13,963 and 15,512 orthogroups were shared between *P. salicina* and Rosids, Rosales, Rosaceae, Amygdaloideae and *Prunus,* respectively.

The phylogenetic analysis confirmed the close relationship among *P. salicina*, *P. mume* and *P. armeniaca*. The molecular clock of these plant genomes was also calculated. The data indicated that *P. salicina* diverged from the ancestor of *P. mume* and *P. armeniaca* approximately 9.05 Mya, from the ancestor of *P. persica* and *P.dulcis* 11.12 Mya (Figure 2).

We also explored the genome syntenic blocks between *P. salicina* and the other representative *Prunus* species. As shown in Fig. 3, our genome assembly of *P. salicina* exhibited a high level of genome synteny with all the other *Prunus* genomes, especially the genomes of *P. avium* and *P. dulcis*. Significantly fewer inversions were

found in *P. salicina* vs *P. avium* and *P. salicina* vs *P. dulcis* than that in *P. salicina* vs *P. mume* and *P. salicina* vs *P. armeniaca.*

Expansion and contraction of gene families in *P. salicina*

The gene family analysis showed that during the evolution of *P. salicina,* 146 gene families were expanded and 500 gene families were contracted. The functional enrichment on Gene Ontology of those expanded gene families identified 60 significantly enriched GO terms (p-value < 0.05) (Table S15; Figure S3).

It was noteworthy that genes from the expanded families were enriched in a series of cell wall related processes, such as 'cell wall polysaccharide metabolic process (GO: 0010383)', 'hemicellulose metabolic process (GO: 0010410)' and 'regulation of cellular biosynthetic process (GO: 0031326)'. Specially, genes in 'xylan biosynthetic process (GO: 0045492)', which corresponded to the DUF579 family [79], were significantly expanded. Further investigation showed that the major copy differences were found in Clade II, which consisted of orthologs of IRX15/IRX15L [79], with seven members in *P. salicina* and only two to four members in other *Prunus* species (Figure 4). It was reported that IRX15 and IRX15L defined a new class of genes involved in xylan biosynthesis [80, 81]. The species-specific expansion of this new subclade might contribute to the relatively high content of xylan-related metabolites (like xylose and xyliot) in plum [9, 10], which provided new insight into the xylan metabolism in plum.

Moreover, the FRS (FAR1-related sequence) gene family, which played multiple roles in a wide range of cellular processes [82], was also significantly expanded in the phylogeny (GO: 000945), and the family expansion may be related to the genetic and phenotypic diversity in *P. salicina*.

Positively selected genes in *P. salicina*

The Ka/Ks ratios for all the 2,314 single-copy orthologs shared with the sequenced *Prunus* species were calculated. A total of 213 candidate genes in *P. salicina* underwent positive selection ($P<0.05$). Most of them were enriched in the GO terms

involved in ‘monooxygenase activity (GO: 0004497)’ and ‘enzyme inhibitor activity (GO: 0004857)’ (Figure S4). It was noteworthy that the category ‘monooxygenase activity’ was also found in the enriched GO terms for the expanded gene families in *P. salicina*, which might provide valuable candidate genes for further functional investigations.

Conclusions

To our knowledge, this is the first report of the chromosome-level genome assembly of plums using Illumina and PacBio sequencing platforms with Hi-C technology. The assembly had a total size of 284.2 Mb, the contig and scaffold N50 reached 1.8 Mb and 32.3 Mb, respectively. A total of 24,448 protein-coding genes were predicted, and 23,931 genes (97.9%) have been annotated. Phylogenetic analysis indicated that *P. salicina* was closely related to *P. mume* and *P. armeniaca*. Expanded gene families in *P. salicina* were significantly enriched in several cell-wall related processes. Remarkably, the *P. salicina*-specific expansion of the xylan biosynthesis-related DUF579 family provided new insight into the xylan metabolism in plums. Given the economic and evolutionary importance of *P. salicina*, the genomic data in this study offer a valuable resource for facilitating plum breeding programs and studying the genetic basis for agronomic and adaptive divergence of plum and *Prunus* species.

Availability of supporting data and materials

This Whole Genome Shotgun project has been deposited at DDBJ/ENA/GenBank under the accession WERZ00000000. The version described in this paper is version WERZ01000000. The raw sequencing data are available through the NCBI Sequence Read Archive (SRA) via accession numbers from SRR10233497 to SRR10233505, via the Project PRJNA574159. The transcriptome data are available through the NCBI SRA (from SRR10235674 to SRR10235679). The genome data have also been submitted to *Genome Database for Rosaceae* (Accession number: tfGDR1044). All annotation tables containing results of an analysis of the draft genome are available at

Figshare [83]. Supporting data is also available via the *GigaScience* database GigaDB [84].

Additional files

Table S1 Statistics of *P. salicina* genome sequencing data.

Table S2 Statistics of characteristics of PacBio long-reads.

Table S3 Statistics of Hi-C sequencing data.

Table S4 Estimation of the genome size using k-mer analysis.

Table S5 Summary of assembled 8 pseudochromosomes of *P. Salicina.*

Table S6 Summary of the genome assembly of *P. Salicina.*

Table S7 Statistics of mapping ratio in genome.

Table S8 Summary of the transcriptome and their mapping rate on the genome assembly.

Table S9 Number and density of SNPs in *P. salicina* genome.

Table S10 Assessment of CEGMA.

Table S11 Summary of BUSCO analysis results according to prediction.

Table S12 Detailed classification of repeat sequences.

Table S13 Statistics of functional annotation.

Table S14 Summary of non-coding RNA.

Table S15 List of the Gene ontology terms significantly enriched in the expanded gene families of *P. salicina*

Figure S1 17-mer frequency distribution in *P. salicina* genome.

Figure S2 Gene ontology enrichment of the tandemly duplicated genes in *P. salicina*.

Figure S3 Gene ontology enrichment of *P. salicina*-expanded genes.

Figure S4 Gene ontology enrichment of the positively selected genes in *P. salicina*.

Authors' Contributions

Y.H.H. conceived the study. C.Y.L., C.F. and J.T.W. performed bioinformatics analysis. W.Z.P., J.J.H. and J.J.P. collected the samples and extracted the DNA. C.Y. L. and C. F. wrote the manuscript. All authors read and approved the final manuscript.

Abbreviations

BLAST: Basic Local Alignment Search Tool; BEAST: Bayesian Evolutionary Analysis Sampling Trees; bp: base pair; BUSCO: Benchmarking Universal Single-Copy Orthologs; CEGMA: Core Eukaryotic Genes Mapping Approach; CTAB: cetyltrimethylammonium bromide; EVM: EVidenceModeler; Gb: gigabase pair; GO: Gene Ontology; Hi-C: high-throughput chromosome conformation capture; kb: kilobase pair; KEGG: Kyoto Encyclopedia of Genes and Genomes; Mb: megabase pair; miRNA: microRNA; Mya: million years ago; NCBI: National Center for Biotechnology Information; PacBio: Pacific Biosciences; PAML: phylogenetic analysis by maximum likelihood; PASA: Program to Assemble Spliced Alignments; RNA-seq: RNA sequencing; rRNA: ribosomal RNA; SMRT: single-molecule real-time; SnRNA， small nuclear RNA; SNP: single-nucleotide polymorphism; TRF: Tandem Repeats Finder; tRNA: transfer RNA.

Funding

This work was financially supported by The Industry University Research Collaborative Innovation Major Projects of Guangzhou Science Technology Innovation Commission (201704020021) and Modern Agricultural Industry Technology System of Guangdong Province (2016LM1128).

Competing interests

The authors declare no competing interests.

Figure Legends

Figure 1 The genome and photograph of *P. salicina*. Landscape of the *P. salicina* genome, comprising 8 pseudochromosomes that cover ~96.56% of assembly (A); Concentric circles, from outermost to innermost, showing TE percentage (red; B); gene density (green; C); density of duplicates resulted from tandem duplications (blue; D); (E) photograph of *P. salicina*.

Figure 2 Evolution of *P. salicina* genome and orthogroups. (A) The phylogeny, divergence time and orthogroup expansions/contractions for 17 rosids species. The tree was constructed by maximum likelihood method using 341 single copy orthogroups. All nodes have 100% bootstrap support. Divergence time was estimated on a basis of three calibration points (blue circles). Blue bar indicates 95% HPD (highest posterior density) for each node. The numbers in red and green indicate the numbers of orthogroups that have expanded and contracted along particular branches, respectively. (B) The comparison of genes among 17 rosids. The grey bars indicate the genes belonging to 9,616 rosids-shared orthogroups in each of 17 rosids. The grey + green bars indicate the genes belonging to 10,447 rosales-shared orthogroups in each of 16 rosales. The grey + green + pink bars indicate the genes belonging to 11,098 Rosaceae-shared orthogroups in each of 15 Rosaceae. The grey + green + pink + yellow bars indicate the genes belonging to 13,963 rosaceae-shared orthogroups in each of ten Amygdaloideae. The grey + green + pink + yellow + blue bars indicate the genes belonging to 15,512 *Prunus*-shared orthogroups in each of seven *Prunus* species. The red and stripe bars indicate the genes in species-specific orthogroups and unassigned genes, respectively. The white bars indicate the remaining genes for each genome.

Figure 3 Chromosome-level collinearity patterns between *P. salicina*, *P. mume* and *P. armeniaca* (A) and between *P. salicina*, *P. avium* and *P. dulcis* (B). The numbers indicate the pseudochromosome order generated from the original genome sequence.

The pseudochromosome 2 and 6 in *P. armeniaca* and *P. mume* are reversed. Each gray line represents one block. The inverted regions are highlighted with brown color.

.

Figure 4 The significant expansion of the DUF579 family members in *P. salicina*. (A) Phylogenetic tree of the DUF579 proteins from *P. salicina* (red cicle), *P. persica* (hollow inverted triangle), *P. mume* (solid triangle), *P. armeniaca* (hollow diamond), *P. dulcis* (solid diamond) and *A. thaliana* (solid square). (B) The summary of the numbers of clade members in DUF579 family.

Table 1 Summary of genome assembly and annotation for *P. salicina*

	**Number or percentage**
**Assembly feature**	
Total length of scaffolds (bp)	284,209,110
Number of scaffolds	75
N50 of scaffolds (bp)	32,324,625
Total length of contigs (bp)	284,189,410
Number of contigs	272
N50 of contigs (bp)	1,777,944
Mapping rate by reads from short-insert libraries	96.93%
Assembled CEGs	94.35%
Completely assembled CEGs	92.34%
Complete BUSCOs	95.7%
Complete and single-copy BUSCOs	86.5%
Complete and duplicated BUSCOs	9.2%
Fragmented BUSCOs	1.3%
Missing BUSCOs	3.0%
RNA-Seq evaluation	92.44%-95.25%
**Genome annotation**	
Percentage of transposable elements (TE)	48.28%
Percentage of long terminal repeat (LTR) retrotransposon	42.1%
No. of predicted protein-coding genes	24,448
No. of genes assigned to pseudochromosomes	24,209 (99.0%)
No. of genes annotated to public database	23,930 (97.9%)
No. of genes annotated to GO database	13,484 (55.2%)
No. of genes duplicated by tandem duplications	2,384(9.8%)

Table 2 Statistics of predicted protein-coding genes.

Gene set		Number	Average transcript length (bp)	Average CDS length (bp)	Average exons per gene	Average exons length (bp)	Average intron length (bp)
De novo prediction	Augustus	23,592	2,627.71	1167.83	4.80	243.43	384.45
	GlimmerHMM	39,985	5,450.51	747.07	3.14	238.12	2200.59
	SNAP	24,882	2,876.50	728.45	4.22	172.73	667.66
	Geneid	33,780	3,829.40	899.99	4.44	202.74	851.78
	Genscan	21,882	8,251.09	1355.87	6.34	213.98	1292.13
Homolog prediction	*Pyrus bretschneideri*	20,265	3,119.83	1356.17	4.74	286.35	472.06
	Malus domestica	20,010	2,920.17	1361.30	4.65	292.56	426.72
	Prunus mume	23,064	3,038.66	1346.19	4.78	281.67	447.84
	Prunus persica	28,915	2,296.51	1099.56	4.06	270.55	390.64
	Arabidopsis thaliana	28,284	2,071.73	973.28	3.67	265.51	412.07
	Fragaria vesca	22,927	2,994.24	1380.61	4.59	300.66	449.24
	Prunus avium	22,715	3,077.20	1351.28	4.74	284.86	461.03
RNA-seq	PASA	196,264	3,913.86	1008.68	5.16	195.60	698.88
	Transcripts	42,450	11,076.28	2360.92	6.85	344.83	1490.64
EVM		27,981	2,736.70	1061.73	4.57	232.52	469.68
PASA-update*		27,594	2,784.15	1092.82	4.64	235.59	464.83
Final set*		24,448	2,988.45	1157.42	4.97	233.09	461.72

* UTR regions were contained

References

1. Roussos PA, Efstathios N, Intidhar B, Denaxa N-K and Tsafouros A. Plum (*Prunus domestica* L. and *P. salicina* Lindl.). In: Monique Simmonds VRP, editor. Nutritional Composition of Fruit Cultivars. Elsevier; 2016. p. 639 - 666.
2. Topp BL, Russell DM, Neumüller M, Dalbó MA and Liu W. Plum. In: Maria Luisa Badenes DHB, editor. Fruit Breeding. Springer; 2012. p. 571-621.
3. Hartmann W and Neumüller M. Plum breeding. In: Shri Mohan Jain PMP, editor. Breeding Plantation Tree Crops: Temperate Species. Springer; 2009. p. 161-231.
4. Okie W and Hancock J. Plums. In: Hancock JF, editor. Temperate Fruit Crop Breeding. Springer Science & Business Media; 2008. p. 337-358.
5. Esmenjaud D and Dirlewanger E. Plum. In: Kole C, editor. Genome Mapping and Molecular Breeding in Plants. Springer; 2007. p. 119-135.
6. Guerra M and Rodrigo J. Japanese plum pollination: A review. SCI Hortic-Amsterdam 2015;**197**:674-686.
7. Rennie EA and Scheller HV. Xylan biosynthesis. Curr Opin Biotech 2014;**26**:100-107.
8. Brummell DA and Schröder R. Xylan metabolism in primary cell walls. NZ J Forestry Sci. 2009;**39**:125-143.
9. Renard CMGC and Ginies C. Comparison of the cell wall composition for flesh and skin from five different plums. Food Chem 2009;**114**(3):1042-1049.
10. Arcaño YD, García ODV, Mandelli D, Carvalho WA and Pontes LAM. Xylitol: A review on the progress and challenges of its production by chemical route. Catal Today 2020;**344**:2-14.
11. Aranzana MJ, Decroocq V, Dirlewanger E, Eduardo I, Gao ZS, Gasic K, et al. *Prunus* genetics and applications after *de novo* genome sequencing: achievements and prospects. Hortic Res 2019;**6** (1):1-25.
12. Velasco R, Zharkikh A, Affourtit J, Dhingra A, Cestaro A, Kalyanaraman A, et al. The genome of the domesticated apple (*Malus*× *domestica* Borkh.). Nat Genet 2010;**42** (10):833-839.
13. Chen X, Li S, Zhang D, Han M, Jin X, Zhao C, et al. Sequencing of a wild apple (*Malus baccata*) genome unravels the differences between cultivated and wild apple species regarding disease resistance and cold tolerance. G3: Genes, Genomes, Genet 2019;**9** (7):2051-2060.
14. Zhang L, Hu J, Han X, Li J, Gao Y, Richards CM, et al. A high-quality apple genome assembly reveals the association of a retrotransposon and red fruit colour. Nat Commun 2019;**10** (1):1-13.
16. Verde I, Abbott AG, Scalabrin S, Jung S, Shu S, Marroni F, et al. The high-quality draft genome of peach (*Prunus persica*) identifies unique patterns of genetic diversity, domestication and genome evolution. Nat Genet 2013;**45**(5):487-494.
17. Linsmith G, Rombauts S, Montanari S, Deng CH, Celton J-M, Guérif P, et al.

Pseudo-chromosome–length genome assembly of a double haploid “Bartlett” pear (*Pyrus communis* L.) GigaSciemce 2019; 8 (12):giz138.

18. Wu J, Wang Z, Shi Z, Zhang S, Ming R, Zhu S, et al. The genome of the pear (*Pyrus bretschneideri* Rehd.). Genome Res 2013;**23**(2):396-408.
19. Chagné D, Crowhurst RN, Pindo M, Thrimawithana A, Deng C, Ireland H, et al. The draft genome sequence of European pear (*Pyrus communis* L.‘Bartlett’). PloS One 2014;**9** (4):e92644.
20. Dong X, Wang Z, Tian L, Zhang Y, Qi D, Huo H, et al. *De novo* assembly of a wild pear (*Pyrus betuleafolia*) genome. Plant Biotechnol J 2020;**18**(2):581-595
21. Shulaev V, Sargent DJ, Crowhurst RN, Mockler TC, Folkerts O, Delcher AL, et al. The genome of woodland strawberry (*Fragaria vesca*). Nat Genet 2011;**43**(2):109-116.
22. Edger PP, Poorten TJ, VanBuren R, Hardigan MA, Colle M, McKain MR, et al. Origin and evolution of the octoploid strawberry genome. Nat Genet 2019;**51**(3):541-547.
23. Alioto T, Alexiou KG, Bardil A, Barteri F, Castanera R, Cruz F, et al. Transposons played a major role in the diversification between the closely related almond and peach genomes: Results from the almond genome sequence. Plant J 2020;**101**(2):455-472.
24. Sánchez-Pérez R, Pavan S, Mazzeo R, Moldovan C, Cigliano RA, Del Cueto J, et al. Mutation of a bHLH transcription factor allowed almond domestication. Science 2019; **364** (6445):1095-1098.
25. VanBuren R, Bryant D, Bushakra JM, Vining KJ, Edger PP, Rowley ER, et al. The genome of black raspberry (*Rubus occidentalis*). Plant J 2016;**87**(6):535-547.
26. Shirasawa K, Isuzugawa K, Ikenaga M, Saito Y, Yamamoto T, Hirakawa H, et al. The genome sequence of sweet cherry (*Prunus avium*) for use in genomics-assisted breeding. DNA Res 2017;**24**(5):499-508.
27. Wang J, Liu W, Zhu D, Hong P, Zhang S, Xiao S, et al. Chromosome-scale genome assembly of sweet cherry *(Prunus avium* L.) cv. Tieton obtained using long-read and Hi-C sequencing. Hort Res 2020;**7** (1):1-11.
28. Jiang F, Zhang J, Wang S, Yang L, Luo Y, Gao S, et al. The apricot (*Prunus armeniaca* L.) genome elucidates Rosaceae evolution and beta-carotenoid synthesis. Hortic Res 2019;6 (1):1-12.
29. Campoy JA, Sun H, Goel M, Jiao W-B, Folz-Donahue K, Kukat C, et al. Chromosome-level and haplotype-resolved genome assembly enabled by high-throughput single-cell sequencing of gamete genomes. BioRxiv. 2020.
30. Jiang S, An H, Xu F and Zhang X. Chromosome-level genome assembly and annotation of the loquat (*Eriobotrya japonica*) genome. GigaScience 2020;**9**(3):giaa015.
31. Zhang Q, Chen W, Sun L, Zhao F, Huang B, Yang W, et al. The genome of *Prunus mume*. Nat Commun 2012;**3**:1318.
32. Lodhi MA, Ye G-N, Weeden NF and Reisch BI. A simple and efficient method for DNA extraction from grapevine cultivars and *Vitis* species. Plant Mol Biol Rep. 1994;**12** (1):6-13.

33. Wingett S, Ewels P, Furlan-Magaril M, Nagano T, Schoenfelder S, Fraser P, et al. HiCUP: pipeline for mapping and processing Hi-C data. F1000Res 2015;**4**:1310.
34. Luo R, Liu B, Xie Y, Li Z, Huang W, Yuan J, et al. SOAPdenovo2: an empirically improved memory-efficient short-read *de novo* assembler. Gigascience 2012;**1** (1):2047-217X-1-18.
35. Chin C-S, Peluso P, Sedlazeck FJ, Nattestad M, Concepcion GT, Clum A, et al. Phased diploid genome assembly with single-molecule real-time sequencing. Nat Methods 2016;**13** (12):1050-1054.
36. Chin C-S, Alexander DH, Marks P, Klammer AA, Drake J, Heiner C, et al. Nonhybrid, finished microbial genome assemblies from long-read SMRT sequencing data. Nat Methods 2013;**10**(6):563-569.
37. Walker BJ, Abeel T, Shea T, Priest M, Abouelliel A, Sakthikumar S, et al. Pilon: an integrated tool for comprehensive microbial variant detection and genome assembly improvement. PloS One 2014;**9** (11):e112963.
38. Roach MJ, Schmidt SA and Borneman ARJBb. Purge Haplotigs: allelic contig reassignment for third-gen diploid genome assemblies. BMC Bioinformatics 2018;**19** (1):460.
39. Li H and Durbin R. Fast and accurate short read alignment with Burrows-Wheeler transform. Bioinformatics 2009;**25** (14):1754-1760.
40. Burton JN, Adey A, Patwardhan RP, Qiu R, Kitzman JO and Shendure J. Chromosome-scale scaffolding of *de novo* genome assemblies based on chromatin interactions. Nat Biotechnol 2013;**31** (12):1119-1125.
41. Robinson JT, Turner D, Durand NC, Thorvaldsdottir H, Mesirov JP and Aiden EL. Juicebox. js provides a cloud-based visualization system for Hi-C data. Cell Syst 2018;**6**(2):256-258. e1.
42. Kim D, Langmead B and Salzberg SL. HISAT: a fast spliced aligner with low memory requirements. Nat Methods 2015;**12** (4):357-360.
43. Parra G, Bradnam K and Korf I. CEGMA: a pipeline to accurately annotate core genes in eukaryotic genomes. Bioinformatics 2007;**23** (9):1061-1067.
44. Simão FA, Waterhouse RM, Ioannidis P, Kriventseva EV and Zdobnov EM. BUSCO: assessing genome assembly and annotation completeness with single-copy orthologs. Bioinformatics 2015;**31**(19):3210-3212.
45. Tarailo‐Graovac M and Chen N. Using RepeatMasker to identify repetitive elements in genomic sequences. Curr Protoc Bioinf 2009;**25** (1):4-10.
46. Jurka J, Kapitonov VV, Pavlicek A, Klonowski P, Kohany O and Walichiewicz J. Repbase Update, a database of eukaryotic repetitive elements. Cytogenet Genome Res 2005;**110** (1-4):462-467.
47. Price AL, Jones NC and Pevzner PA. *De novo* identification of repeat families in large genomes. Bioinformatics 2005;**21** (suppl_1):i351-i358.
48. Xu Z and Wang H. LTR_FINDER: an efficient tool for the prediction of full-length LTR retrotransposons. Nucleic Acids Res 2007;**35** (suppl_2):W265-W268.

49. Benson G. Tandem repeats finder: a program to analyze DNA sequences. Nucleic Acids Res 1999;**27** (2):573-580.

50. Gertz EM, Yu Y-K, Agarwala R, Schäffer AA and Altschul SF. Composition-based statistics and translated nucleotide searches: improving the TBLASTN module of BLAST. BMC Biol 2006;**4** (1):1-14.

51. Birney E, Clamp M and Durbin R. GeneWise and genomewise. Genome Res 2004;**14** (5):988-995.

52. Stanke M, Steinkamp R, Waack S and Morgenstern B. AUGUSTUS: a web server for gene finding in eukaryotes. Nucleic Acids Res 2004;**32** (suppl_2):W309-W312.

53. Majoros WH, Pertea M and Salzberg SL. TigrScan and GlimmerHMM: two open source ab initio eukaryotic gene-finders. Bioinformatics 2004;**20** (16):2878-2879.

54. Korf I. Gene finding in novel genomes. BMC Bioinf 2004;**5** (1):59.

55. Blanco E, Parra G and Guigó R. Using geneid to identify genes. Curr Protoc Bioinf 2007;**18** (1):4.3. 1-4.3. 28.

56. Burge C and Karlin S. Prediction of complete gene structures in human genomic DNA. J Mol Biol 1997;**268** (1):78-94.

57. Pertea M, Pertea GM, Antonescu CM, Chang T-C, Mendell JT and Salzberg SL. StringTie enables improved reconstruction of a transcriptome from RNA-seq reads. Nat Biotechnol 2015;**33**(3):290-295.

58. Haas BJ, Papanicolaou A, Yassour M, Grabherr M, Blood PD, Bowden J, et al. *De novo* transcript sequence reconstruction from RNA-seq using the Trinity platform for reference generation and analysis. Nat Protoc 2013;**8** (8):1494-1512.

59. Haas BJ, Salzberg SL, Zhu W, Pertea M, Allen JE, Orvis J, et al. Automated eukaryotic gene structure annotation using EVidenceModeler and the Program to Assemble Spliced Alignments. Genome Biol 2008;**9** (1):R7.

60. Bairoch A and Apweiler R. The SWISS-PROT protein sequence database and its supplement TrEMBL in 2000. Nucleic Acids Res 2000;**28** (1):45-48.

61. Mulder N and Apweiler R. InterPro and InterProScan: tools for protein sequence classifcation and comparison. Methods Mol Biol 2007; **396**:59-70.

62. Finn RD, Bateman A, Clements J, Coggill P, Eberhardt RY, Eddy SR, et al. Pfam: the protein families database. Nucleic Acids Res 2013;**42** (D1):D222-D230.

63. Jones P, Binns D, Chang H-Y, Fraser M, Li W, McAnulla C, et al. InterProScan 5: genome-scale protein function classification. Bioinformatics 2014;**30** (9):1236-1240.

64. Kanehisa M and Goto S. KEGG: kyoto encyclopedia of genes and genomes. Nucleic Acids Res 2000;**28** (1):27-30.

65. Lowe TM and Eddy SR. tRNAscan-SE: a program for improved detection of transfer RNA genes in genomic sequence. Nucleic Acids Res 1997;**25** (5):955-964.

66. Griffiths-Jones S, Bateman A, Marshall M, Khanna A and Eddy SR. Rfam: an RNA family database. Nucleic Acids Res 2003;**31**(1):439-441.

67. Nawrocki EP and Eddy SR. Infernal 1.1: 100-fold faster RNA homology searches. Bioinformatics 2013;**29** (22):2933-2935.
68. Emms DM and Kelly S. OrthoFinder: solving fundamental biases in whole genome comparisons dramatically improves orthogroup inference accuracy. Genome Biol 2015;**16** (1):157.
69. Katoh K and Standley DM. MAFFT multiple sequence alignment software version 7: improvements in performance and usability. Mol Biol Evol 2013;**30** (4):772-780.
70. Nguyen L-T, Schmidt HA, Von Haeseler A and Minh BQ. IQ-TREE: a fast and effective stochastic algorithm for estimating maximum-likelihood phylogenies. Mol Biol Evol 2015;**32** (1):268-274.
71. Drummond AJ and Rambaut A. BEAST: Bayesian evolutionary analysis by sampling trees. BMC Evol Biol 2007;**7** (1):1-8.
72. Xiang Y, Huang C-H, Hu Y, Wen J, Li S, Yi T, et al. Evolution of Rosaceae fruit types based on nuclear phylogeny in the context of geological times and genome duplication. Mol Biol Evol 2017;**34** (2):262-281.
73. De Bie T, Cristianini N, Demuth JP and Hahn MW. CAFE: a computational tool for the study of gene family evolution. Bioinformatics 2006;**22** (10):1269-1271.
74. Tang H. Multiple collinearity scan—mcscan. 2009.
75. Yang Z. PAML 4: phylogenetic analysis by maximum likelihood. Mol Biol Evol 2007;**24**(8):1586-1591.
76. Alexa A and Rahnenführer J. Gene set enrichment analysis with topGO. Bioconductor Improv. 2009;27.
77. Carrasco B, González M, Gebauer M, García-González R, Maldonado J and Silva HJPo. Construction of a highly saturated linkage map in Japanese plum (*Prunus salicina* L.) using GBS for SNP marker calling. PloS one 2018;**13** (12): e0208032.
78. Ernst AM, Jekat SB, Zielonka S, Müller B, Neumann U, Rüping B, et al. Sieve element occlusion (SEO) genes encode structural phloem proteins involved in wound sealing of the phloem. P Natl Acad Sci USA 2012;**109** (28): E1980-E1989.
79. Temple H, Mortimer JC, Tryfona T, Yu X, Lopez‐Hernandez F, Sorieul M, et al. Two members of the DUF 579 family are responsible for arabinogalactan methylation in Arabidopsis. Plant Direct 2019;**3** (2):e00117.
80. Jensen JK, Kim H, Cocuron JC, Orler R, Ralph J and Wilkerson CG. The DUF579 domain containing proteins IRX15 and IRX15-L affect xylan synthesis in Arabidopsis. Plant J 2011;**66** (3):387-400.
82. Brown D, Wightman R, Zhang Z, Gomez LD, Atanassov I, Bukowski JP, et al. Arabidopsis genes IRREGULAR XYLEM (IRX15) and IRX15L encode DUF579‐containing proteins that are essential for normal xylan deposition in the secondary cell wall. Plant J 2011;**66** (3):401-413.
82. Ma L and Li G. FAR1-related sequence (FRS) and FRS-related factor (FRF) family proteins

in *Arabidopsis* growth and development. Front Plant Sci 2018; **9**:692.

83. Liu C, Feng C, Peng W, Hao J, Pan J, He Y. Annotation results of Prunus salicina genome Figshare 2020. https://doi.org/10.6084/m9.figshare.9973469.

84 Liu C, Feng C, Peng W, Hao J, Wang J, Pan J, He Y Supporting data for "The chromosome-level draft genome of a diploid plum (Prunus salicina)" GigaScience Database. 2020. http://dx.doi.org/10.5524/100811

Figure 1

Click here to access/download;Figure;Figure 1-9-28.tif

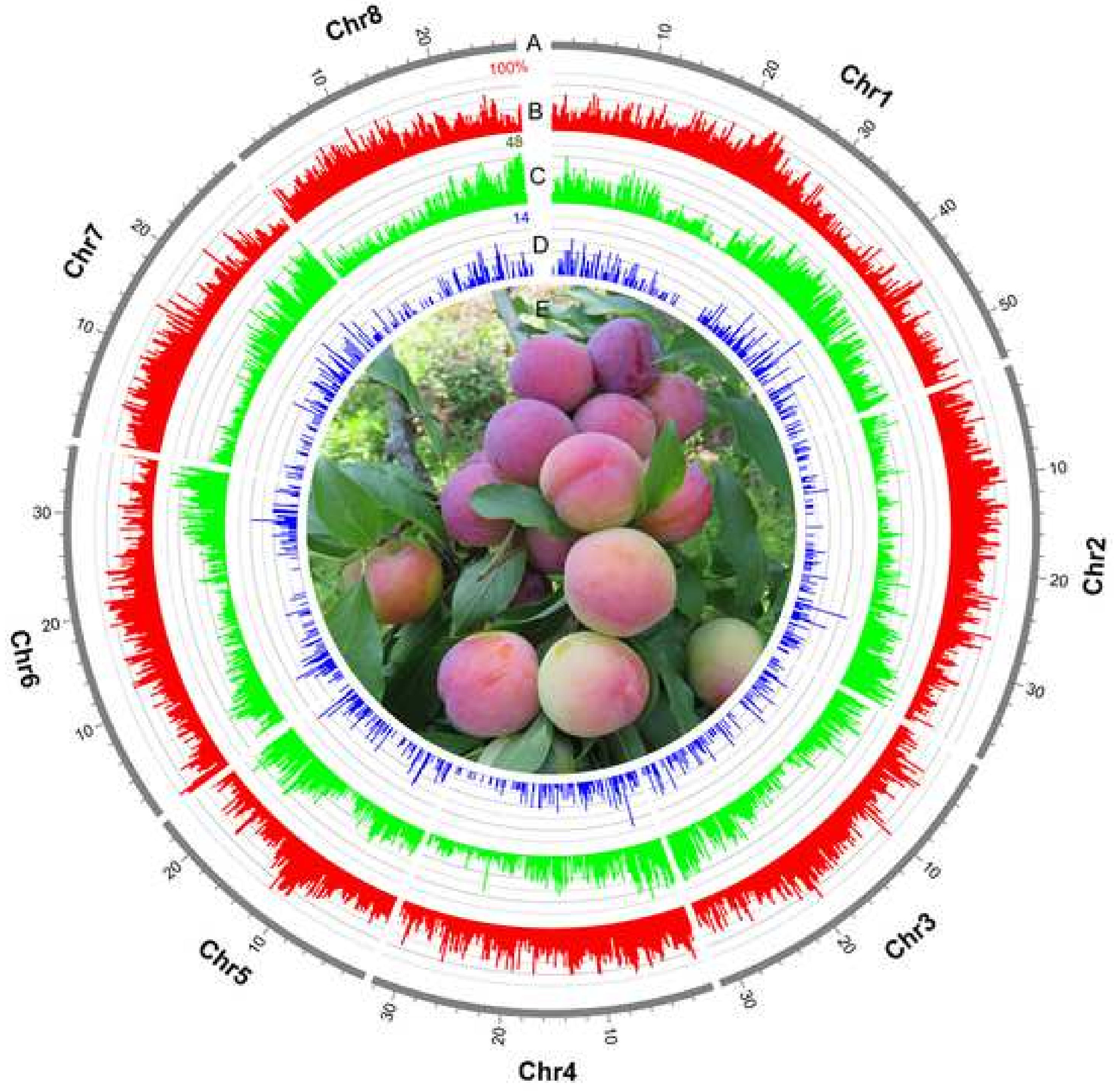

Figure 2

Click here to access/download;Figure;Figure 2-ok.tif

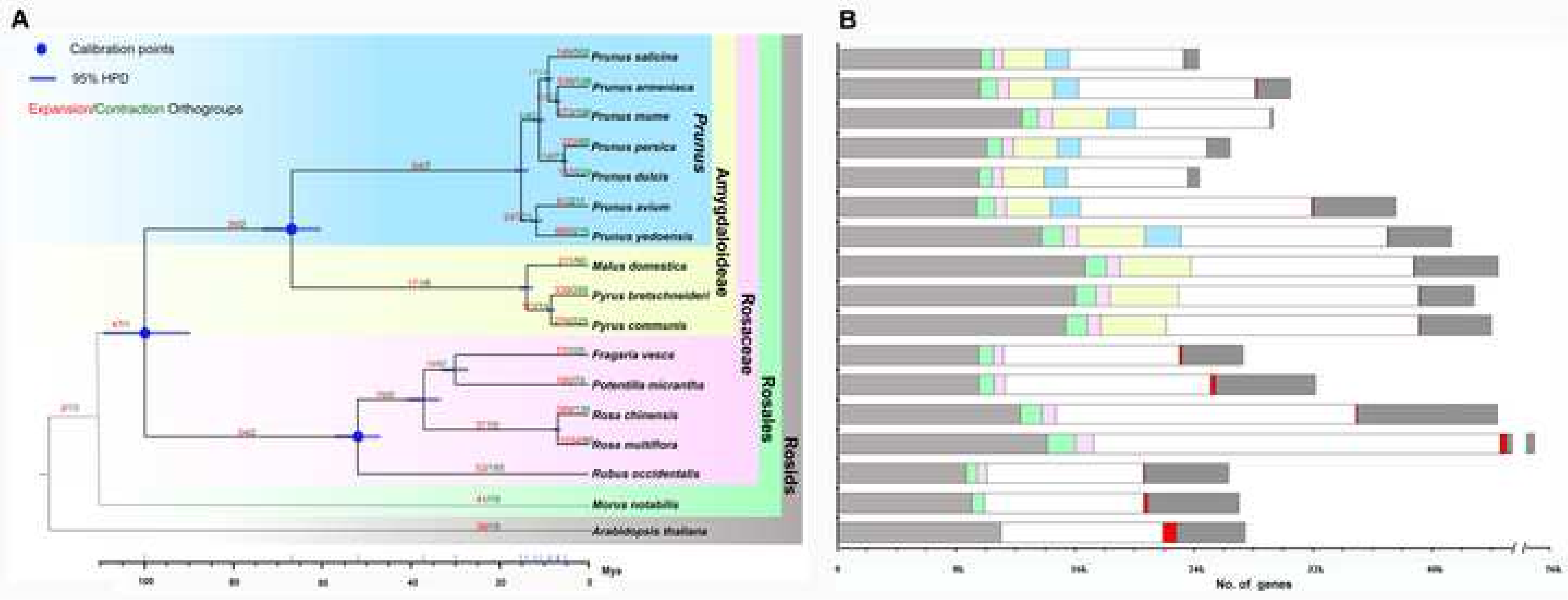

Figure 3

Click here to access/download;Figure;Figure 3-9-28.tif

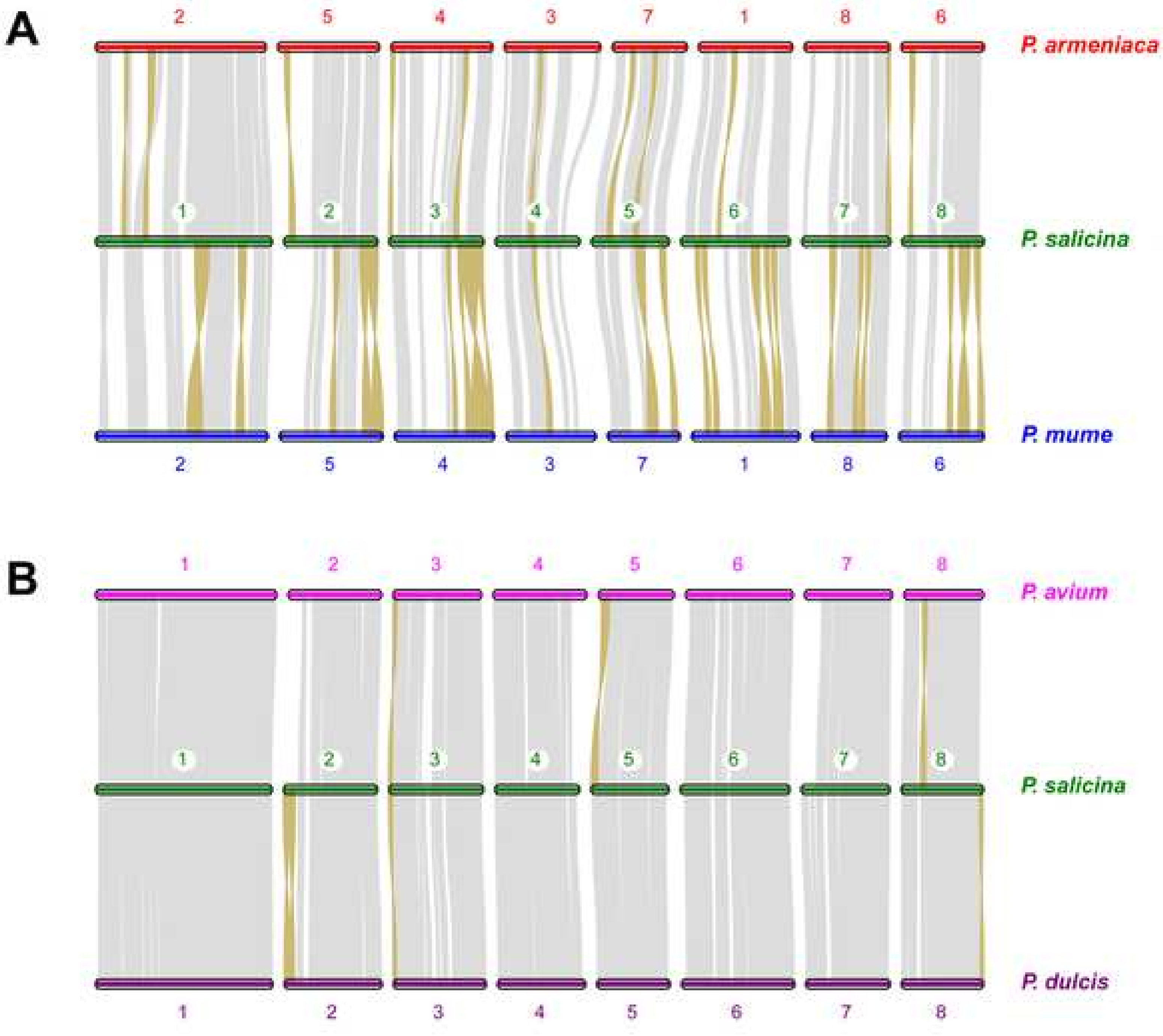

Figure 4 Click here to access/download;Figure;Figure 4-9-28.tif

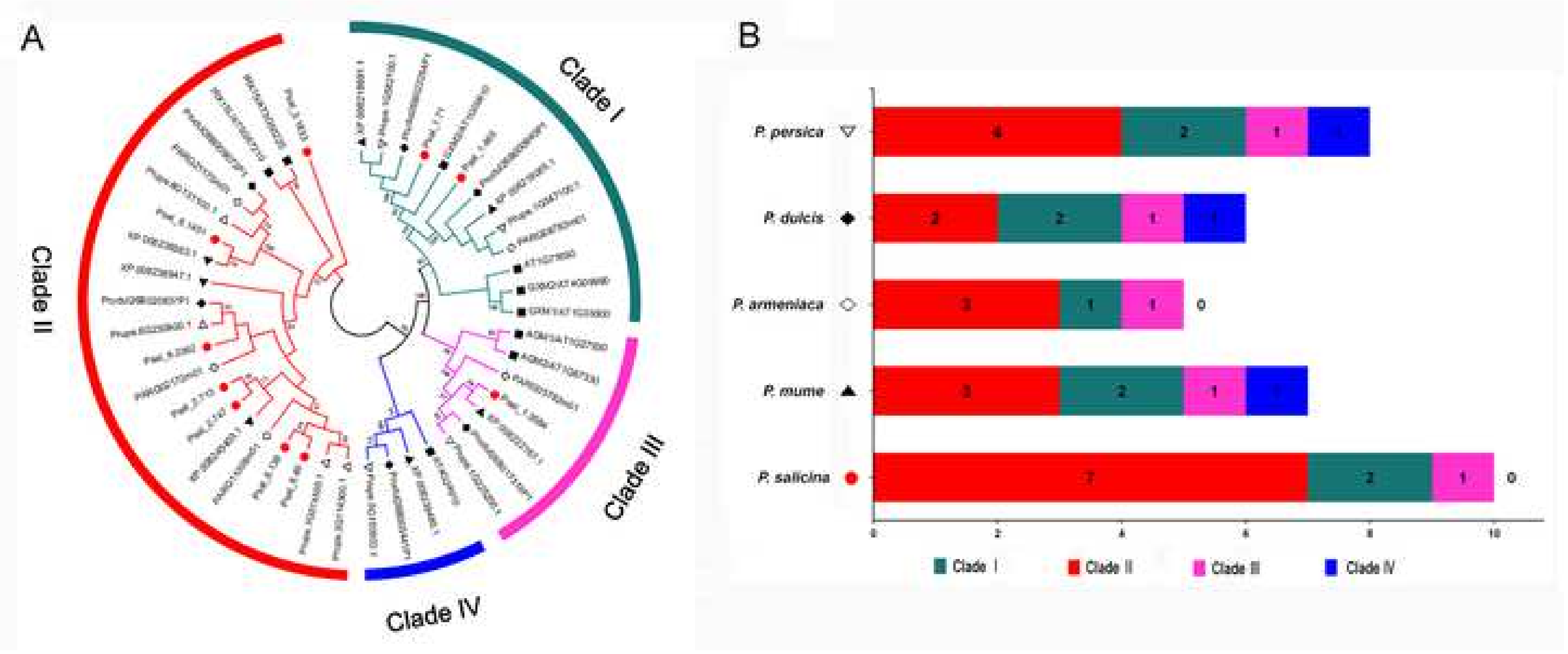

Click here to access/download
Supplementary Material
Supplementary Figures.pdf

Supplementary Tables

Click here to access/download
Supplementary Material
Supplementary Tables-9-28.xlsx
