# Supplementary material for: Chromosome-level draft genome of a diploid plum (Prunus salicina)
Source: Gigascience. 2020 Dec 10;9(12):giaa130. doi: 10.1093/gigascience/giaa130 (PMC7727024; doi:10.1093/gigascience/giaa130)
Supplement: giaa130_Supplemental_Files [file giaa130_supplemental_files.zip › Supplementary Figures.pdf]

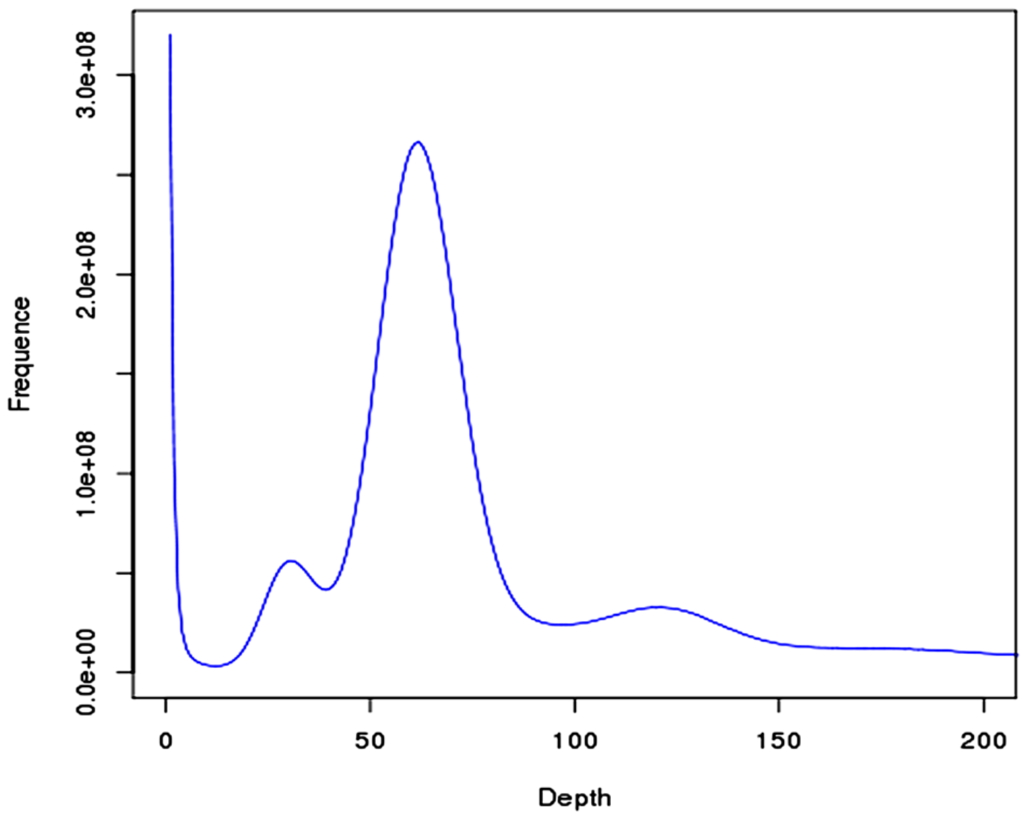

Figure S1 17-mer frequency distribution in *P. salicina* genome.

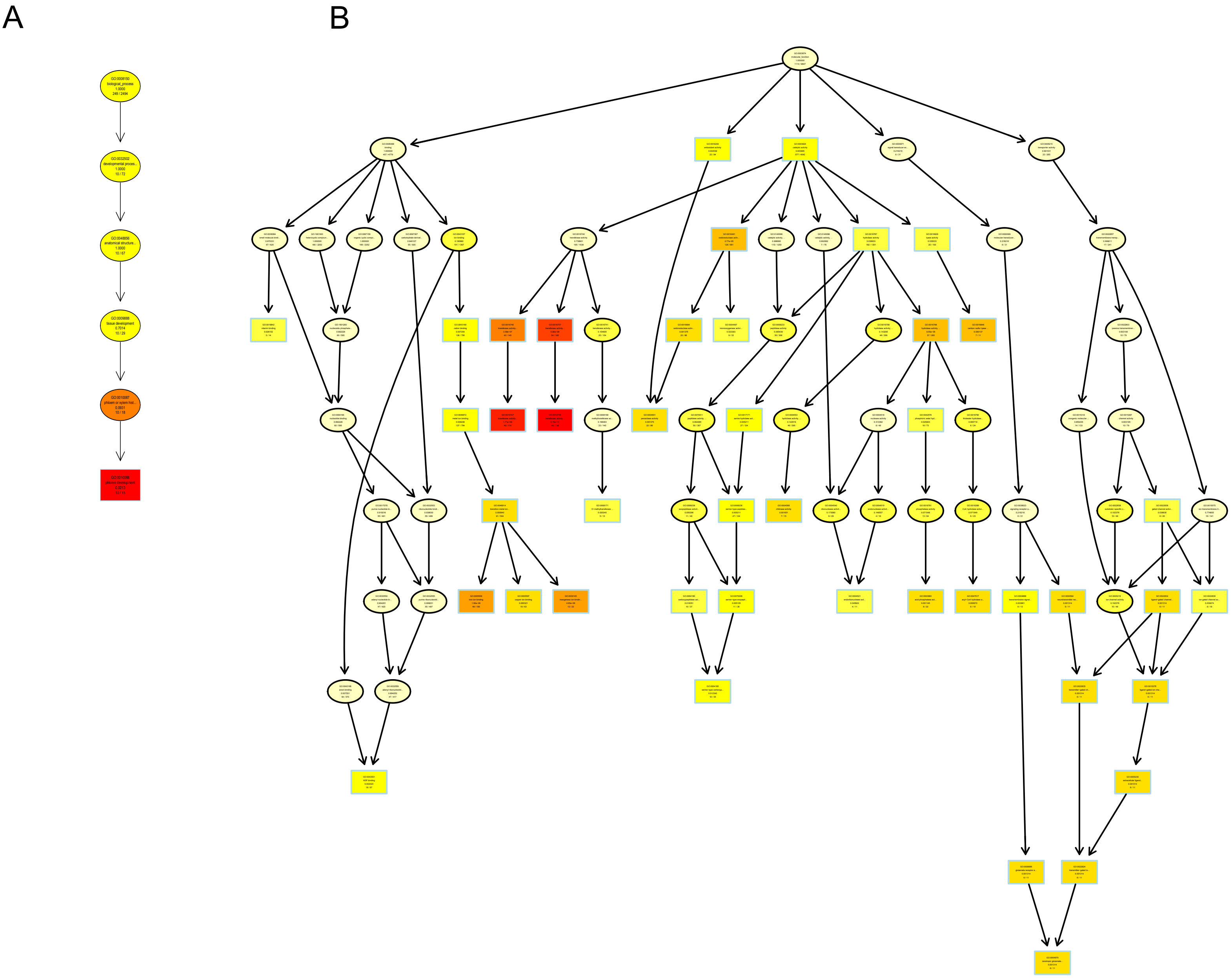

Figure S2 Gene ontology enrichment of the tandemly duplicated genes in *P. salicina*. Directed acyclic graph showed top enriched GO terms belonging to Category Biological Process (A) and Molecular Function (B). Rectangles indicate the significant terms with p-value < 0.01, with color ranging from dark red (represent most significant p-value) to bright yellow (least significant).The information displayed for each node, from first line to last line, is the GO term, GO name, p-value, and the number of all syntenic duplicates/ the number of total genes annotated to the respective GO term, respectively.

A

B

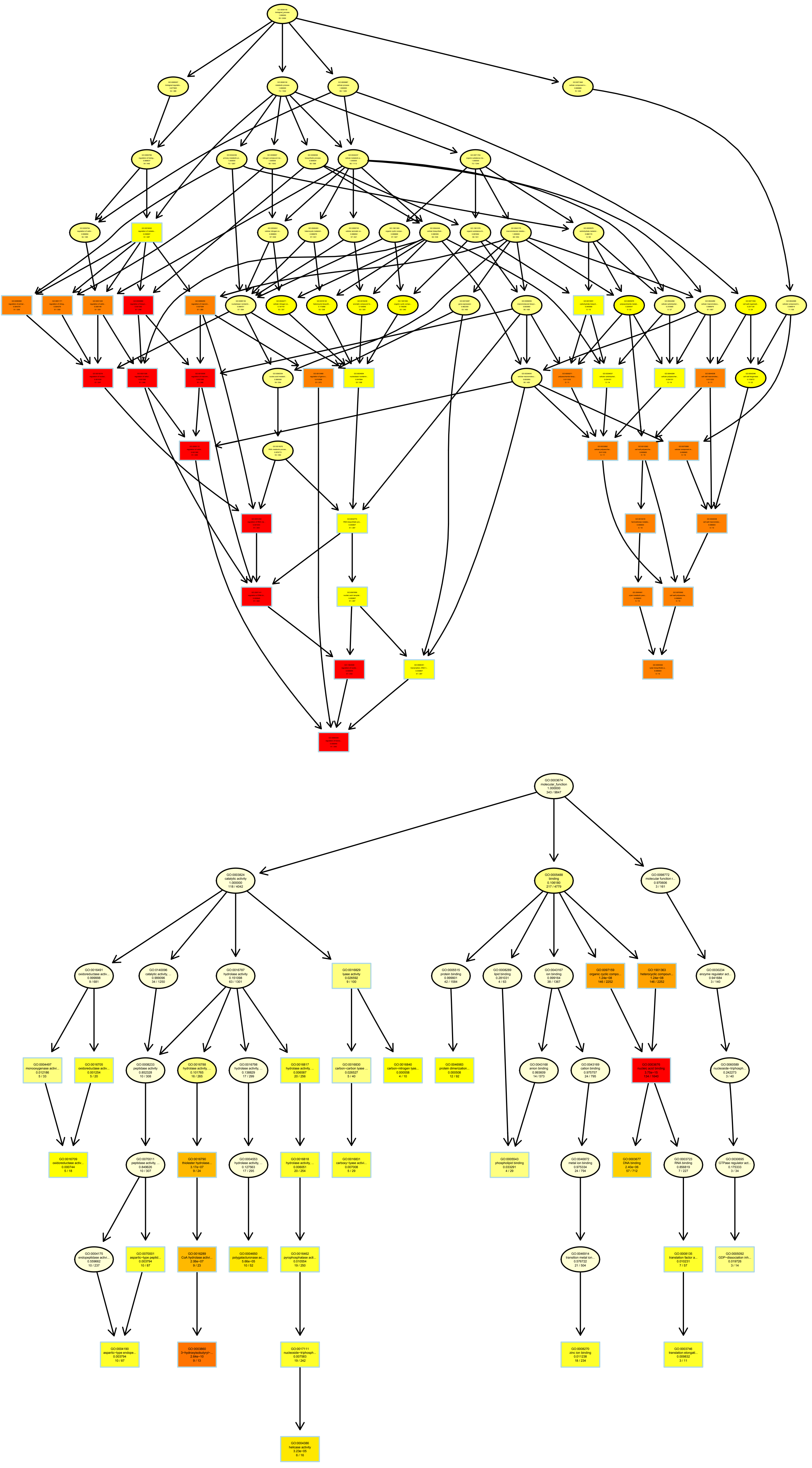

Figure S3 Gene ontology enrichment of *P. salicina*-expanded genes. Directed acyclic graph showed top enriched GO terms belonging to Category Biological Process (A) and Molecular Function (B). Rectangles indicate the significant terms with p-value < 0.01, with color ranging from dark red (represent most significant p-value) to bright yellow (least significant).The information displayed for each node, from first line to last line, is the GO term, GO name, p-value, and the number of all syntenic duplicates/ the number of total genes annotated to the respective GO term, respectively.

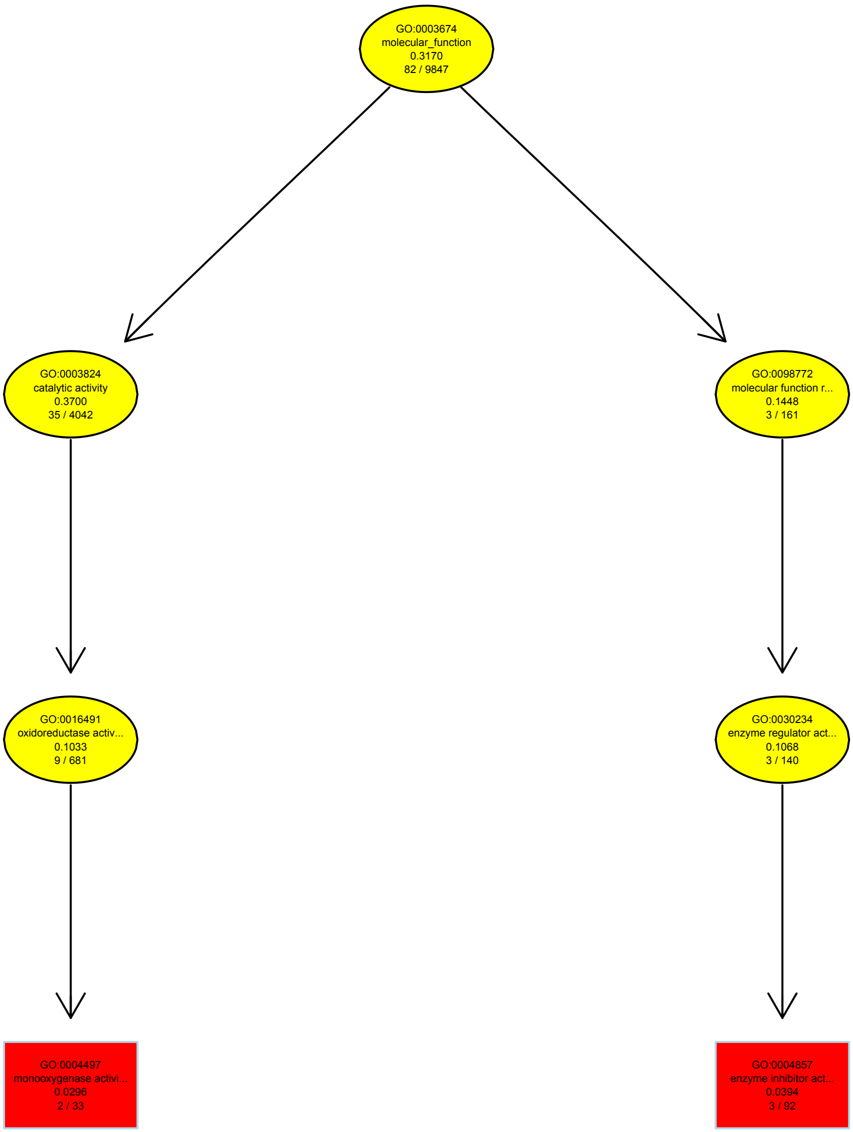

Figure S4 Gene ontology enrichment of the positively selected genes in *P. salicina*. Directed acyclic graph showed top enriched GO terms belonging to Category Molecular Function. Rectangles indicate the significant terms with p-value < 0.01, with color ranging from dark red (represent most significant p-value) to bright yellow (least significant).The information displayed for each node, from first line to last line, is the GO term, GO name, p-value, and the number of all syntenic duplicates/ the number of total genes annotated to the respective GO term, respectively.
