# Supplementary material for: Chromosome-level draft genome of a diploid plum (Prunus salicina)
Source: Gigascience. 2020 Dec 10;9(12):giaa130. doi: 10.1093/gigascience/giaa130 (PMC7727024; doi:10.1093/gigascience/giaa130)
Supplement: giaa130_Response_to_Reviewer_Comments_Original_Submission [file giaa130_response_to_reviewer_comments_original_submission.pdf]

Author's Response To Reviewer Comments

Close

Dear Editor and Reviewers,

We would like to thank you for helpful suggestions on our manuscript entitled "The chromosome-level draft genome of a diploid plum (Prunus salicina)" (GIGA-D-20-00195). Following the comments and suggestions, we rewrote the entire manuscript and re-organized the structure of the article. Our genome data have been submitted to Genome Database for Rosaceae (GDR) and received the accession number tfGDR1044. The reviewer's questions regarding heterozygosity, peach physical map and inter-specific hybrid were answered in detail. We carefully proofread the manuscript and corrected inappropriate words and expressions. We have studied the comments carefully and revised the manuscript according to reviewers' suggestions, and we expected that it would meet the publication requirement of GigaScience. A point by point response to the reviewers' comments and questions and the main corrections in the paper were provided below.

Reviewer reports:

--Reviewer #1:

This manuscript reports high-quality assembly and annotation for one Japanese plum (P. salicina) genome. Phylogenetic analysis has been performed based on the identification of orthologous genes. The genomic data are interesting and should be useful for the community, however, the authors do not put forward any clear research question and respective hypotheses. Therefore, the study will be of limited relevance for an international readership. Most importantly, I identified substantial shortcomings that cannot be alleviated on the basis of the data and analyses presented. The main points raised are summarized below:

The manuscript is poorly prepared. Material and Methods, Results and Discussion sections are not clearly identified and this does not help to estimate the scope and importance of the results presented. Review and discussion on published results in the similar topics and/or related species appeared insufficient. Material & Methods, Results, mixed with discussion, were not clearly presented. It is fine for results and discussion to be combined, but the results still should be presented first, clearly, then followed by relevant discussion. It also requires a proper Material & Methods section, even presented as supplemental information, but at least clearly identified from the results section. This paper needs substantial improvement of its content organization and clarity to be clear and understandable, before it could be re-submitted as a new manuscript. An alternative would be to present it as a short communication but the decision remains to the editorial board.

Response: Thanks for your suggestions. We are very sorry for the inconvenience due to the poor content organization. For the preparation of our original manuscript, we download several published papers in 'Data Note' section, and take them as reference to arrange the contents of our manuscript. We mainly focus on how to describe our data and ignore the content structures. According to the suggestions from you and editor, the content organizations are significantly improved, and the Methods and Results sections could be clearly identified in our revised manuscript. We hope the clear content structure could make it more convenient for your review.

The choice of the methodology for genome assembly is also raising question. Japanese plum is self-incompatible, at least in most accessions, and thus highly heterozygous. It is not clear how the authors disentangled the two expected haplotypes (therefore the two sets of 8 pseudomolecules for P. salicina). By the way, it is not clear why they assembled the accession 'Sanyueli', in particular. What is the level of heterozygosity in 'Sanyueli'.

Response: Thanks very much for your kindly suggestions.
(1) Assembling the highly heterozygous Japanese plum genome have long been challenging as a result

of its self-incompatible nature [1]. The short Illumina reads and even hybrid assembly strategies have always been problematic to de novo assemble any complex plant genome having highly heterozygous sequences. However, the problem has been greatly alleviated with the advent of new sequencing technologies as well as accompanying advances in genome assembly algorithms.

In recent years, the single-molecule, real-time (SMRT) PacBio sequencing and chromosome conformation capture (Hi-C) techniques have been used to make significant advances in improving the assembly of plant genomes at the chromosomal level. The PacBio sequencing can generate long reads which overcomes the restriction of the short reads generated from the Illumina sequencing platform [2]. The Hi-C technology has become available to generate reliable chromosome-scale de novo genome assemblies, and the Hi-C data can also be used to phase genome onto separate haplotypes at chromosomal-scale, since homologous chromosomes occupy distinct territories in nuclei, which could be used to distinguish different haplotypes [3].

Moreover, continuous optimizations for the genome assembly algorithms are helpful for us to disentangle the two expected haplotypes of Japanese plum genome. Just as you mentioned, the haplotype phasing is a key problem in heterozygous genome assemblies. The newer generation of genome assemblers, such as FALCON-Phase, Purge Haplotigs and FALCON-Unzip, are able to separate allelic contigs and have considerably improved the quality of highly heterozygous diploid genome [4]. In our study, the pipeline of 'Purge Haplotigs' [5] was used to remove the redundant sequences caused by genomic heterozygosity.

Based on the integration of PacBio sequencing, Hi-C technology and latest generation of genome assemblers, a series of high quality complex plant genomes have been obtained recently, such as the genome of rubber tree (heterozygosity rate of ~1.6%) [6], cushion willow (~0.71%) [7], Camellia sinensis var. sinensis (~1.22%) [8] and Durian (~1.14%) [9]. In our study, the level of heterozygosity for the Japanese plum 'Sanyueli' was about 0.7% (estimated by k-mer analysis), which was significantly lower than many published complex genomes. Therefore, we think it is not a major problem to assemble the Japanese plum genome and disentangle the two expected haplotypes.

(2)The accession 'Sanyueli' is an early-maturing and high-yielding Japanese plum variety and widely cultivated in South China. Besides the economic importance, 'Sanyueli' also has great value in breeding and scientific research for its lowest chilling requirements among the cultivated Japanese plum varieties [10]. Moreover, the preliminary genome survey results show that the heterozygosity rate (~0.7 %) of 'Sanyueli' is not very high. Therefore, 'Sanyueli' is selected for the subsequent genome sequencing and assembly in our study.

Authors used the peach physical map and genome assembly to align the metascaffolds onto 8 pseudo-molecules, corresponding to the eight haploid Prunus chromosomes. How did the authors handle the genomic re-arrangements (translocation, inversions, deletions) between peach and plum? Why didn't they use Japanese plum genetic maps which were previously published?

Response: Thank you very much for your kindly suggestions.
(1) We think there might be some misunderstandings, the peach physical map was not used in the genome assembly process in our study. The chromosome-level de novo genome assembly of Prunus salicina was generated using an integrated strategy that combined PacBio sequencing, Illumina sequencing and Hi-C technology. We used Hi-C to cluster and order contigs of this draft genome assembly into 8 pseudo-molecules, which cover ~96.56% of the total contig length. The genomic data from peach were only used as references in the gene annotation, orthogroup identification and phylogenetic analysis.

(2) Since the peach physical map and genome assembly were not used to align the scaffolds in our study, the genomic re-arrangements between peach and plum were not considered in the genome assembly process. For the Hi-C assisted assembly, we applied LACHESIS to cluster, order, and orient the assembly contigs onto pseudo-molecules.

(3) Genetic maps are useful tools for guiding scaffold anchoring into pseudo-chromosome assembly [11]. Up to now, only a few genetic linkage maps of Japanese plums have been reported [12-15], and there are still several problems in using them for the assisted genome assembly: ① Most of the parents are not local varieties of Japanese plums; ②The marker numbers and chromosome coverage are limited, and several large gaps are found; ③The original data for most of the genetic maps are not

available.

Moreover, the mapping algorithms used to build genetic maps can sometimes place markers at incorrect locations, which could lead to errors in the genome assembly [16]. The Hi-C technology employed in our study is a novel strategy combining capture of chromatin interaction within the nucleus and next-generation sequencing. Hi-C data can effectively identify linkage between contigs or scaffolds, allowing contigs being linked to nearly whole chromosome-scale [4]. This method has been widely used in many species and dramatically improved genome assemblies. For example, Jibran et al. [17] demonstrated that Hi-C analysis had vastly improved the black raspberry genome assembly, yielding a N50 contig size for the Hi-C guided assembly of 31,759,000 bp versus the N50 scaffold size of 48,488 bp for the previously genetic maps-assisted assembled genome of VanBuren et al. [18].

(4) Overall, compared to the published relatively low-density Japanese plum genetic maps, we think that the Hi-C technology has more advantages in the genome assembly. It is certain that the available high-density Japanese plum genetic maps could be used as an important supplement for the improvement of our genome assembly in the future.

P. salicina is inter-fertile with many other Prunus species, P. mume and P. armeniaca included, especially in China. This has been profoundly documented (see Zhang et al, 2018. DOI: 10.1038/s41467-018-04093-z). How did the authors check the fact that cv. 'Sanyueli' is pure Japanese plum and not an inter-specific hybrid?

Response: Thank you very much for your kindly suggestions.
(1) According to the paper you mentioned, there also might be introgression events in Japanese plum cultivars from Prunus species. The interspecific cross-compatibility is found among the diploid plum and non-plum species within the subgenus Prunophora [19]. Moreover, the diploid plums can also be hybridised with species from the subgenera Amygdalus (peach and almond) and Cerasus (cherry) but with less fertility [20]. Many interspecific hybrids have been reported and widely cultivated. For example, Prunus simonii might be a type of natural hybridization between P. salicina and P. armeniaca [21]; 'Santa Rosa' is a complex hybrid containing a mixture of P. salicina, P. saimonii, and P. Americana [22].

(2) 'Sanyueli' is a traditional landraces of Japanese plum and widely cultivated in South China, especially in Guangdong Province. 'Sanyueli' has long cultivation history and has been recorded in local gazetteers of Nanhua County in 1843 [23].

(3) Japanese plum originates in China, has a long cultivation history and wide geographical distribution ranging from the southern to the northern areas of the country. 'Sanyueli' is a low-chilling requirement and cold-sensitive Japanese plum variety, mainly distributed in the south of Japanese plum cultivation regions in China [10, 24].

According to the most widely accepted classification [25], Prunophora subgenus could be subdivided into the sections Euprunus (plum species native to Europe and Asia), Prunocerasus (plum species native to North America) and Armeniaca (apricot species). Among the species of Euprunus and Prunocerasus sections, only Japanese plum is widely found in South China region, according to the germplasm resources investigation [10]. Other plum species are not well adapted to the climate in South China, because the winter temperatures could not meet their chilling requirements for normal flowering in most years. The distribution characteristics of plums show that the natural outcrossing between 'Sanyueli' and other plum species in recent years might be considered as rare events.

Among the species in Section Armeniaca, only Prunus mume is also widely found in South China, which is overlapped with the distribution of ‘Sanyueli’. However, the differences in flowering time might reduce the possibility of natural outcrossing. As far as we know, there are no reports about the natural hybrids between Prunus mume and Prunus salicina. Boonprakob at al. [26] found that the P. mume produce semi-fertile hybrids in crosses with plum species. The interspecific hybrids between P. mume cv. Baigo and P. salicina cv. Sordum were created with manual hybridization by Hakoda et al. [27], and the hybrids can be easily distinguished with their parents according to the morphological characteristics like flower size and leaf shape.

(4) Overall, the above analyses indicate that the cv. 'Sanyueli' is most likely not from the recent interspecific hybridization. We think it could be a suitable candidate material for the Japanese plum

genome sequencing. However, we could not rule out the possibility of the introgression from other germplasms like P. mume during the long-term cultivation and domestication of 'Sanyueli'. In the future work, we will perform the whole-genome re-sequencing project for various germplams within Prunophora subgenus. We think the project will help us to better understand the genetic background of 'Sanyueli' and other varieties of Japanese plum.

Given those issues, the analyses appear rudimentary/descriptive and biased, the main conclusions not reliable enough and the previous studies on diversity and genetic studies in Japanese plums not taken into account.

Response: We agree that the analyses in our study maybe not comprehensive enough and the main conclusions need further experimental verification. However, our paper is submitted as a Data Note, which aims to incentivize and more rapidly release data before subsequent detailed analysis has been carried out, so we mainly focuses on presenting the genome data in our manuscript. We have actually noticed the previous studies on diversity and genetic studies in Japanese plum, and carefully selected cv. ‘Sanyueli’ for genome sequencing. We think that the completion of our high-quality Japanese plum genome will help to measure and characterize the genetic diversity and determine how this diversity relates to the tremendous phenotypic diversity among plum cultivars.

This situation is aggravated by the fact that in many instances, writing is not clear and terminology inappropriate, with many awkward or incorrect sentences (for ex. In the abstract, what does 'hold the center of the Prunus' mean or what is a 'typical' diploid plum species for the authors?). Attention should be given to using correct terms. A substantial English proofreading is required.

Response: Thank you very much for your kindly suggestions. We are sorry for the unclear writing and inappropriate terminology in our original manuscript. We reorganize the article structures and carefully modify the incorrect sentences that you pointed out. The substantial English proofreading is implemented, and the inappropriate words and expressions are corrected in revised manuscript.

Reviewer #2:
The authors report the first chromosome-level genome assembly of plum (P. salicina), which is an economically important fruit crop and therefore provide a useful resource for the research community of this fruit tree. They also provided a phylogenetic analysis with P. nume and P. armenica and studied gene family expansion in P. salicina evolution investigating in particular xylan metabolism which might have an impact on fruit quality.
I believe that the paper is well written and provides a useful resource for the community therefore I would welcome its publication once a few, mostly minor, issues are addressed.

I have seen that the data is/will be available on public repositories but I did not see the assembled sequences and the usual services like BLAST that would make the genome truly available for the community. I am not sure whether authors intend to publish this data on their own web-server alongside GigaDB, but I would also recommend to submit sequences/gene predictions to specialized databases like the Genome Database for Rosaceae (GDR) which will make this data easily available for the rosaceae community.

Response: According to your suggestion, we have submitted our genome data to GDR and received the accession number tfGDR1044. The genome data will be available through the link https://www.rosaceae.org/publication_datasets. (Line 435)

Detailed comments

line 31: "Plums are the economically important" I believe should be "Plums are one of the most economically important... and are produced"
Response: We have corrected it according to your suggestion. (Line 31)

line 64: originate should be originates
Response: We corrected accordingly. (Line 63)

line 88: some references here are missing like Daccord et.al, 2017 for the apple GDDH13 genome and Linsmith et al, 2019 for European Pear. The published genomes of Prunus avium, Prunus armenica and Prunus dulcis are also ignored here. I am not an expert in Prunus, but perhaps authors should also consider providing a collinearity analysis with avium and dulcis.
Response: Thanks very much for your kindly suggestions. The references you mentioned have been added in the revised manuscript (Apple GDDH13, Ref 15; European Pear, Ref 17; Sweet cherry, Ref 27; Apricot, Ref 29; Almond, Ref 24)(Line 88-89).According to your suggestion, the collinearity analysis between P. salicina, P. avium and P. dulcis was performed in revised manuscript (Figure 3B, Line 375-379).

line 105: conversation should be conservation
Response: We corrected accordingly. (Line 108)

line 119: I guess that by "with unknown bases (N) than 10%" authors mean "with more than 10% unknown bases (N)", and with more than 50% low quality bases... Please rephrase.
Response: We rephrase the sentence according to your suggestion. (Line 124)

line 145: "were used to estimate the genomic information" I would rephrase this to say that they were used to perform a kmer analysis to estimate the genome size.
Response: Thanks very much for your kindly suggestions. We corrected accordingly. (Line 141)

lines 156-158: this is what FALCON does, so in my opinion there is no need to repeat this here.
Response: We corrected accordingly. (Line 146)

line 189: I would remove approaches.
Response: We corrected accordingly. (Line 174)

line 194: In table 1 it would be interesting to have more information on CEGMA and BUSCO like the % of duplicated genes vs unique etc. which are in the supplementary material
Response: According to your suggestions, more detailed information about CEGMA and BUSCO were added in Table 1.

line 195: It would be interesting to see how many telomeric sequences are recovered at each end of the assembled chromosomes to show how complete they are. I believe this could be a nice addition to this paragraph.
Response: Thanks very much for your kindly suggestions. According to your suggestions, the telomere sequences were identified by BLASTN searches using tandem repeats of the telomere repeat motif (TTTAGGG), and the results were exhibited in Table S5.

#line 211: remove "of"
Response: We corrected accordingly. (Line 342)

#line 213-214: any comment on why transferase activity and phloem development were enriched?
Response: Thanks very much for your kindly suggestions. The possible causes for the significant enrichment of sieve element occlusion genes in 'phloem development' were discussed in revised manuscript. (Line 348-350)

#line 221-222: maybe authors should have added the protein sequences from Pyrus Communis as well. In the gene family identification paragraph Pyrus communis is actually mentioned, therefore this might just be an oversight here.
Response: Thanks very much for your kindly suggestions. Sequences from Prunus salicina and other 16 sequenced rosids species, including Pyrus Communis, were actually used in the gene family identification (Line 240). However, only 7 species (Prunus persica, Prunus avium, Prunus mume, Pyrus bretschneideri, Malus domestica, Fragaria vesca and Arabidopsis thaliana) were selected in the homology-based gene prediction (Line 198-199). The Pyrus Communis was not included because the Pyrus bretschneideri was selected as the representative of pear.

#line 224: SwisssProt should be SwissProt
Response: We corrected accordingly. (Line 220)

#Lines 245-247: It is not clear to me if authors used only Interpro results to annotate the plum proteins

with the Gene Ontology? In this case, why did they also perform the BLAST search against NR and SwissProt? Otherwise, how did they use the BLAST results to retrieve the GO terms? Please explain.
Response: We only used the Interpro results to annotate the Japanese plum proteins with the Gene Ontology (GO). The GO IDs for each gene were assigned according to the corresponding InterPro entry. The InterPro database, which includes 14 member databses, integrates diverse information about protein families, domains and functional sites [28]. The InterPro databases group one or more related member databases signatures, and provides additional overarching functional annotations, including GO terms wherever possible.The BLAST search against NR and SwissProt databases were also performed in our study, because they were not integrated into the InterPro databases and had different focuses and distinctive signatures. The NR dataset include the non-redundant protein sequences from GenPept, SwissProt, PIR, PDF, PDB, and NCBI Refseq, and the annotations might be more comprehensive. SwissProt is a curated protein sequence database [29], which might be able to provide the high quality annotation.

#Figure 2: The quality of the figure I saw is quite low and it is difficult to read the names. This might be due to the pdf version I have seen, but please double-check
Response: We checked the quality of Figure 2 again, and found that the low figure quality was due to the PDF version that you have seen. The original figure in TIFF format could be downloaded through the link “Click here to access/download” at the top right corner of the PDF pages.

#Figure 3: P. armeniaeca should be P. armenica
Response: According to your suggestion, we corrected the scientific name of apricot (in Figure 3) to P. armeniaca.

Reference
1. Guerra M and Rodrigo J. Japanese plum pollination: A review. Sci Hortic 2015; 197:674-686.
2. Wei S, Yang Y and Yin T. The chromosome-scale assembly of the willow genome provides insight into Salicaceae genome evolution. Hortic Res 2020; 7(1):1-12.
3. Korbel JO and Lee C. Genome assembly and haplotyping with Hi-C. Nat Biotechnol 2013; 31 (12):1099-1101.
4. Zhang X, Wu R, Wang Y, Yu J and Tang H. Unzipping haplotypes in diploid and polyploid genomes. Comput Struct Biotechnol J 2020; 18:66-72.
5. Roach MJ, Schmidt SA and Borneman AR. Purge Haplotigs: allelic contig reassignment for third-gen diploid genome assemblies. BMC Bioinformatics 2018; 19 (1):460.
6. Liu J, Shi C, Shi CC, Li W, Zhang QJ, Zhang Y, et al. The Chromosome-Based Rubber Tree Genome Provides New Insights into Spurge Genome Evolution and Rubber Biosynthesis. Mol Plant 2020; 13 (2):336-350.
7. Chen JH, Huang Y, Brachi B, Yun QZ, Zhang W, Lu W, et al. Genome-wide analysis of Cushion willow provides insights into alpine plant divergence in a biodiversity hotspot. Nat Commun 2019; 10 (1):5230.
8. Zhang Q-J, Li W, Li K, Nan H, Shi C, Zhang Y, et al. SMRT sequencing yields the chromosome-scale reference genome of tea tree, Camellia sinensis var. sinensis. BioRxiv 2020.
9. Teh BT, Lim K, Yong CH, Ng CCY, Rao SR, Rajasegaran V, et al. The draft genome of tropical fruit durian (Durio zibethinus). Nat Genet 2017; 49 (11):1633-1641.
10. He Yehua, Feng Junting, Guo Cuihong, Han Jingzhong, Xie Zhiliang and Yu Xiaolin. Study on plum germplasm of Guangdong (In Chinese). Acta Horticulturae Sinica 2014; 41(S):2610.
11. Xue H, Wang S, Yao JL, Deng CH, Wang L, Su Y, et al. Chromosome level high-density integrated genetic maps improve the Pyrus bretschneideri 'DangshanSuli' v1.0 genome. BMC Genomics 2018; 19 (1):833.
12. Vieira EA, Nodari, RO, de Mesquita Dantas A C, Ducroquet JPHJ, Dalbó M, & Borges, CV. Genetic mapping of Japanese plum. Crop Breed Appl Biot 2005; 5(1):29-37.
13. Salazar JA, Pacheco I, Shinya P, Zapata P, Silva C, Aradhya M, et al. Genotyping by Sequencing for SNP-Based Linkage Analysis and Identification of QTLs Linked to Fruit Quality Traits in Japanese Plum (Prunus salicina Lindl.). Front Plant Sci 2017; 8:476.
14. Carrasco B, Gonzalez M, Gebauer M, Garcia-Gonzalez R, Maldonado J and Silva H. Construction of a highly saturated linkage map in Japanese plum (Prunus salicina L.) using GBS for SNP marker calling. PLoS One 2018; 13 (12):e0208032.
15. Zhang Q-p, Wei X, Liu N, Zhang Y-p, Xu M, Zhang Y-j, et al. Construction of an SNP-based high-density genetic map for Japanese plum in a Chinese population using specific length fragment sequencing. Tree Genetics & Genomes 2020; 16(1):1-10.
16. Tang H, Zhang X, Miao C, Zhang J, Ming R, Schnable JC, et al. ALLMAPS: robust scaffold ordering based on multiple maps. Genome Biol 2015; 16 (1):3.
17. Jibran R, Dzierzon H, Bassil N, Bushakra JM, Edger PP, Sullivan S, et al. Chromosome-scale

scaffolding of the black raspberry (Rubus occidentalis L.) genome based on chromatin interaction data. Hortic Res 2018; 5(1): 1-11.
18. VanBuren R, Bryant D, Bushakra JM, Vining KJ, Edger PP, Rowley ER, et al. The genome of black raspberry (Rubus occidentalis). Plant J 2016; 87 (6):535-547.
19. Okie W and Weinberger J. Fruit breeding, volume I: Tree and tropical fruits. John Wiley & Sons, Inc., New York, 1996.
20. Topp BL, Russell DM, Neumüller M, Dalbó MA and Liu W. Plum. In: Maria Luisa Badenes DHB, editor. Fruit Breeding. Springer; 2012.p.571-621.
21. Faust M and Surányi D. Origin and dissemination of plums. Hort Rev. 1999; 23: 179-231.
22. Karp D. Luther Burbank's plums. HortScience 2015; 50(2):189-194.
23. Zhang Jiayan and Zhou En. Records of Chinese fruit-Journal of plum (In Chinese). Beijing: China Forestry Publishing House, 1998. p.17-23.
24. Liu Shuo, Xu Ming, Zhang Yuping, Zhang Yujun, Ma Xiaoxue, Zhang Qiuping, Liu Ning, Liu Weisheng. Retrospect, problematical issues and the prospect of plum breeding in China (In Chinese). Journal of Fruit Science 2018; 35 (2):231-245.
25. Rehder A. Manual of cultivated trees and shrubs. 1949.
26. Boonprakob U and Byrne D. Mume, a possible source of genes in apricot breeding. Fruit Varieties J 1990; 44 (3):108-113.
27. Hakoda N, Toyoda R, Tabuchi T, Ogiwara I, Ishikawa S and Shimura I. Morphological characteristics of the interspecific hybrids between Japanese apricot (Prunus mume) and Plum (P. salicina) (In Japanese). J JPN SOC HORTIC SCI 1998; 67 (5):708-714.
28. Finn RD, Attwood TK, Babbitt PC, Bateman A, Bork P, Bridge AJ, et al. InterPro in 2017-beyond protein family and domain annotations. Nucleic Acids Res 2017; 45 (D1): D190-D199.
29. Gasteiger E, Jung E and Bairoch AM. SWISS-PROT: connecting biomolecular knowledge via a protein database. Curr Issues Mol Biol 2001; 3 (3):47-55.

Close
