# Supplementary material for: Chromosome-level draft genome of a diploid plum (Prunus salicina)
Source: Gigascience. 2020 Dec 10;9(12):giaa130. doi: 10.1093/gigascience/giaa130 (PMC7727024; doi:10.1093/gigascience/giaa130)
Supplement: giaa130_Response_to_Reviewer_Comments_Revision_1 [file giaa130_response_to_reviewer_comments_revision_1.pdf]

Author's Response To Reviewer Comments

Close

Dear Editor and Reviewer,

We would like to thank you for helpful suggestions on our manuscript entitled "The chromosome-level draft genome of a diploid plum (Prunus salicina)" (GIGA-D-20-00195R1). Following the comments and suggestions, we corrected the numbering of the P. salicina pseudochromosomes all over the manuscript. The P. salicina pseudo-chromosome names and gene IDs in Figure 1, 3 and 4 were modified in the revised manuscript. We also contacted the staff of GDR (Genome Database for Rosaceae) and GenBank to update our genomic information. We expected that it would meet the publication requirement of GigaScience.

A point by point response to the reviewer' comments and questions and the main corrections in the paper were provided below.

Reviewer #1: I thank the authors for further revising their manuscript and clarifying some outstanding issues in regards of English proofreading and MS layout. Thank you very much for the answers to my previous questions, even if I do not fully agree with soem of them.
However, there is still a major revision necessary before the manuscript is ready for publication. I bet I overlooked it in the first version of the manuscript because of the other issues that were since corrected. My main concern relates to the chromosome nomenclature: the chromosome numbering is not in adequation with the Prunus genetic map. For exemple, Chromosome 1 in all Prunus species is always the largest one and following Figure 1, it appears that it is chromosome 2, here. The same remark applies to the other chromosomes, not only chromosome 2 (see figure 2B, chromosome 1 of P. salicina should in fact be chromosome 6 in the Prunus genetic map, chr3 should be chr4 and so on), and that's the reason why I was recommending using, even a few, Prunus genetic markers, to correct this discrepancy. This major issue is coming from the first release of the P. mume genome in 2012 and was reproduced in the P. armeniaca genome presented here. If colinearity has to be displayed (Figure 3) then it should be made clear that Chr2 here should be in fact Chr 1 in the genetic map. In fact, I would once again recommend the authors to re-order their chromosomes, according to the general acknowledged genetic map. Since the genetic maps were obtained by using molecular markers which are largely colinear and syntenic in between Prunus species (peach, P. mume, apricot and plum included) I would strongly recommend to right this issue, both within the P. salicina assembly and the following colinearity studies with the other genomes. Since genetic maps were released before genome assembly, the authors are expected to follow the internationally acknowledged nomenclature. Reproducing forever the mistake made initially for the P. mume genome would severely limit the interest of this de novo assembled genome and thus the impact of its release.
In conclusion, I recommend the authors to correct the numbering of the P. salicina chromosome all over the MS (by using a few of plum markers and even better Prunus orthologous markers as published in https://doi.org/10.1371/journal.pone.0208032, for that they only need to do a ePCR with markers depicted in Table S2F) and the data available online (and therefore Figure 3, accordingly).

Response：Thank you very much for your kindly suggestion. We further carefully read the literatures that you mentioned, and realized that the important role of the Prunus genetic maps was ignored in our first version of the manuscript. According to your kind advices, the numbering of the P. salicina pseudochromosome was corrected in the revised manuscript, using the markers that you recommended.

The present pseudochromosome numbering is consisted with that in the published P. salicina genetic map (Ref 77 in the revised manuscript). The P. salicina pseudo-chromosomes names and gene IDs in Figure 1, 3 and 4 were modified in the revised manuscript. The detailed information about the P. salicina pseudochromosomes in Table S5 was corrected accordingly. Moreover, we also contacted the staff of GDR (Genome Database for Rosaceae) and GenBank to update our genomic information.

Close
