# Supplementary material for: Chromosome-level draft genome of a diploid plum (Prunus salicina)
Source: Gigascience. 2020 Dec 10;9(12):giaa130. doi: 10.1093/gigascience/giaa130 (PMC7727024; doi:10.1093/gigascience/giaa130)
Supplement: giaa130_Reviewer_1_Report_Original_Submission — Veronique Decroocq, Ph.D -- 7/20/2020 Reviewed [file giaa130_reviewer_1_report_original_submission.pdf]

Reviewer Report

Title: The chromosome-level draft genome of a diploid plum (Prunus salicina)

Version: Original Submission **Date:** 7/20/2020

Reviewer name: Veronique Decroocq, Ph.D

Reviewer Comments to Author:

This manuscript reports high-quality assembly and annotation for one Japanese plum (P. salicina) genome. Phylogenetic analysis has been performed based on the identification of orthologous genes. The genomic data are interesting and should be useful for the community, however, the authors do not put forward any clear research question and respective hypotheses. Therefore, the study will be of limited relevance for an international readership.

Most importantly, I identified substantial shortcomings that cannot be alleviated on the basis of the data and analyses presented. The main points raised are summarized below:

- The manuscript is poorly prepared. Material and Methods, Results and Discussion sections are not clearly identified and this does not help to estimate the scope and importance of the results presented. Review and discussion on published results in the similar topics and/or related species appeared insufficient. Material & Methods, Results, mixed with discussion, were not clearly presented. It is fine for results and discussion to be combined, but the results still should be presented first, clearly, then followed by relevant discussion. It also requires a proper Material & Methods section, even presented as supplemental information, but at least clearly identified from the results section. This paper needs substantial improvement of its content organization and clarity to be clear and understandable, before it could be re-submitted as a new manuscript. An alternative would be to present it as a short communication but the decision remains to the editorial board.
- The choice of the methodology for genome assembly is also raising question. Japanese plum is self-incompatible, at least in most accessions, and thus highly heterozygous. It is not clear how the authors disentangled the two expected haplotypes (therefore the two sets of 8 pseudomolecules for P. salicina). By the way, it is not clear why they assembled the accession 'Sanyueli', in particular. What is the level of heterozygosity in 'Sanyueli'.
- Authors used the peach physical map and genome assembly to align the metascaffolds onto 8 pseudo-molecules, corresponding to the eight haploid Prunus chromosomes. How did the authors handle the genomic re-arrangements (translocation, inversions, deletions) between peach and plum? Why didn't they use Japanese plum genetic maps which were previously published?
- P. salicina is inter-fertile with many other Prunus species, P. mume and P. armeniaca included, especially in China. This has been profoundly documented (see Zhang et al, 2018. DOI: 10.1038/s41467-018-04093-z). How did the authors check the fact that cv. 'Sanyueli' is pure Japanese plum and not an inter-specific hybrid ?

Given those issues, the analyses appear rudimentary/descriptive and biased, the main conclusions not reliable enough and the previous studies on diversity and genetic studies in Japanese plums not taken into account.

This situation is aggravated by the fact that in many instances, writing is not clear and terminology inappropriate, with many awkward or incorrect sentences (for ex. In the abstract, what does 'hold the center of the Prunus' mean or what is a 'typical' diploid plum species for the authors ?). Attention should be given to using correct terms. A substantial English proofreading is required.

Level of Interest

Please indicate how interesting you found the manuscript: Choose an item.

Quality of Written English

Please indicate the quality of language in the manuscript: Choose an item.

Declaration of Competing Interests

Please complete a declaration of competing interests, considering the following questions:

- Have you in the past five years received reimbursements, fees, funding, or salary from an organisation that may in any way gain or lose financially from the publication of this manuscript, either now or in the future?
- Do you hold any stocks or shares in an organisation that may in any way gain or lose financially from the publication of this manuscript, either now or in the future?
- Do you hold or are you currently applying for any patents relating to the content of the manuscript?
- Have you received reimbursements, fees, funding, or salary from an organization that holds or has applied for patents relating to the content of the manuscript?
- Do you have any other financial competing interests?
- Do you have any non-financial competing interests in relation to this paper?

If you can answer no to all of the above, write 'I declare that I have no competing interests' below. If your reply is yes to any, please give details below.

I declare that I have no competing interests

I agree to the open peer review policy of the journal. I understand that my name will be included on my report to the authors and, if the manuscript is accepted for publication, my named report including any attachments I upload will be posted on the website along with the authors' responses. I agree for my report to be made available under an Open Access Creative Commons CC-BY license (http://creativecommons.org/licenses/by/4.0/). I understand that any comments which I do not wish to be included in my named report can be included as confidential comments to the editors, which will not be published.

Choose an item.

To further support our reviewers, we have joined with Publons, where you can gain additional credit to further highlight your hard work (see: https://publons.com/journal/530/gigascience). On publication of this paper, your review will be automatically added to Publons, you can then choose whether or not to claim your Publons credit. I understand this statement.

Yes Choose an item.
