# Supplementary material for: Chromosome-level draft genome of a diploid plum (Prunus salicina)
Source: Gigascience. 2020 Dec 10;9(12):giaa130. doi: 10.1093/gigascience/giaa130 (PMC7727024; doi:10.1093/gigascience/giaa130)
Supplement: giaa130_Reviewer_1_Report_Revision_1 — Veronique Decroocq, Ph.D -- 9/20/2020 Reviewed [file giaa130_reviewer_1_report_revision_1.pdf]

Reviewer Report

Title: The chromosome-level draft genome of a diploid plum (Prunus salicina)

Version: Revision 1 **Date:** 9/20/2020

Reviewer name: Veronique Decroocq, Ph.D

Reviewer Comments to Author:

I thank the authors for further revising their manuscript and clarifying some outstanding issues in regards of English proofreading and MS layout. Thank you very much for the answers to my previous questions, even if I do not fully agree with soem of them.
However, there is still a major revision necessary before the manuscript is ready for publication. I bet I overlooked it in the first version of the manuscript because of the other issues that were since corrected. My main concern relates to the chromosome nomenclature: the chromosome numbering is not in adequation with the Prunus genetic map. For exemple, Chromosome 1 in all Prunus species is always the largest one and following Figure 1, it appears that it is chromosome 2, here. The same remark applies to the other chromosomes, not only chromosome 2 (see figure 2B, chromosome 1 of P. salicina should in fact be chromosome 6 in the Prunus genetic map, chr3 should be chr4 and so on), and that's the reason why I was recommending using, even a few, Prunus genetic markers, to correct this discrepancy. This major issue is coming from the first release of the P. mume genome in 2012 and was reproduced in the P. armeniaca genome presented here. If colinearity has to be displayed (Figure 3) then it should be made clear that Chr2 here should be in fact Chr 1 in the genetic map. In fact, I would once again recommend the authors to re-order their chromosomes, according to the general acknowledged genetic map. Since the genetic maps were obtained by using molecular markers which are largely colinear and syntenic in between Prunus species (peach, P. mume, apricot and plum included) I would strongly recommend to right this issue, both within the P. salicina assembly and the following colinearity studies with the other genomes. Since genetic maps were released before genome assembly, the authors are expected to follow the internationally acknowledged nomenclature. Reproducing for ever the mistake made initially for the P. mume genome would severely limit the interest of this de novo assembled genome and thus the impact of its release.
In conclusion, I recommend the authors to correct the numbering of the P. salicina chromosome all over the MS (by using a few of plum markers and even better Prunus orthologous markers as published in https://doi.org/10.1371/journal.pone.0208032, for that they only need to do a ePCR with markers depicted in Table S2F) and the data available online (and therefore Figure 3, accordingly).

Level of Interest

Please indicate how interesting you found the manuscript: Choose an item.

Quality of Written English

Please indicate the quality of language in the manuscript: Choose an item.

Declaration of Competing Interests

Please complete a declaration of competing interests, considering the following questions:

- Have you in the past five years received reimbursements, fees, funding, or salary from an organisation that may in any way gain or lose financially from the publication of this manuscript, either now or in the future?
- Do you hold any stocks or shares in an organisation that may in any way gain or lose financially from the publication of this manuscript, either now or in the future?
- Do you hold or are you currently applying for any patents relating to the content of the manuscript?
- Have you received reimbursements, fees, funding, or salary from an organization that holds or has applied for patents relating to the content of the manuscript?
- Do you have any other financial competing interests?
- Do you have any non-financial competing interests in relation to this paper?

If you can answer no to all of the above, write 'I declare that I have no competing interests' below. If your reply is yes to any, please give details below.

I declare that I have no competing interests

I agree to the open peer review policy of the journal. I understand that my name will be included on my report to the authors and, if the manuscript is accepted for publication, my named report including any attachments I upload will be posted on the website along with the authors' responses. I agree for my report to be made available under an Open Access Creative Commons CC-BY license (http://creativecommons.org/licenses/by/4.0/). I understand that any comments which I do not wish to be included in my named report can be included as confidential comments to the editors, which will not be published.

Choose an item.

To further support our reviewers, we have joined with Publons, where you can gain additional credit to further highlight your hard work (see: https://publons.com/journal/530/gigascience). On publication of this paper, your review will be automatically added to Publons, you can then choose whether or not to claim your Publons credit. I understand this statement.

Yes Choose an item.
