# Supplementary material for: Chromosome-level draft genome of a diploid plum (Prunus salicina)
Source: Gigascience. 2020 Dec 10;9(12):giaa130. doi: 10.1093/gigascience/giaa130 (PMC7727024; doi:10.1093/gigascience/giaa130)
Supplement: giaa130_Reviewer_2_Repor_Original_Submission — Luca Bianco, Ph.D -- 7/22/2020 Reviewed [file giaa130_reviewer_2_repor_original_submission.pdf]

Reviewer Report

Title: The chromosome-level draft genome of a diploid plum (Prunus salicina)

Version: Original Submission **Date:** 7/22/2020

Reviewer name: Luca Bianco, Ph.D

Reviewer Comments to Author:

The authors report the first chromosome-level genome assembly of plum (P. salicina), which is an economically important fruit crop and therefore provide a useful resource for the research community of this fruit tree. They also provided a phylogenetic analysis with P. nume and P. armenica and studied gene family expansion in P.salicina evolution investigating in particular xylan methabolism which might have an impact on fruit quality.
I believe that the paper is well written and provides a useful resource for the community therefore I would welcome its publication once a few, mostly minor, issues are addressed. I have seen that the data is/will be available on public repositories but I did not see the assembled sequences and the usual services like BLAST that would make the genome truly available for the community. I am not sure whether authors intend to publish this data on their own web-server alongside GigaDB, but I would also recommend to submit sequences/gene predictions to specialized databases like the Genome Database for Rosaceae (GDR) which will make this data easily available for the rosaceae community.
Detailed comments
line 31: "Plums are the economically important" I believe should be "Plums are one of the most economically important... and are produced"
line 64: originate should be originates
line 88: some references here are missing like Daccord et.al, 2017 for the apple GDDH13 genome and Linsmith et al, 2019 for European Pear. The published genomes of Prunus avium, Prunus armenica and Prunus dulcis are also ignored here. I am not an expert in Prunus, but perhaps authors should also consider providing a collinearity analysis with avium and dulcis.
line 105: conversation should be conservation
line 119: I guess that by "with unknown bases (N) than 10%" authors mean "with more than 10% unknown bases (N)", and with more than 50% low quality bases... Please rephrase.
line 145: "were used to estimate the genomic information" I would rephrase this to say that they were used to perform a kmer analysis to estimate the genome size.
lines 156-158: this is what FALCON does, so in my opinion there is no need to repeat this here.
line 189: I would remove approaches.
line 194: In table 1 it would be interesting to have more information on CEGMA and BUSCO like the % of duplicated genes vs unique etc. which are in the supplementary material
line 195: It would be interesting to see how many telomeric sequences are recovered at each end of the assembled chromosomes to show how complete they are. I believe this could be a nice addition to this paragraph.
line 211: remove "of"

line 213-214: any comment on why transferase activity and phloem development were enriched?
line 221-222: maybe authors should have added the protein sequences from Pyrus Communis as well. In the gene family identification paragraph Pyrus communis is actually mentioned, therefore this might just be an oversight here.
line 224: SwisssProt should be SwissProt
Lines 245-247: It is not clear to me if authors used only Interpro results to annotate the plum proteins with the Gene Ontology? In this case, why did they also perform the BLAST search against NR and SwissProt? Otherwise, how did they use the BLAST results to retrieve the GO terms? Please explain.
Figure 2: The quality of the figure I saw is quite low and it is difficult to read the names. This might be due to the pdf version I have seen, but please double-check
Figure 3: P. armeniaeca should be P. armenica

Level of Interest

Please indicate how interesting you found the manuscript: Choose an item.

Quality of Written English

Please indicate the quality of language in the manuscript: Choose an item.

Declaration of Competing Interests

Please complete a declaration of competing interests, considering the following questions:

- Have you in the past five years received reimbursements, fees, funding, or salary from an organisation that may in any way gain or lose financially from the publication of this manuscript, either now or in the future?
- Do you hold any stocks or shares in an organisation that may in any way gain or lose financially from the publication of this manuscript, either now or in the future?
- Do you hold or are you currently applying for any patents relating to the content of the manuscript?
- Have you received reimbursements, fees, funding, or salary from an organization that holds or has applied for patents relating to the content of the manuscript?
- Do you have any other financial competing interests?
- Do you have any non-financial competing interests in relation to this paper?

If you can answer no to all of the above, write 'I declare that I have no competing interests' below. If your reply is yes to any, please give details below.

I declare that I have no competing interests

I agree to the open peer review policy of the journal. I understand that my name will be included on my report to the authors and, if the manuscript is accepted for publication, my named report including any attachments I upload will be posted on the website along with the authors' responses. I agree for my report to be made available under an Open Access Creative Commons CC-BY license (http://creativecommons.org/licenses/by/4.0/). I understand that any comments which I do not wish to be included in my named report can be included as confidential comments to the editors, which will not be published.

Choose an item.

To further support our reviewers, we have joined with Publons, where you can gain additional credit to further highlight your hard work (see: https://publons.com/journal/530/gigascience). On publication of this paper, your review will be automatically added to Publons, you can then choose whether or not to claim your Publons credit. I understand this statement.

Yes Choose an item.
